# Sulfur, mercury, and boron adducts of sydnone imine derived anionic N-heterocyclic carbenes†

**DOI:** 10.1039/c9ra00294d

**Published:** 2019-02-06

**Authors:** Tyll Freese, Jan C. Namyslo, Martin Nieger, Andreas Schmidt

**Affiliations:** Clausthal University of Technology, Institute of Organic Chemistry Leibnizstrasse 6, Clausthal-Zellerfeld D-38678 Germany schmidt@ioc.tu-clausthal.de; Department of Chemistry, University of Helsinki P. O. Box 55 FIN-00014 Finland

## Abstract

The sydnone imines (5-benzoylimino)-3-(2-methoxyphenyl)-sydnone imine and molsidomine were deprotonated at C4 to give sydnone imine anions which can be represented as anionic N-heterocyclic carbenes, respectively. Trapping reactions with sulfur gave unstable sydnone imine sulfides which were stabilized by the formation of methyl thioethers, methyl sulfoxides, gold complexes [(PPh_3_)Au–S-sydnone imine] and a bis(ligand) mercury(ii) complex. The latter possesses a tetrahedral coordination of the mercury central atom to the sulfur atoms with the N6 nitrogen atoms coordinating as neutral ligands. Water converted the molsidomine anion into ethyl(2-morpholino-2-thioxoacetyl)carbamate. Mercury(ii)chloride and triphenylborane were employed to trap the sydnone imine carbenes as mercury complexes as well as BPh_3_ adducts.

## Abstract 2

The sydnone imines (5-benzoylimino)-3-(2-methoxyphenyl)-sydnone imine and molsidomine were deprotonated at C4 to give sydnone imine anions which can be represented as anionic N-heterocyclic carbenes, respectively.

## Graphical abstract

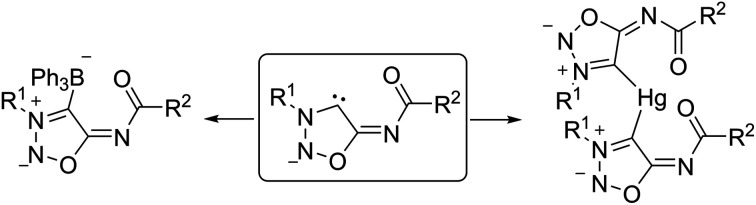

## Introduction

**Scheme 1:**
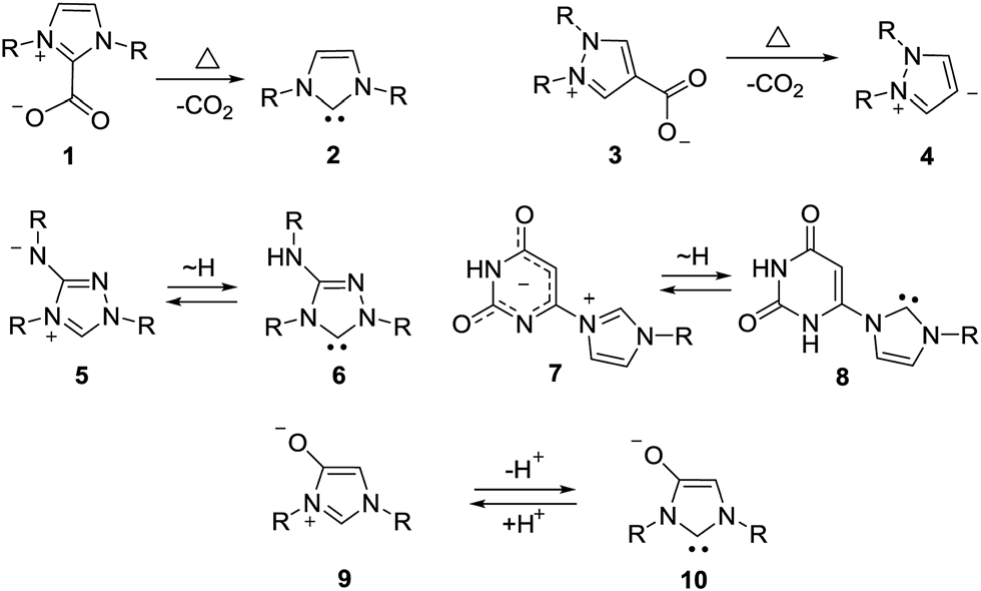
Examples of mesomeric betaine – N-heterocyclic carbene transformations.

Mesomeric betaines (MB) can exclusively be represented by several zwitterionic canonical forms in which the positive and negative charges are delocalized within a common π-electron system. They have proven to be versatile starting materials for the generation of N-heterocyclic carbenes (NHC) as well as of their anionic derivatives^1^ which play important roles in synthesis and catalysis. According to a recent classification which is based on a matrix-connectivity analysis, five distinct classes of mesomeric betaines can be differentiated.^2^ As examples, conjugated (CMB), cross-conjugated (CCMB) and pseudo-cross-conjugated mesomeric betaines (PCCMB) differ in their charge distribution with respect to the canonical formulae.^3^ In addition, characteristic dipole types of each class can be dissected from the mesomeric structures.^3^ Conjugated and cross-conjugated mesomeric betaines differ also in their frontier orbital profiles.^4^ Not unexpectedly, the distinct types of mesomeric betaines show different chemical behaviours which is also reflected in their potential transformations into distinct types of N-heterocyclic carbenes.^1,5^ Thus, pseudo-cross-conjugated mesomeric betaines such as imidazolium-2-carboxylate 1 are by far the most widely applied betaines for the generation of NHCs 2, because they decarboxylate thermally under relatively mild conditions.^6^ Pyrazolium-3-carboxylates,^7^ indazolium-3-carboxylates,^8^ and pyridinium-2-carboxylates^9^ react similarly to give the corresponding NHCs *in situ*, respectively. In general, the extrusion of heterocumulenes from pseudo-cross-conjugated mesomeric betaines is a valuable tool to generate NHCs. By contrast, cross-conjugated mesomeric betaines (CCMB) such as pyrazolium-4-carboxylates 3 require harsh reaction conditions for the formation of remote N-heterocyclic carbenes 4 so that this approach is not useful from a synthetic point of view.^10^ Some mesomeric betaines undergo reactions *via* their formal tautomers which are N-heterocyclic carbenes. The CMB 5 and the CCMB 7 are examples. Thus, nitron 5 reacts with sulfur to give a urea derivative as formal trapping product of the carbene 6.^11^ According to a computational study on relative stabilities of mesoionic and N-heterocyclic carbene tautomers in dependence on substituent effects, nitron 5 is by 2.3 kcal mol^−1^ more stable than its carbene 6.^12^ Similarly, betaine 7 tautomerizes to give carbene 8.^13^ Deprotonation of mesomeric betaines such as 9 results in the formation of anionic N-heterocyclic carbenes 10 (Scheme 1).^14^

**Scheme 2:**
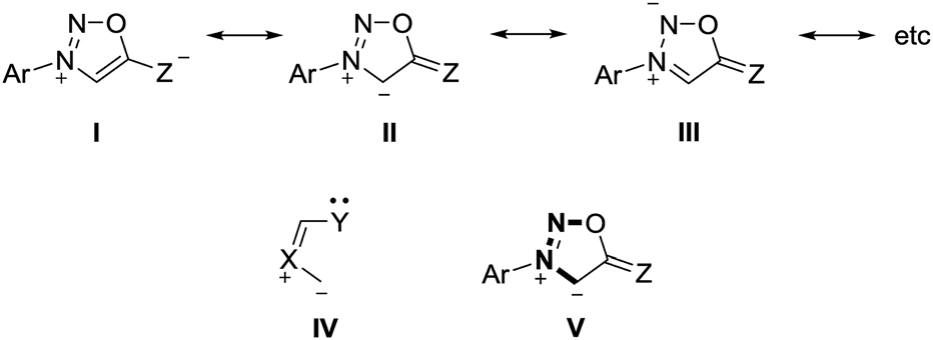
Features of sydnone derivatives.

Sydnones (Z = O) and their derivatives such as sydnone imines (Z = NR) and sydnone methides (Z = CR_2_) belong to the class of conjugated mesomeric betaines (CMB). Although the canonical structure I is the most common, the resonance structures II and III indicate that the exocyclic C–X bond corresponds to a carbonyl group for X = O which is in well agreement to results of X-ray analyses as well as vibrational spectroscopy.^15^ Sydnones and their derivatives possess the dipole type IV/V which is characteristic of the class of conjugated mesomeric betaines (CMB) (Scheme 2).^3^

**Scheme 3:**
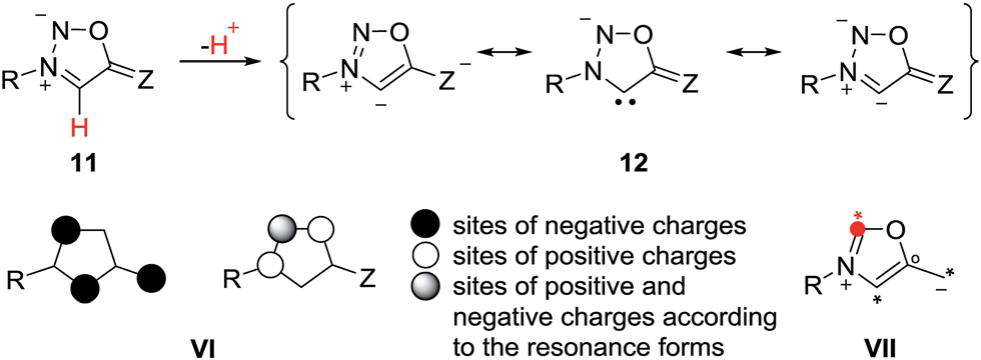
Characteristics of sydnone anions and of their derivatives.

The anions of sydnones and sydnone imines can be represented as anionic normal NHCs and abnormal NHCs. In the resonance forms, the delocalization of the negative charge of sydnone and sydnone imine anions include the site of deprotonation, *i.e.* C4 (VI), which is a starred (active) position according to a connectivity analysis (VII).^2^ Thus, anions of sydnone derivatives combine the features of N-heterocyclic carbenes due to their σ lone pair and of conjugated mesomeric betaines due to their π-architecture. Consequently, the highest occupied molecular orbitals (HOMO) are π-orbitals which display large atomic orbital coefficients on C4 (Scheme 3).

The sydnone imine anion 12 (Z = NR), usually stabilized by Li^+^, can be deuterated (13) and trapped by selenium, followed by methylation to give 14.^16^ It forms palladium as well as gold complexes such as 15 and 16.^16^ Iminium salts of formimidate are able to formylate the sydnone imine anions to yield 17,^17^ and the treatment with aldehydes give alcohols such as 18.^18^ Trapping with isocyanates give amides (19),^19^ and treatment with chlorodiphenylphosphane give phosphorus adducts like 20.^20^ Some cross-coupling reactions, catalyzed by Pd(PPh_3_)_4_ and copper salts, to yield 21 and 22 were also described.^18^ In addition to that, rearrangements of 12 have been reported.^19^

In continuation of our studies directed toward the chemistry of mesomeric betaines and their conversions into N-heterocyclic carbenes, we describe here trapping reactions of sydnone imine anions with sulfur, boron, and mercury.

## Results and discussion

**Scheme 4:**
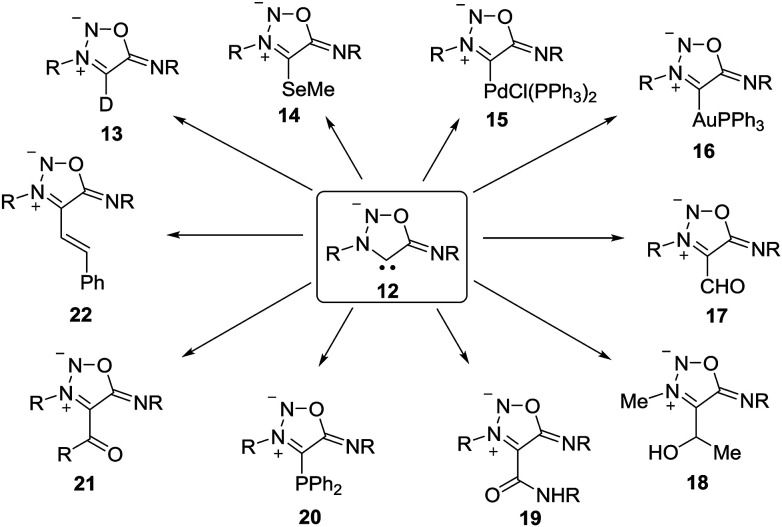
Reactions of sydnone imine anions.

**Scheme 5:**
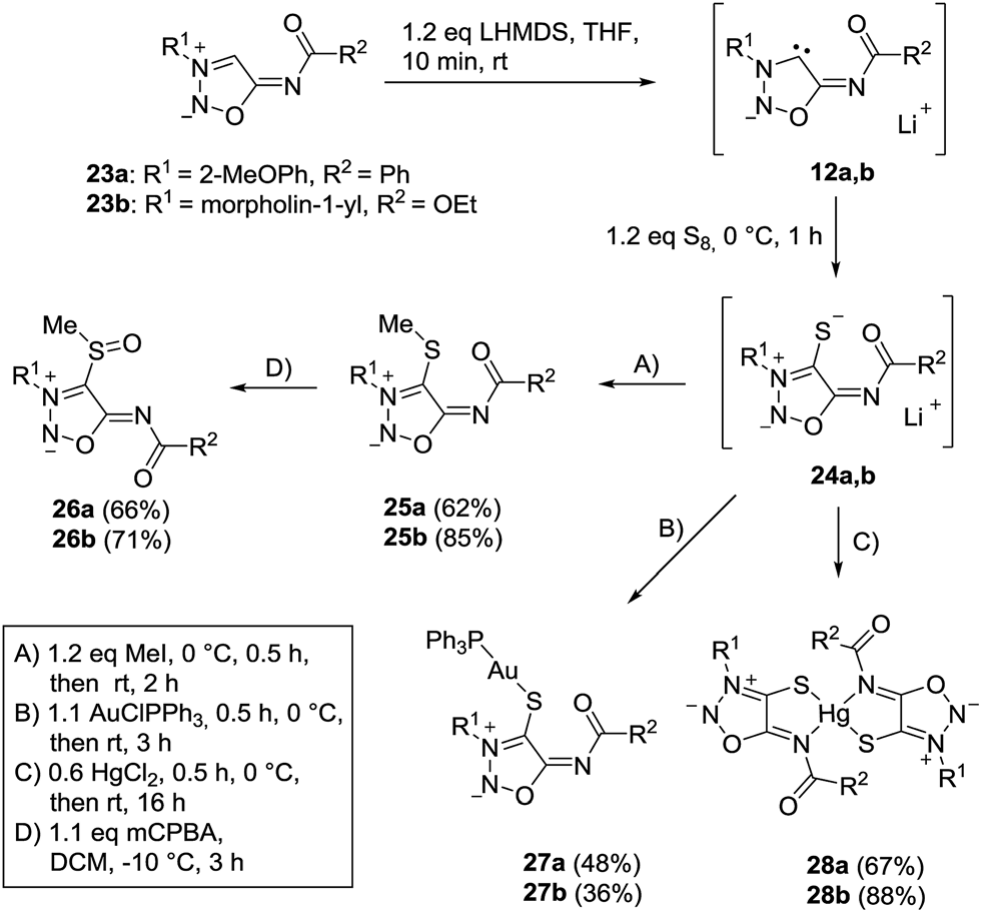
Trapping reactions of sydnone imines with sulfur and subsequent stabilizations.

The sydnone imine anions 12a,b, generated on treatment of the sydnone imines 23a and 23b (“molsidomine”) with lithium (trimethylsilyl)amide at rt, can be trapped by sulfur to give the corresponding sulfides 24a,b which could not be isolated (Scheme 5). Thiols of sydnone imines are very rare.^21^ Methylation by modified literature procedures yielded the stable^21,22^ sydnone imine thioethers 25a,b in acceptable yields. We were able to oxidize the thioethers 25a,b with *m*-chloroperoxybenzoic acid to give the sulfoxides 26a,b. Stabilization of the sulfides as gold complexes was accomplished on treatment of freshly prepared samples of the sulfides with chloro(triphenylphosphine)gold(i) which resulted in the formation of the complexes 27a,b. These complexes supplement the complexes 16 (Scheme 4) in which the gold is directly attached to C4 of the sydnone imines and which we described earlier.^16^ Although the complexes 27a,b are stable enough to survive purification by column chromatography, gold complexes with the transition metal directly bound to the C4 carbene carbon atom are far more stable. The sulfides were also stabilized as mercury complexes 28a,b which were formed on exposure of the sulfides with mercury(ii)chloride. Metal-stabilized sydnone sulfides are rare. To the best of our knowledge, only one tin complex has been described so far.^23^

**Fig. 1:**
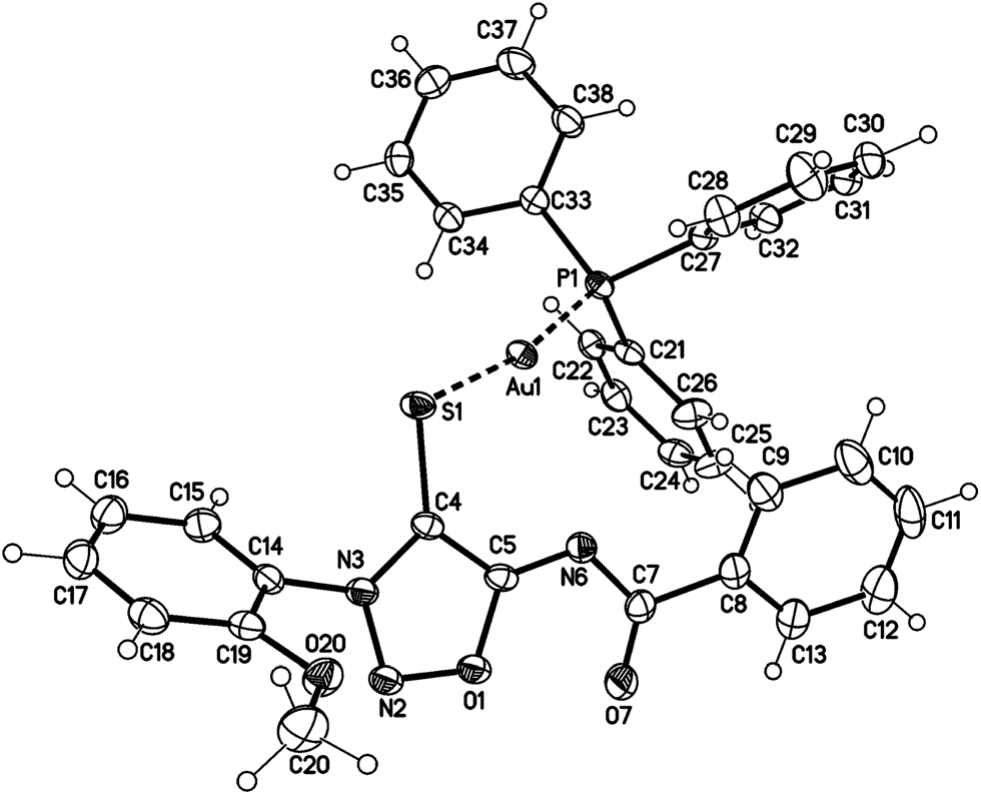
Molecular drawing of gold complex 27a (displacement parameters are drawn at 50% probability level). Selected bond lengths [pm] (crystallographic numbering): N2–N3: 132.4(2), N3–C4: 135.6(3), C4–C5: 140.9(3), C5–N6: 130.9(3), N6–C7: 136.8(3), C7–O7: 123.3(3), C4–S1: 172.3(2), S1–Au1: 232.76(5), N6–Au1: 298.97(17), Au1–P1: 225.91(5) pm.

Single crystals of the gold complex 27a were obtained by slow crystallization at −20 °C in a CHCl_3_–EtOAc mixture (Fig. 1). It is noteworthy to point out the angle P1–Au1–S1 is 168.824(19)° which is slightly bent towards the N6 nitrogen atom and not linear.

**Fig. 2:**
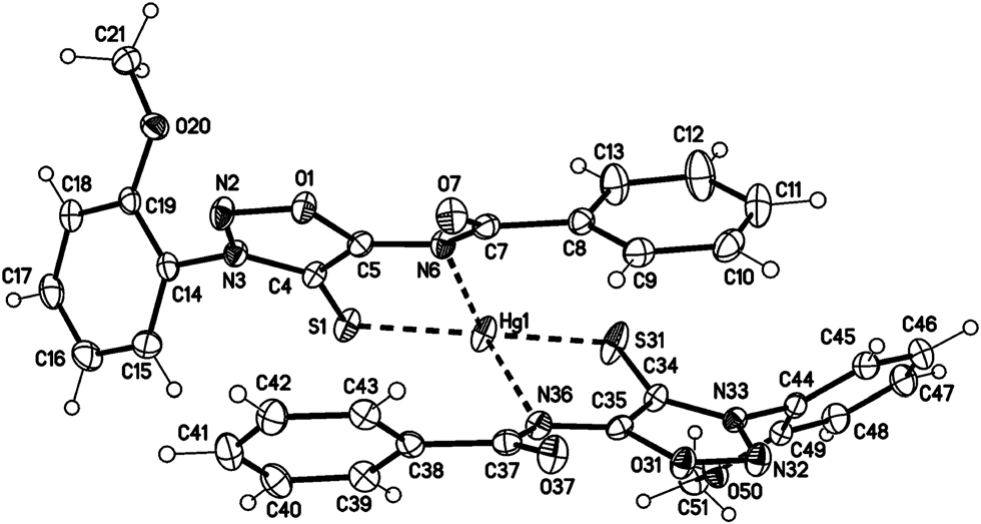
Molecular drawing of complex 28a (displacement parameters are drawn at 50% probability level). Selected bond lengths [pm] (crystallographic numbering): N2–N3: 132.5(2), N3–C4: 135.4(3), C4–C5: 140.4(3), C5–N6: 132.2(3), N6–C7: 138.5(2), C7–O7: 122.4(2), C4–S1: 171.6(2), S1–Hg1: 240.42(6), N6–Hg1: 247.27(17), N32–N33: 132.9(2), N33–C34: 134.8(2), C34–C35: 139.8(3), C35–N36: 132.4(3), N36–C37: 138.1(2), C37–O37: 122.8(2), C34–S31: 171.4(2), S31–Hg1: 239.19(6), N36–Hg1: 251.07(17) pm.

Single crystals of the mercury complex 28a were obtained by slow evaporation of a saturated solution in chloroform and diethyl ether. The complex crystallized in an orthorhombic space group. Crystal data show a bis(ligand) mercury(ii) complex with tetrahedral coordination of the mercury central atom with the N6 nitrogen atoms coordinating as neutral ligands (Fig. 2). Furthermore, the N6 coordination has a major influence on the C4–S1-metal angle. While the C4–S1–Au1 angle of complex 27a adopts a value of 101.65(7)°, the corresponding angle of the bis(ligand) mercury(ii) complex 28a has a value of 94.02(7)° (C34–S31–Au1 95.39(7)°).

**Scheme 6:**
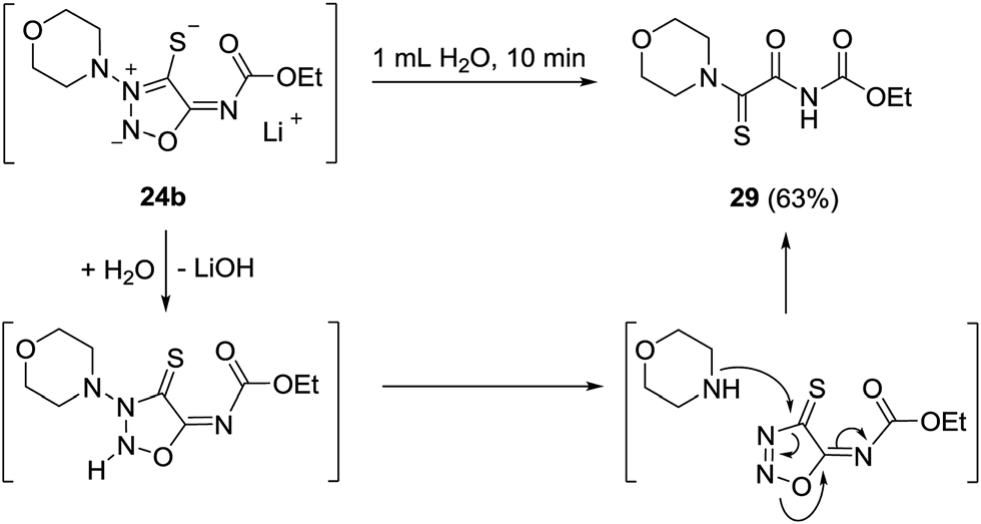
Morpholinyl group rearrangement starting from molsidomine (23b) *via* sulfide 24b.

The bond between the exocyclic substituent and the sydnone imine is known to be very stable under a variety of reaction conditions. However, the molsidomine derivative 24b surprisingly underwent an intramolecular rearrangement of the morpholinyl group and subsequent nitrogen cleavage for which the depicted mechanism is suggested (Scheme 6). Under analogous reaction conditions, 24a decomposed. Sydnone imine cleavages to form unsaturated nitriles such as N-morpholinoformimidoyl cyanide are known.^24^ They occur, however, when the exocyclic nitrogen atom is not substituted by electron-withdrawing groups. In these cases the N–N bond connecting the morpholine group and the sydnone imine remain intact.

**Fig. 3:**
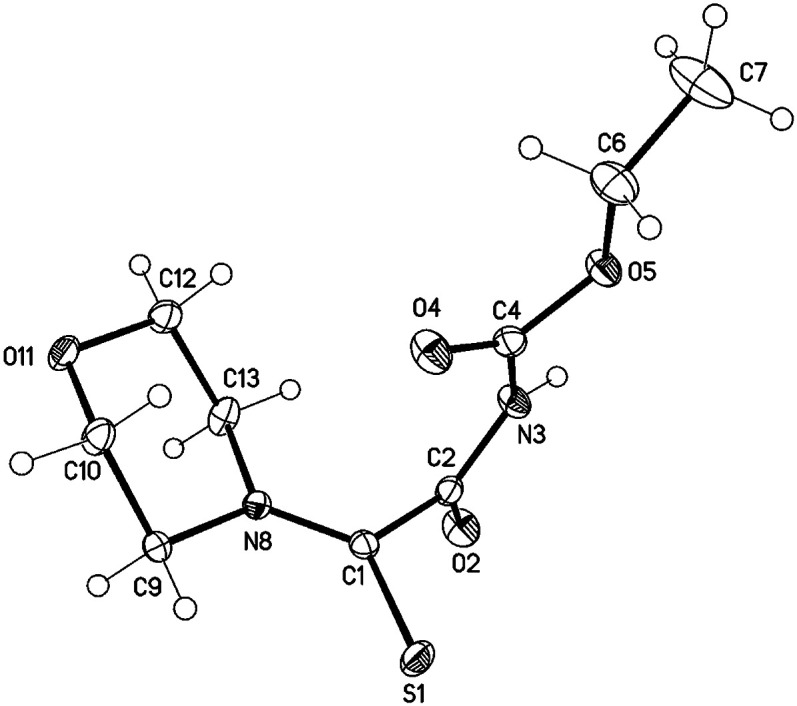
Molecular drawing of carbamate 29 (displacement parameters are drawn at 50% probability level). Selected bond lengths [pm] (crystallographic numbering): C1–N8: 131.77(16), C1–S1: 166.56(12), C1–C2: 151.61(16), C2–O2: 121.74(15), C2–N3: 137.07(16), N3–C4: 139.51(16), C4–O4: 120.04(16), S1–H9A: 264 pm.

We were able to obtain single crystals for an X-ray structure analysis by slow evaporation of 29 from a saturated solution in ethyl acetate. The structure was confirmed *via* single crystal X-ray analysis (Fig. 3).

**Fig. 4:**
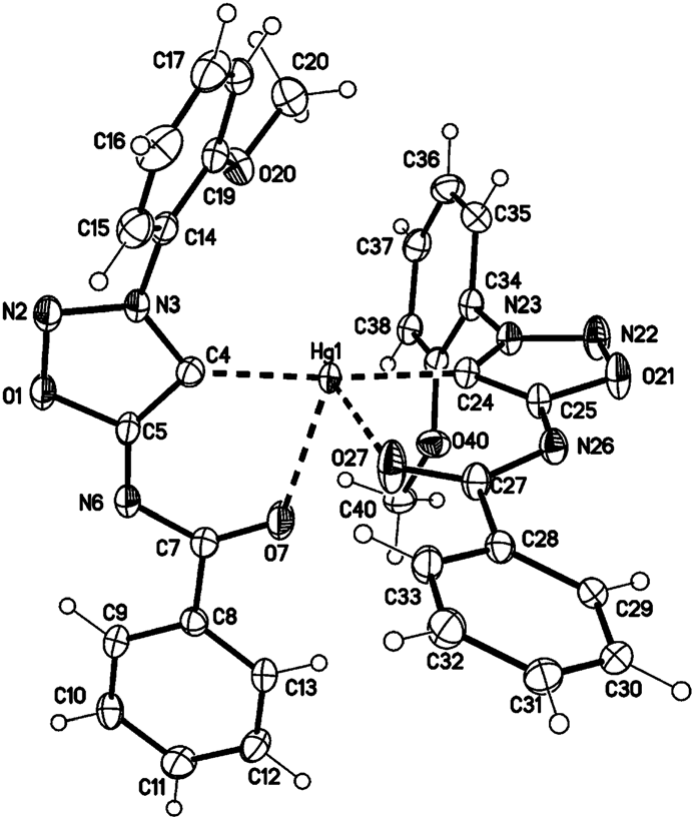
Molecular drawing of complex 30a (displacement parameters are drawn at 50% probability level). Selected bond lengths [pm] (crystallographic numbering): N2–N3: 131.3(4), N3–C4: 135.6(4), C4–C5: 139.9(5), C5–N6: 131.5(4), N6–C7: 135.5(4), C7–O7: 124.5(4), C4–Hg1: 205.0(3), Hg1–O7: 255.1(3), N22–N23: 131.7(4), N23–C24: 135.1(4), C24–C25: 140.2(4), C25–N26: 132.0(4), N26–C27: 135.7(4), C27–O27: 123.8(4), C24–Hg1: 204.6(3), Hg1–O27: 255.2(3) pm.

In comparison to the sydnone imine sulfide mercury(ii) complexes we furthermore prepared the transition metal complexes with mercury directly attached to the C4 carbene carbon atom in high yields. These complexes show very high stability towards water, air, bases and higher temperatures. Crystal data show a tetrahedral coordination of the mercury(ii) atom (Fig. 4). In comparison to the complex 28a where coordination is observed by the S1 and N6 atom (forming a five-membered ring), the complex 30a shows coordination by the C4 and O7 atom (forming a six-membered ring).

**Scheme 7:**
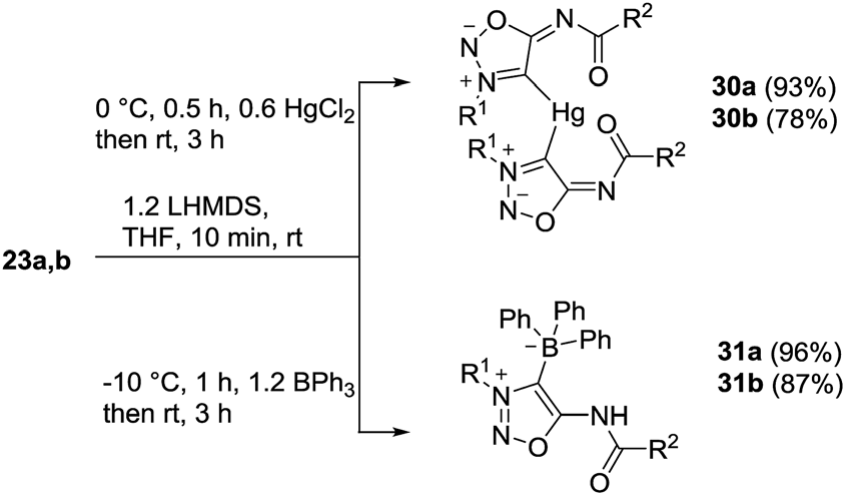
Reaction of sydnone imines 23a,b with mercury(ii) and triphenylborane.

Moreover, we managed to undergo covalent bond formation with triphenyl borane, resulting in a new mesomeric betaine structure in which the negative charge is in the borate substituent (Scheme 7).

**Fig. 5:**
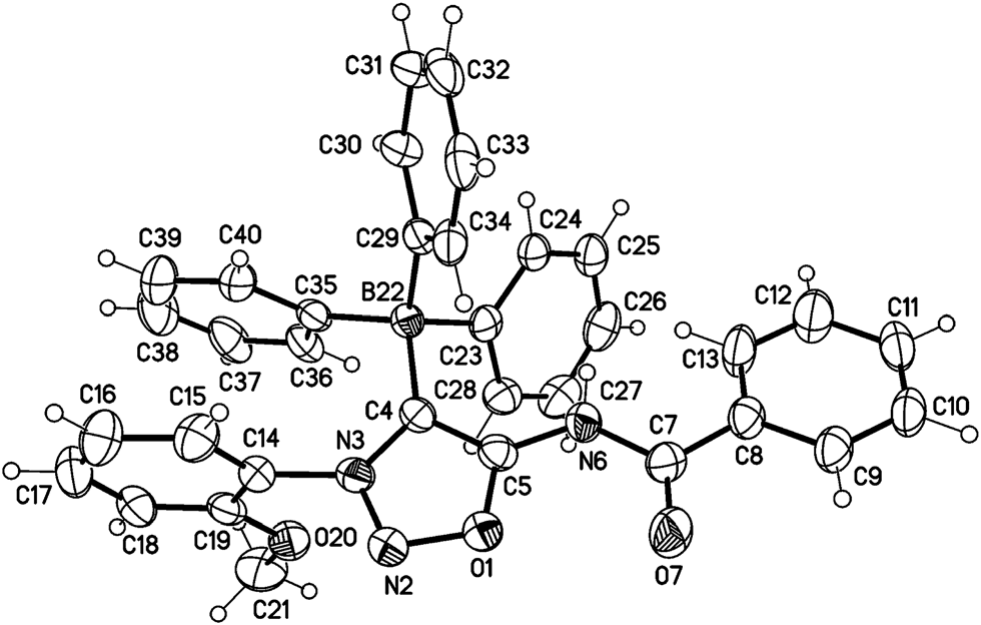
Molecular drawing of boron compound 31a (one of the two crystallographic independent molecules is shown, displacement parameters are drawn at 50% probability level). Selected bond lengths [pm] (crystallographic numbering, for the second molecule in brackets): O1–N2: 136.6(2) [135.8(2)], N2–N3: 131.5(2) [130.7(2)], N3–C4: 136.8(3) [136.9(3)], C4–C5: 137.9(3) [138.3(3)], O1–C5: 134.7(3) [134.7(2)], C5–N6: 136.1(3) [136.3(3)], N6–C7: 137.7(3) [139.6(3)], C7–O7: 121.6(3) [121.7(3)], C4–B22: 165.2(3) [165.6(3)] pm.

The structure was confirmed by a single crystal X-ray analysis (Fig. 5). The molecular drawing shows a tetrahedral arrangement of the covalently bound boron atom. The C4–B22 bond length (165.2(3) pm) (C104–B122 165.6(3) pm) is in agreement with the Ph_4_B^−^ bond length (164.3 pm) presented in the literature.^25^

## Experimental

The reactions were carried out under an atmosphere of nitrogen in oven-dried glassware. Nuclear magnetic resonance (NMR) spectra were obtained with a Bruker Avance 400 and Bruker Avance III 600 MHz. ^1^H NMR spectra were measured at 400 MHz or 600 MHz and ^13^C NMR spectra were measured at 100 MHz or 150 MHz, with the solvent peak or tetramethylsilane used as the internal reference. Multiplicities are described by using the following abbreviations: s = singlet, d = doublet, t = triplet, q = quadruplet, and m = multiplet, and the signal orientations in DEPT experiments were described as follows: o = no signal; + = up (CH, CH_3_); − = down (CH_2_). ATR-IR spectra were obtained on a Bruker Alpha in the range of 400 to 4000 cm^−1^. Melting points were measured by differential scanning calorimetry (DSC) using a DSC 6 apparatus (Perkin-Elmer). The HR-MS spectra were measured on a Bruker Daltonik Tesla-Fourier transform-ion cyclotron resonance mass spectrometer with electrospray ionisation. Yields are not optimized.

### Crystal structure determinations

The single-crystal X-ray diffraction study were carried out on a Bruker D8 Venture diffractometer with Photon100 (29) or PhotonII detector (27a, 28a, 30a, 31a) at 123(2) K using Cu-Kα radiation (*λ* = 1.54178 Å) (29, 31a) or Mo-Kα radiation (*λ* = 0.71073 Å) (27a, 28a, 30a). Dual space/intrinsic methods (27a, 28a, 30a, 31a) (SHELXT)^26^ or direct methods (29) (SHELXS-97)^27^ were used for structure solution and refinement was carried out using SHELXL-2014 (full-matrix least-squares on *F*^2^).^26^ Hydrogen atoms were localized by difference electron density determination and refined using a riding model (in 29 H(N) free). Semi-empirical absorption corrections and extinction correction (for 28a, 29) were applied. In 31a refinement with the listed atoms show in one void residual electron density due to a heavily disordered diethylether which could not be refined with split atoms. Therefore the option “SQUEEZE” of the program package PLATON^28^ was used to create a hkl file taking into account the residual electron density in the void areas. Therefore the atoms list and unit card do not agree (see cif-.file for details).

27a: yellow plates, C_34_H_27_N_3_O_3_SPAu, *M* = 785.58, crystal size 0.10 × 0.05 × 0.02 mm, triclinic, space group *P*1̄ (no. 2), *a* = 11.3805 (5) Å, *b* = 11.7754 (5) Å, *c* = 13.5775 (5) Å, *α* = 90.886(1)°, *β* = 114.586(1)°, *γ* = 112.289(1)°, *V* = 1498.12 (11) Å^3^, *Z* = 2, *ρ*(calc) = 1.742 Mg m^−3^, *F*(000) = 772, *μ*(MoKα) = 5.07 mm^−1^, 43 043 reflections (2*θ*_max_ = 55.0°), 6890 unique [*R*_int_ = 0.034], 389 parameters, *R*_1_ (for 6512 *I* > 2*σ*(*I*)) = 0.016, *wR*2 (all data) = 0.036, *S* = 1.07, largest diff. peak and hole 0.75 and −0.47 eÅ^−3^.

28a: yellow blocks, C_32_H_24_N_6_O_6_S_2_Hg, *M* = 853.28, crystal size 0.16 × 0.10 × 0.04 mm, triclinic, space group *P*1̄ (no. 2), *a* = 8.5954(11) Å, *b* = 11.3147(14) Å, *c* = 17.4308(19) Å, *α* = 77.613(4)°, *β* = 86.366(4)°, *γ* = 70.249(4)°, *V* = 1558.3(3) Å^3^, *Z* = 2, *ρ*(calc) = 1.819 Mg m^−3^, *F*(000) = 836, *μ*(MoKα) = 5.13 mm^−1^, 38 298 reflections (2*θ*_max_ = 55.0°), 7157 unique [*R*_int_ = 0.031], 427 parameters, *R*_1_ (for 6733 *I* > 2*σ*(*I*)) = 0.018, *wR*2 (all data) = 0.037, *S* = 1.08, largest diff. peak and hole 0.71 and −0.67 eÅ^−3^.

29: colourless blocks, C_9_H_14_N_2_O_4_S, *M* = 246.28, crystal size 0.45 × 0.35 × 0.15 mm, monoclinic, space group *C*2/*c* (no. 15), *a* = 23.9648(12) Å, *b* = 6.5378(3) Å, *c* = 14.6598(8) Å, *β* = 92.902(1)°, *V* = 2293.9(2) Å^3^, *Z* = 8, *ρ*(calc) = 1.426 Mg m^−3^, *F*(000) = 1040, *μ*(CuKα) = 2.56 mm^−1^, 14 352 reflections (2*θ*_max_ = 144.4°), 2240 unique [*R*_int_ = 0.023], 149 parameters, 1 restraint, *R*_1_ (for 2230 *I* > 2*σ*(*I*)) = 0.029, *wR*2 (all data) = 0.073, *S* = 1.09, largest diff. peak and hole 0.36 and −0.27 eÅ^−3^.

30a: colourless blocks, C_32_H_24_N_6_O_6_Hg, *M* = 789.16, crystal size 0.04 × 0.02 × 0.01 mm, triclinic, space group *P*1̄ (no. 2), *a* = 10.4627(5) Å, *b* = 11.4660(6) Å, *c* = 13.4682(7) Å, *α* = 79.335(2)°, *β* = 68.151(2)°, *γ* = 89.637(2)°, *V* = 1470.28(13) Å^3^, *Z* = 2, *ρ*(calc) = 1.783 Mg m^−3^, *F*(000) = 772, *μ*(MoKα) = 5.29 mm^−1^, 55 028 reflections (2*θ*_max_ = 55.0°), 6727 unique [*R*_int_ = 0.044], 408 parameters, *R*_1_ (for 6372 *I* > 2*σ*(*I*)) = 0.024, *wR*2 (all data) = 0.050, *S* = 1.19, largest diff. peak and hole 1.66 and −2.23 eÅ^−3^.

31a: colourless plates, C_34_H_28_BN_3_O_3_·0.25(C_4_H_10_O), *M* = 555.93, crystal size 0.20 × 0.12 × 0.06 mm, triclinic, space group *P*1̄ (no. 2), *a* = 11.2078 (4) Å, *b* = 16.0505 (6) Å, *c* = 16.8226 (7) Å, *α* = 74.625(2)°, *β* = 88.792(2)°, *γ* = 85.859(2)°, 2910.3 (2) Å^3^, *Z* = 4, *ρ*(calc) = 1.269 Mg m^−3^, *F*(000) = 1170, *μ*(CuKα) = 0.65 mm^−1^, 38 234 reflections (2*θ*_max_ = 144.4°), 11 373 unique [*R*_int_ = 0.038], 741 parameters, *R*_1_ (for 9893 *I* > 2*σ*(*I*)) = 0.060, *wR*2 (all data) = 0.172, *S* = 1.04, largest diff. peak and hole 0.46 and −0.28 eÅ^−3^.

### Synthesis

#### 6-Benzoyl-3-(2-methoxyphenyl)-4-methylthio-sydnone imine (25a)

0.30 g (1.0 mmol) of 6-benzoyl-3-(2-methoxyphenyl)-sydnone imine are dissolved in 12 mL THF (abs.) under an inert atmosphere. Then 1.20 mL (1.2 mmol) of LHMDS (1 M in THF) are added at rt. The mixture is cooled to 0 °C treated with 0.04 g (1.2 mmol) of sulfur and stirred for 1 h. Then 0.08 mL (1.2 mmol) of MeI are added. After stirring for 30 minutes at 0 °C the flask is warmed up to rt and stirred for another 2 h. The crude product is purified by column chromatography (DCM : MeOH 40 : 1). Yield: 0.21 g (62%) of a yellow solid, mp 248 °C (decomp.). ^1^H-NMR (400 MHz, CDCl_3_): *δ* = 8.33–8.35 (m, 2H, 9/9′-*H*), 7.65–7.69 (m, 1H, 15-H), 7.47–7.51 (m, 1H, 11-H), 7.41–7.46 (m, 3H, 10/10′-H + 17-H), 7.21–7.16 (m, 2H, 16-H + 14-H), 3.90 (s, 3H, 19-H), 2.47 (s, 3H, 21-H) ppm; ^13^C-NMR (100 MHz, CDCl_3_): *δ* = 172.8 (o, C-7), 167.2 (o, C-5), 153.5 (o, C-13), 137.3 (o, C-8), 134.1 (+, C-15), 131.4 (+, C-11), 129.6 (+, C-9/9′), 127.9 (+, C-10/10′), 127.1 (+, C-17), 121.7 (o, C-12), 121.1 (+, C-16), 114.2 (o, C-4), 112.6 (+, C-14), 56.1 (+, C-19), 7.4 (+, C-21) ppm; IR (ATR): 3057, 2928, 2842, 1558, 1499, 1353, 1286, 1218, 1159, 1087, 1017, 794, 754, 706, 529 cm^−1^; HR-ESI-MS: calcd for C_17_H_16_N_3_O_3_S^+^ 342.0912. Found 342.0915.

#### 6-Ethoxycarbonyl-4-methylthio-3-morpholinyl-sydnone imine (25b)

0.50 g (2.1 mmol) 6-ethoxycarbonyl-3-morpholinyl-sydnone imine are dissolved in 18 mL THF (abs.) under an inert atmosphere. Then 2.27 mL (2.3 mmol) of LHMDS (1 M in THF) are added at rt. The mixture is cooled to 0 °C, treated with 0.08 g (2.5 mmol) of sulfur and stirred for 1 h. Then 0.16 mL (2.5 mmol) of MeI are added. After stirring for 30 minutes at 0 °C the flask is warmed up to rt and stirred for another 2 h. The crude product is purified by column chromatography (EE). Yield: 0.51 g (85%) of a white solid, mp 137 °C, 224 °C (decomp.). ^1^H-NMR (400 MHz, CDCl_3_): *δ* = 4.20 (q, 2H, ^3^*J*_H,H_ = 7.1 Hz, 9-H), 3.96–3.99 (m, 4H, 13/13′-H), 3.51–3.54 (m, 4H, 12/12′-H), 2.46 (s, 3H, 16-H), 1.32 (t, 3H, ^3^*J*_H,H_ = 7.1 Hz, 10-H) ppm; ^13^C-NMR (100 MHz, CDCl_3_): *δ* = 167.2 (o, C-5), 159.3 (o, C-7), 105.9 (o, C-4), 65.9 (−, C-13/13′), 61.3 (−, C-9), 55.2 (−, C-12/12′), 16.6 (+, C-16), 14.4 (+, C-10) ppm; IR (ATR): 2973, 2931, 2901, 2858, 1660, 1595, 1408, 1368, 1290, 1260, 1204, 1102, 1049, 977, 878, 790, 722, 655, 557, 504 cm^−1^; HR-ESI-MS: calcd for C_10_H_17_N_4_O_4_S^+^ 289.0971. Found 289.0970.

#### 6-Benzoyl-3-(2-methoxyphenyl)-4-methylsulfinyl-sydnone imine (26a)

0.27 g (0.8 mmol) 6-benzoyl-3-(2-methoxyphenyl)-4-methylthio-sydnone imine are dissolved in 15 mL DCM at −10 °C, then 0.21 g (0.9 mmol) of *m*-chloroperbenzoic acid (77 w%) are added. The mixture is stirred for 3 h at −10 °C and treated with a saturated NaHCO_3_ solution in water. The organic layer is separated. Then the water layer is extracted 3× with DCM and dried over MgSO_4_. The crude product is purified by column chromatography (EE : PE 1 : 1). Yield: 0.19 g (66%) of a white solid, mp 158 °C, 185 °C (decomp.). ^1^H-NMR (400 MHz, CDCl_3_): *δ* = 8.28–8.31 (m, 2H, 9/9′-H), 7.67–7.71 (m, 1H, 15-H), 7.49–7.57 (m, 2H, 11/17-H), 7.42–7.46 (m, 2H, 10/10′-H), 7.21–7.16 (m, 2H, 16/14-H), 3.94 (s, 3H, 19-H), 3.37 (s, 3H, 21-H) ppm; ^13^C-NMR (100 MHz, CDCl_3_): *δ* = 173.0 (o, C-7), 163.8 (o, C-5), 153.0 (o, C-13), 136.3 (o, C-8), 134.7 (+, C-15), 132.0 (+, C-11), 129.8 (+, C-9/9′), 128.0 (+, C-10/10′), 127.1 (+, C-17), 121.4 (+, C-16), 121.0 (o, C-12), 117.3 (o, C-4), 112.6 (+, C-14), 56.3 (+, C-19), 37.6 (+, C-21) ppm; IR (ATR): 3062, 3023, 2948, 1642, 1573, 1498, 1467, 1354, 1313, 1285, 1275, 1234, 1159, 1060, 1034, 1017, 982, 939, 807, 753, 706, 681, 586, 530, 474, 422 cm^−1^; HR-ESI-MS: calcd for C_17_H_15_N_3_O_4_SNa^+^ 380.0681. Found 380.0680.

#### 6-Ethoxycarbonyl-4-methylsulfinyl-3-morpholinyl-sydnone imine (26b)

0.11 g (0.4 mmol) of 6-ethoxycarbonyl-4-methylthio-3-morpholinyl-sydnone imine are dissolved in 10 mL DCM at −10 °C, then 0.07 g (0.4 mmol) of *m*-chloroperbenzoic acid (77 w%) are added. The mixture is stirred for 3 h at −10 °C and treated with a saturated NaHCO_3_ solution in water. The organic layer is separated. Then the water layer is extracted 3× with DCM and dried over MgSO_4_. The crude product is purified by column chromatography (EE : MeOH 20 : 1). Yield: 0.08 g (71%) of a white solid, mp 158 °C (decomp.). ^1^H-NMR (400 MHz, CDCl_3_): *δ* = 4.15–4.27 (m, 2H, 9-H), 3.97–4.00 (m, 4H, 13/13′-H), 3.56–3.65 (m, 4H, 12/12′-H), 3.30 (s, 3H, 16-H), 1.33 (t, 3H, ^3^*J*_H,H_ = 7.1 Hz, 10-H) ppm; ^13^C-NMR (100 MHz, CDCl_3_): *δ* = 162.8 (o, C-5), 158.8 (o, C-7), 111.5 (o, C-4), 66.1 (−, C-13/13′), 62.0 (−, C-9), 56.7 (−, C-12/12′), 36.3 (+, C-16), 14.4 (+, C-10) ppm; IR (ATR): 2986, 2914, 2865, 1670, 1607, 1406, 1367, 1299, 1255, 1206, 1147, 1103, 1042, 980, 942, 881, 798, 759, 719, 638, 560, 485, 443 cm^−1^; HR-ESI-MS: calcd for C_10_H_16_N_4_O_5_SNa^+^ 289.0739. Found 327.0738.

#### ((6-Benzoyl-3-(2-methoxyphenyl)-sydnone imine-4-yl)thio)-(triphenylphosphine)-gold(i) (27a)

0.075 g (0.25 mmol) of 6-benzoyl-3-(2-methoxyphenyl)-sydnone imine are dissolved in 8 mL of THF (abs.) under an inert atmosphere. Then 0.30 mL (0.30 mmol) of LHMDS (1 M in THF) are added at rt. The mixture is cooled to 0 °C, treated with 0.001 g (0.30 mmol) of sulfur and stirred for 2.5 h. Then 0.150 g (0.30 mmol) of AuCl(PPh_3_) are added. After stirring for 30 minutes at 0 °C the flask is warmed up to rt and stirred for another 3 h. The crude product is purified by column chromatography (PE : EE 1 : 2). Yield: 0.096 g (48%) of a yellow solid, mp 199 °C (decomp.). ^1^H-NMR (600 MHz, CDCl_3_): *δ* = 8.24–8.26 (m, 2H, 9/9′-H), 7.57–7.60 (m, 1H, 15-H), 7.37–7.44 (m, 10H, 24/24′-H + 26-H + 17-H), 7.31–7.33 (m, 6H, 25/25′-H), 7.11–7.14 (m, 2H, 14-H + 16-H), 7.05–7.08 (m, 1H, 11-H), 6.78–6.81 (m, 2H, 10/10′-H), 3.88 (s, 3H, 19-H) ppm; ^13^C-NMR (150 MHz, CDCl_3_): *δ* = 172.9 (o, C-7), 168.6 (o, C-5), 154.1 (o, C-13), 137.9 (o, C-8), 134.1 (+, d, ^2^*J*_C,P_ = 14.0 Hz, C-24/24′), 133.3 (+, C-15), 131.5 (+, d, ^4^*J*_C,P_ = 1.7 Hz, C-26), 130.3 (+, C-11), 129.4 (+, C-9/9′), 129.2 (o, d, ^1^*J*_C,P_ = 55.5 Hz, C-23), 129.0 (+,d, ^3^*J*_C,P_ = 11.8 Hz, C-25/25′), 127.8 (+, C-17), 127.2 (+, C-10/10′), 125.7 (o, C-4), 122.6 (o, C-12), 120.8 (+, C-16), 112.8 (+, C-14), 56.1 (+, C-19) ppm; ^31^P-NMR: (243 MHz, CDCl_3_): *δ* = 36.6 ppm; IR (ATR): 3052, 2966, 2863, 1608, 1555, 1503, 1435, 1359, 1292, 1253, 1159, 1099, 1043, 1019, 996, 906, 744, 692, 539, 496 cm^−1^; HR-ESI-MS: calcd for C_34_H_27_N_3_O_3_SAuPNa^+^ 808.1074. Found 808.1070.

#### ((6-Ethoxycarbonyl-3-morpholinyl-sydnone imine-4-yl)thio)-(triphenylphosphine)-gold(i) (27b)

0.061 g (0.25 mmol) of 6-ethoxycarbonyl-3-morpholinyl sydnone imine are dissolved in 8 mL of THF (abs.) under an inert atmosphere. Then 0.30 mL (0.30 mmol) of LHMDS (1 M in THF) are added at rt. The mixture is cooled to 0 °C, treated with 0.001 g (0.30 mmol) of sulfur and stirred for 2.5 h. Then 0.150 g (0.30 mmol) of AuCl(PPh_3_) are added. After stirring for 30 minutes at 0 °C the flask is warmed up to rt and stirred for another 3 h. The crude product is purified by column chromatography (PE : EE 1 : 2). Yield: 0.067 g (36%) of a yellow solid, mp 155 °C (decomp.). ^1^H-NMR (600 MHz, CDCl_3_): *δ* = 7.60 (m, 6H, 19/19′-H), 7.48–7.50 (m, 3H, 21-H), 7.42–7.45 (m, 6H, 20/20′-H), 3.93–3.94 (m, 4H, 13/13′-H), 3.73 (q, 2H, ^3^*J*_H,H_ = 7.1 Hz, 9-H), 3.51–3.53 (m, 4H, 12/12′-H), 0.76 (t, 3H, ^3^*J*_H,H_ = 7.1 Hz, 10-H) ppm; ^13^C-NMR (150 MHz, CDCl_3_): *δ* = 169.1 (o, C-5), 159.8 (o, C-7), 134.3 (+, d, ^2^*J*_C,P_ = 14.1 Hz, C-19/19′), 131.4 (+, C-21), 130.0 (o, d, ^1^*J*_C,P_ = 57.5 Hz, C-18), 128.9 (+,d, ^3^*J*_C,P_ = 11.7 Hz, C-20/20′), 119.4 (o, C-4), 66.1 (−, C-13/13′), 60.4 (−, C-9), 53.5 (−, C-12/12′), 14.1 (+, C-10) ppm; ^31^P-NMR: (243 MHz, CDCl_3_): *δ* = 36.6 ppm; IR (ATR): 2972, 2859, 1659, 1593, 1239, 1180, 1100, 1070, 879, 746, 692, 537, 508 cm^−1^; HR-ESI-MS: calcd for C_27_H_28_N_4_O_4_SAuPNa^+^ 755.1132. Found 755.1124.

#### (Bis(6-benzoyl-3-(2-methoxyphenyl)-sydnone imine-4-yl)thio)mercury(ii) (28a)

0.15 g (0.5 mmol) of 6-benzoyl-3-(2-methoxyphenyl)-sydnone imine are dissolved in 10 mL of THF (abs.) under an inert atmosphere. Then 0.60 mL (0.6 mmol) of LHMDS (1 M in THF) are added at rt. The mixture is cooled to 0 °C, treated with 0.02 g (0.6 mmol) of sulfur and stirred for 2.5 h. Then 0.08 g (0.3 mmol) of HgCl_2_ are added. After stirring for 30 minutes at 0 °C the flask is warmed up to rt and stirred for another 3 h. The crude product is purified by column chromatography (PE : EE : DCM 2 : 4 : 1). Yield: 0.14 g (67%) of a yellow solid, mp 189 °C (decomp.). ^1^H-NMR (600 MHz, CDCl_3_): *δ* = 8.05–8.07 (m, 2H, 9/9′-H), 7.62–7.65 (m, 1H, 15-H), 7.38–7.40 (m, 1H, 11-H), 7.24–7.27 (m, 3H, 10/10′-H + 17-H), 7.15–7.18 (m, 1H, 16-H), 7.13–7.15 (m, 1H, 14-H), 3.82 (s, 3H, 19-H) ppm; ^13^C-NMR (150 MHz, CDCl_3_): *δ* = 173.4 (o, C-7), 168.9 (o, C-5), 153.6 (o, C-13), 137.1 (o, C-8), 134.0 (+, C-15), 130.8 (+, C-11), 129.2 (+, C-9/9′), 127.6 (+, C-10/10′), 127.2 (+, C-17), 126.0 (o, C-4), 121.5 (o, C-12), 121.0 (+, C-16), 113.0 (+, C-14), 56.1 (+, C-19) ppm; IR (ATR): 3057, 2841, 1629, 1546, 1500, 1366, 1287, 1198, 1162, 1121, 1055, 1014, 912, 760, 718, 696, 537 cm^−1^; HR-ESI-MS: calcd for C_32_H_24_N_6_O_6_S_2_HgNa^+^ 877.0797. Found 877.0764.

#### Bis((6-ethoxycarbonyl-3-morpholinyl-sydnone imine-4-yl)thio)mercury(ii) (28b)

0.12 g (0.5 mmol) of 6-ethoxycarbonyl-3-morpholinyl-sydnone imine are dissolved in 10 mL of THF (abs.) under an inert atmosphere. Then 0.60 mL (0.6 mmol) of LHMDS (1 M in THF) are added at rt. The mixture is cooled to 0 °C treated with 0.02 g (0.6 mmol) of sulfur and stirred for 2.5 h. Then 0.08 g (0.3 mmol) of HgCl_2_ are added. After stirring for 30 minutes at 0 °C the flask is warmed up to rt and stirred for another 3 h. Formation of a yellow precipitate is observed. The solid is filtered off and washed with THF and Et_2_O. Yield: 0.16 g (88%) of a yellow solid, mp 179 °C (decomp.). ^1^H-NMR (600 MHz, CDCl_3_): *δ* = 4.01 (q, 2H, ^3^*J*_H,H_ = 7.1 Hz, 9-H), 3.99–4.00 (m, 4H, 13/13′-H), 3.56–3.58 (m, 4H, 12/12′-H), 1.01 (t, 3H, ^3^*J*_H,H_ = 7.1 Hz, 10-H) ppm; ^13^C-NMR (150 MHz, CDCl_3_): *δ* = 168.4 (o, C-5), 157.4 (o, C-7), 119.2 (o, C-4), 66.0 (−, C-13/13′), 61.3 (−, C-9), 53.7 (−, C-12/12′), 14.1 (+, C-10) ppm; IR (ATR): 2977, 2924, 2900, 2871, 1671, 1596, 1432, 1365, 1289, 1248, 1191, 1089, 1021, 983, 879, 772, 724, 610, 593, 561, 511 cm^−1^; HR-ESI-MS: calcd for C_18_H_26_N_8_O_8_S_2_HgNa^+^ 771.0919. Found 771.0916.

#### Ethyl-*N*-(1-morpholinyl-1-thioxo-ethan-2-one)-carbamate (29)

0.24 g (1.0 mmol) of 6-ethoxycarbonyl-3-morpholinyl-sydnone imine are dissolved in 10 mL of THF (abs.) under an inert atmosphere. Then 1.20 mL (1.2 mmol) of LHMDS (1 M in THF) are added at rt. The mixture is cooled to 0 °C, treated with 0.04 g (1.2 mmol) of sulfur and stirred for 2 h. Then 1 mL of water is added. After stirring for 30 minutes the solvent is removed under removed pressure. The crude product is purified by column chromatography (EE). Yield: 0.15 g (63%) of a yellow solid, mp 134 °C. ^1^H-NMR (400 MHz, CDCl_3_): *δ* = 8.43 (br. s, 1H, 3-H), 4.26 (q, 2H, ^3^*J*_H,H_ = 7.1 Hz, 6-H), 4.18–4.20 (m, 2H, 9-H/9′-H), 3.85–3.88 (m, 2H, 10-H/10′-H), 3.78–3.81 (m, 2H, 10-H/10′-H), 3.71–3.74 (m, 2H, 9-H/9′-H), 1.32 (t, 3H, ^3^*J*_H,H_ = 7.1 Hz, 7-H) ppm; ^13^C-NMR (100 MHz, CDCl_3_): *δ* = 189.3 (o, C-1), 163.6 (o, C-2), 150.7 (o, C-4), 65.9 (−, C-10/C-10′), 65.8 (−, C-10/C-10′), 63.0 (−, C-6), 52.3 (−, C-9/C-9′), 47.3 (−, C-9/C-9′), 14.1 (+, C-7) ppm; IR (ATR): 3126, 2938, 2873, 1760, 1688, 1515, 1432, 1339, 1275, 1209, 1098, 1066, 1040, 1017, 874, 813, 763, 703, 609, 520 cm^−1^; HR-ESI-MS: calcd for C_9_H_15_N_2_O_4_SNa^+^ 289.0572. Found 269.0570.

#### Bis(6-benzoyl-3-(2-methoxyphenyl)-sydnone imine-4-yl)-mercury(ii) (30a)

0.15 g (0.50 mmol) of 6-benzoyl-3-(2-methoxyphenyl)-sydnone imine are dissolved in 10 mL of THF (abs.) under an inert atmosphere. Then 0.60 mL (0.60 mmol) of LHMDS (1 M in THF) are added at rt. The mixture is cooled to 0 °C and treated with 0.08 g (0.30 mmol) of HgCl_2_. After stirring for 0.5 h at 0 °C the flask is warmed up to rt and stirred for another 3 h. The crude product is purified by column chromatography (EE : PE : DCM 4 : 2 : 1). Yield: 0.18 g (93%) of a white solid, mp 244 °C (decomp.). ^1^H-NMR (600 MHz, CDCl_3_): *δ* = 8.10–8.12 (m, 2H, 9/9′-H), 7.78–7.79 (m, 1H, 17-H), 7.44–7.46 (m, 2H, 11-H + 15-H), 7.36–7.39 (m, 2H, 10/10′-H), 6.99–7.03 (m, 2H, 16-H + 14-H), 3.75 (s, 3H, 19-H) ppm; ^13^C-NMR (150 MHz, CDCl_3_): *δ* = 178.2 (o, C-5), 172.7 (o, C-7), 152.8 (o, C-13), 143.4 (o, C-4), 138.1 (o, C-8), 133.5 (+, C-15), 131.0 (+, C-11), 129.3 (+, C-9/9′), 127.7 (+, C-10/10′), 127.6 (+, C-17), 125.2 (o, C-12), 121.1 (+, C-16), 112.9 (+, C-14), 56.1 (+, C-19) ppm; IR (ATR): 3057, 3022, 2843, 1595, 1563, 1501, 1349, 1284, 1207, 1152, 1041, 1013, 935, 851, 753, 730, 709, 673 cm^−1^; HR-ESI-MS: calcd for C_32_H_24_N_4_O_4_HgNa^+^ 813.1361. Found 813.1344.

#### Bis(6-ethoxycarbonyl-3-morpholinyl-sydnone imine-4-yl)-mercury(ii) (30b)

0.12 g (0.50 mmol) of 6-ethoxycarbonyl-3-morpholinyl-sydnone imine are dissolved in 10 mL of THF (abs.) under an inert atmosphere. Then 0.60 mL (0.60 mmol) of LHMDS (1 M in THF) are added at rt. The mixture is cooled to 0 °C treated with 0.08 g (0.30 mmol) of HgCl_2_. After stirring for 0.5 h at 0 °C the flask is warmed up to rt and stirred for another 3 h. The crude product is purified by column chromatography (EE). Yield: 0.13 g (78%) of a white solid, mp 243 °C (decomp.). ^1^H-NMR (600 MHz, CDCl_3_): *δ* = 3.99 (q, *J*_H,H_ = 7.1 Hz, 2H, 9-H), 3.87–3.89 (m, 4H, 13/13′-H), 3.64–3.66 (m, 4H, 12/12′-H), 1.26 (*t*, *J*_H,H_ = 7.1 Hz, 3H, 10-H) ppm; ^13^C-NMR (150 MHz, CDCl_3_): *δ* = 179.3 (o, C-5), 162.1 (o, C-7), 136.2 (o, C-4), 66.2 (−, C-13/13′), 61.2 (−, C-9), 56.5 (−, C-12/12′), 14.5 (+, C-10) ppm; IR (ATR): 2969, 2926, 2899, 2864, 1629, 1538, 1395, 1366, 1285, 1247, 1200, 1148, 1103, 1041, 970, 887, 786, 569 cm^−1^; HR-ESI-MS: calcd for C_18_H_26_N_8_O_8_HgNa^+^ 707.1478. Found 707.1483.

#### (5-Benzamide-3-(2-methoxyphenyl)-1,2,3-oxadiazolium-4-yl)triphenylborate (31a)

0.10 g (0.34 mmol) of 6-benzoyl-3-(2-methoxyphenyl)-sydnone imine are dissolved in 10 mL of THF (abs.) under an inert atmosphere. Then 0.41 mL (0.41 mmol) of LHMDS (1 M in THF) are added at rt. The mixture is cooled to −10 °C and treated with 1.63 mL (0.41 mmol) of BPh_3_ (0.25 M in THF). After stirring for 1 h at −10 °C the flask is warmed up to rt and stirred for another 3 h. The crude product is purified by column chromatography (EE : PE 1 : 5). Yield: 0.17 g (96%) of a white solid, mp 203 °C (decomp.). ^1^H-NMR (600 MHz, CDCl_3_): *δ* = 8.56 (s, 1H, 6-H), 7.50–7.52 (m, 1H, 11-H), 7.29–7.32 (m, 2H, 10/10′-H), 7.24–7.27 (m, 1H, 15-H), 7.16–7.17 (m, 6H, 21/21′-H), 7.06–7.09 (m, 6H, 22/22′-H), 7.02–7.04 (m, 5H, 23-H + 9/9′-H), 6.99–7.00 (m, 1H, 17-H), 6.75–6.78 (m, 1H, 16-H), 6.49–6.50 (m, 1H, 14-H), 3.58 (s, 3H, 19-H) ppm; ^13^C-NMR (150 MHz, CDCl_3_): *δ* = 166.0 (o, C-5), 162.7 (o, C-7), 152.9 (o, C-13), 152.4 (br. s, o, C-20), 142.4 (br. s, o, C-4), 135.3 (+, C-21/21′), 133.6 (+, C-11), 133.3 (+, C-15), 130.4 (o, C-8), 128.7 (+, C-10/10′), 127.8 (+, C-9/9′), 127.1 (+, C-17), 126.9 (+, C-22/22′), 124.5 (+, C-23), 122.5 (o, C-12), 120.0 (+, C-16), 111.5 (+, C-14), 55.3 (+, C-19) ppm; ^11^B-NMR (192 MHz, CDCl_3_): *δ* = −10.1 (s) ppm; IR (ATR): 3288, 3062, 3040, 2995, 1711, 1569, 1500, 1430, 1382, 1286, 1254, 1201, 1052, 1021, 745, 700, 644, 569 cm^−1^; HR-ESI-MS: calcd for C_34_H_28_N_3_O_3_BNa^+^ 560.2121. Found 560.2122.

#### (5-Ethylcarbamate-3-morpholinyl-1,2,3-oxadiazolium-4-yl)triphenylborate (31b)

0.10 g (0.41 mmol) of 6-ethoxycarbonyl-3-morpholinyl-sydnone imine are dissolved in 10 mL of THF (abs.) under an inert atmosphere. Then 0.50 mL (0.50 mmol) of LHMDS (1 M in THF) are added at rt. The mixture is cooled to −10 °C treated with 1.98 mL (0.50 mmol) of BPh_3_ (0.25 M in THF). After stirring for 1 h at −10 °C the flask is warmed up to rt and stirred for another 3 h. The crude product is purified by column chromatography (EE : PE 1 : 2). Yield: 0.17 g (87%) of a white solid, mp 121 °C (decomp.). ^1^H-NMR (600 MHz, CDCl_3_): *δ* = 7.33–7.35 (m, 6H, 16/16′-H), 7.20–7.22 (m, 6H, 17/17′-H), 7.11–7.13 (m, 3H, 18-H), 6.98 (s, 1H, 6-H), 4.12 (q, *J*_H,H_ = 7.1 Hz, 2H, 9-H), 3.24 (br. s, 4H, 13/13′-H), 3.00 (br. s, 4H, 12/12′-H), 1.20 (*t*, *J*_H,H_ = 7.1 Hz, 3H, 10-H) ppm; ^13^C-NMR (150 MHz, CDCl_3_): *δ* = 165.9 (o, C-5), 152.7 (o, C-15), 149.7 (o, C-7), 136.7 (o, C-4), 135.1 (+, C-16/16′), 127.2 (+, C-17/17′), 124.8 (+, C-18), 65.7 (−, C-13/13′), 63.4 (−, C-9), 56.1 (−, C-12/12′), 13.9 (+, C-10) ppm; ^11^B-NMR (192 MHz, CDCl_3_): *δ* = −10.2 (s) ppm; IR (ATR): 3302, 3062, 2980, 2920, 2866, 1757, 1595, 1428, 1401, 1227, 1193, 1099, 1034, 892, 761, 733, 709, 651, 622, 550 cm^−1^; HR-ESI-MS: calcd for C_27_H_29_N_4_O_4_BNa^+^ 507.2174. Found 507.2148.

## Conclusions

In conclusion the anionic N-heterocyclic carbenes derived from sydnone imines can be trapped by sulfur. These unstable sulfides can then be stabilized *in situ* upon methylation or by formation of transition metal complexes (Au, Hg). Furthermore, deprotonated sydnone imines are suitable for covalent bond formation towards boron and for the formation of stable mercury(ii) complexes in good yields. Structure determination was achieved by X-ray analysis.

## Conflicts of interest

There are no conflicts to declare.

## Supplementary Material

RA-009-C9RA00294D-s001

RA-009-C9RA00294D-s002
