# Supplementary material for: Sulfur, mercury, and boron adducts of sydnone imine derived anionic N-heterocyclic carbenes
Source: RSC Adv. 2019 Feb 6;9(9):4781–8. doi: 10.1039/c9ra00294d (PMC9060589; doi:10.1039/c9ra00294d)
Supplement: RA-009-C9RA00294D-s001 [file RA-009-C9RA00294D-s001.pdf]

Electronic Supplementary Material (ESI) for RSC Advances.
This journal is © The Royal Society of Chemistry 2019

Heterocycle syntheses with anionic N-heterocyclic carbenes. Ring transformations of sydnone imine anions

Tyll Freese,[a] Jan C. Namyslo,[a] Martin Nieger,[b] and Andreas Schmidt*[a]

[a] Clausthal University of Technology, Institute of Organic Chemistry, Leibnizstrasse 6, D-38678 Clausthal-Zellerfeld, Germany.

[b] University of Helsinki, Department of Chemistry, P.O. Box 55 (A.I. Virtasen aukio 1),
FIN-00014 University of Helsinki, Finland.

Contents

1. NMR Spectra ………………………..…………………………… 2
2. Crystal Structure Determinations ……………………………… 17

[a] Tyll Freese, Jan C. Namyslo, Andreas Schmidt
Institute of Organic Chemistry
Clausthal University of Technology
Leibnizstrasse 6, D-38678 Clausthal-Zellerfeld, Germany
E-mail: schmidt@ioc.tu-clausthal.de
[b] Martin Nieger
Department of Chemistry, Laboratory of Inorganic Chemistry
University of Helsinki
P.O Box 55, Fin-00014, Finnland

1. NMR Spectra

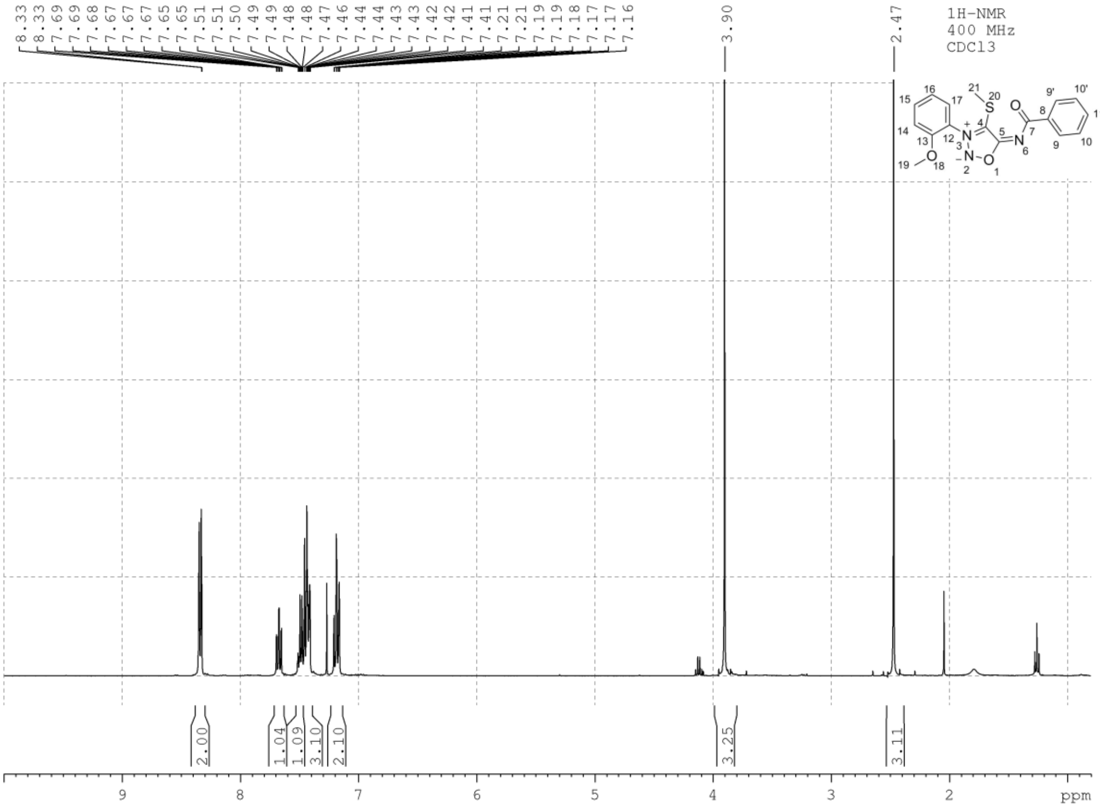

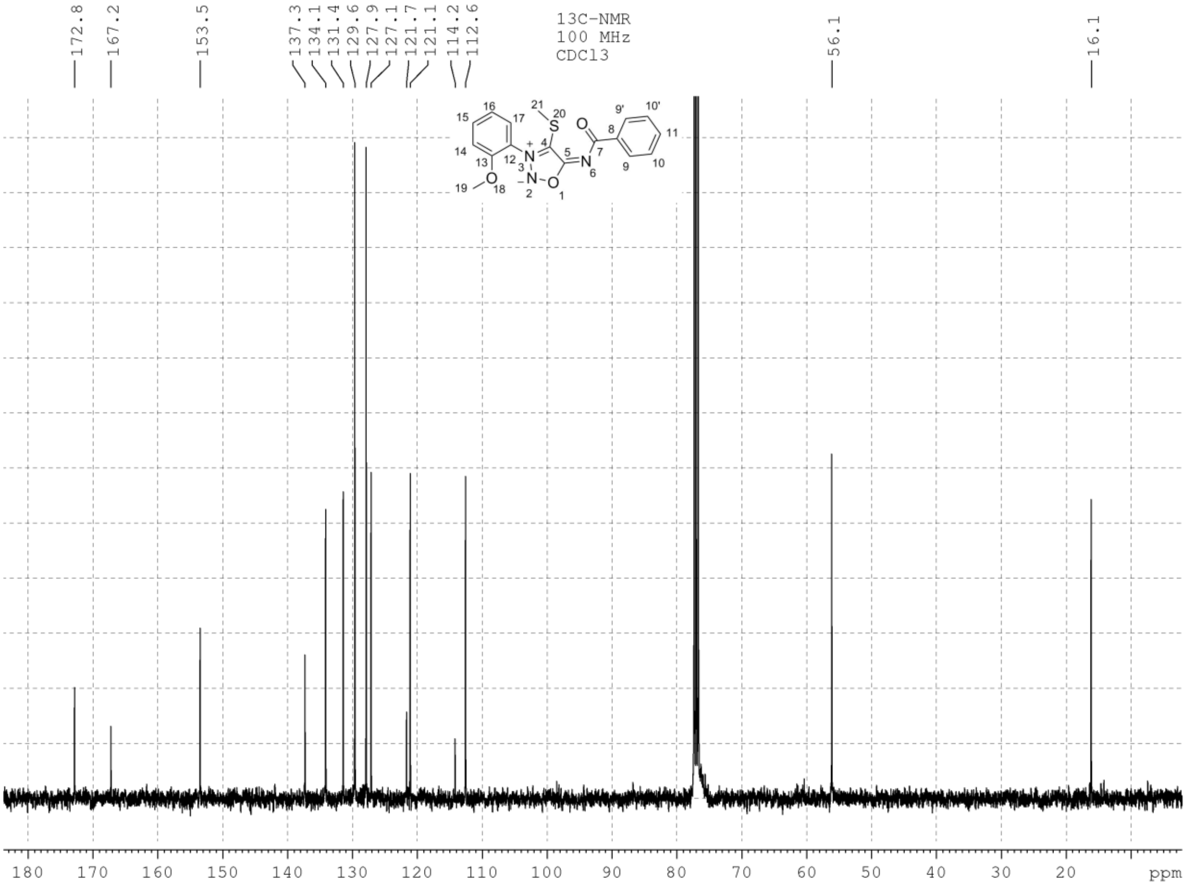

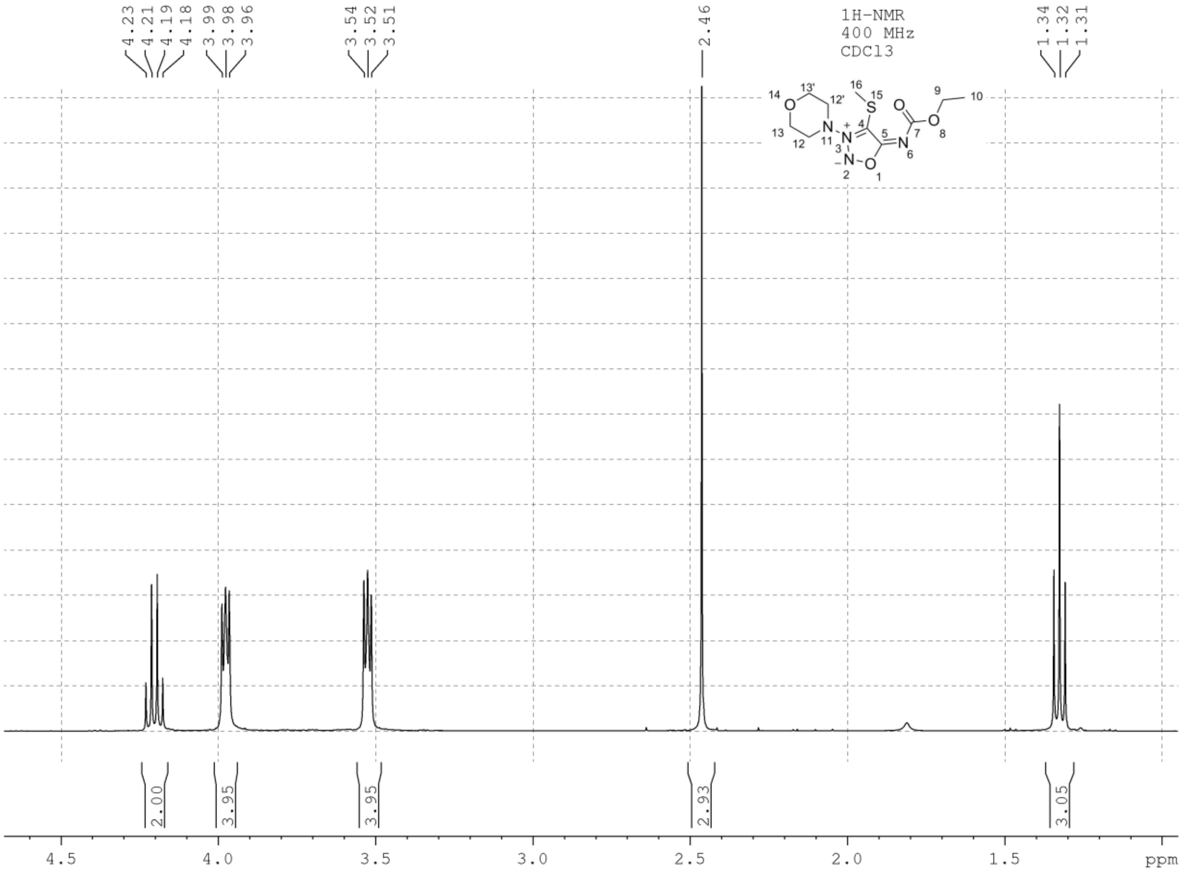
4.23 4.21 4.19 4.18 3.99 3.98 3.96 3.54 3.52 3.51 2.46 1.34 1.32 1.31
1H-NMR
400 MHz
CDCl3
2.00 3.95 3.95 2.93 3.05
4.5 4.0 3.5 3.0 2.5 2.0 1.5 ppm

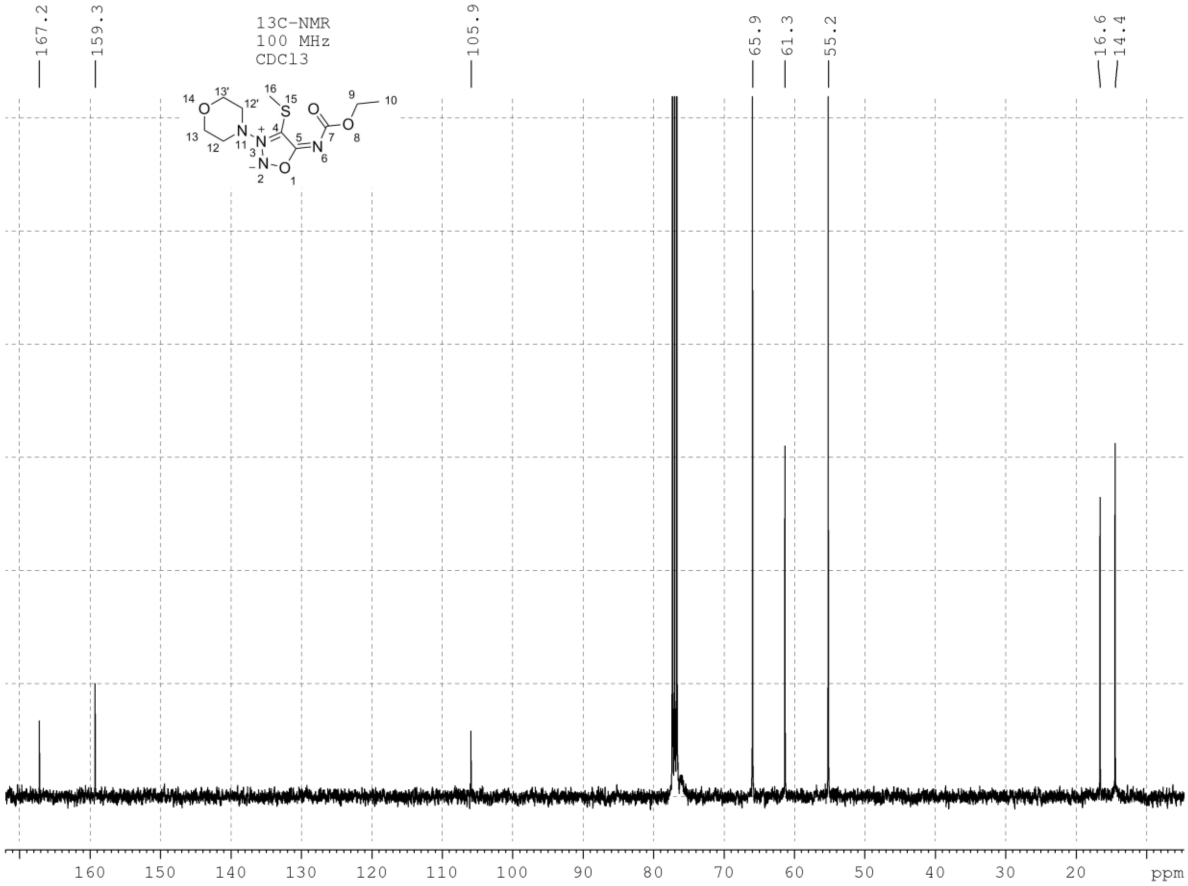
167.2 159.3 105.9 65.9 61.3 55.2 16.6 14.4
13C-NMR
100 MHz
CDCl3
160 150 140 130 120 110 100 90 80 70 60 50 40 30 20 ppm

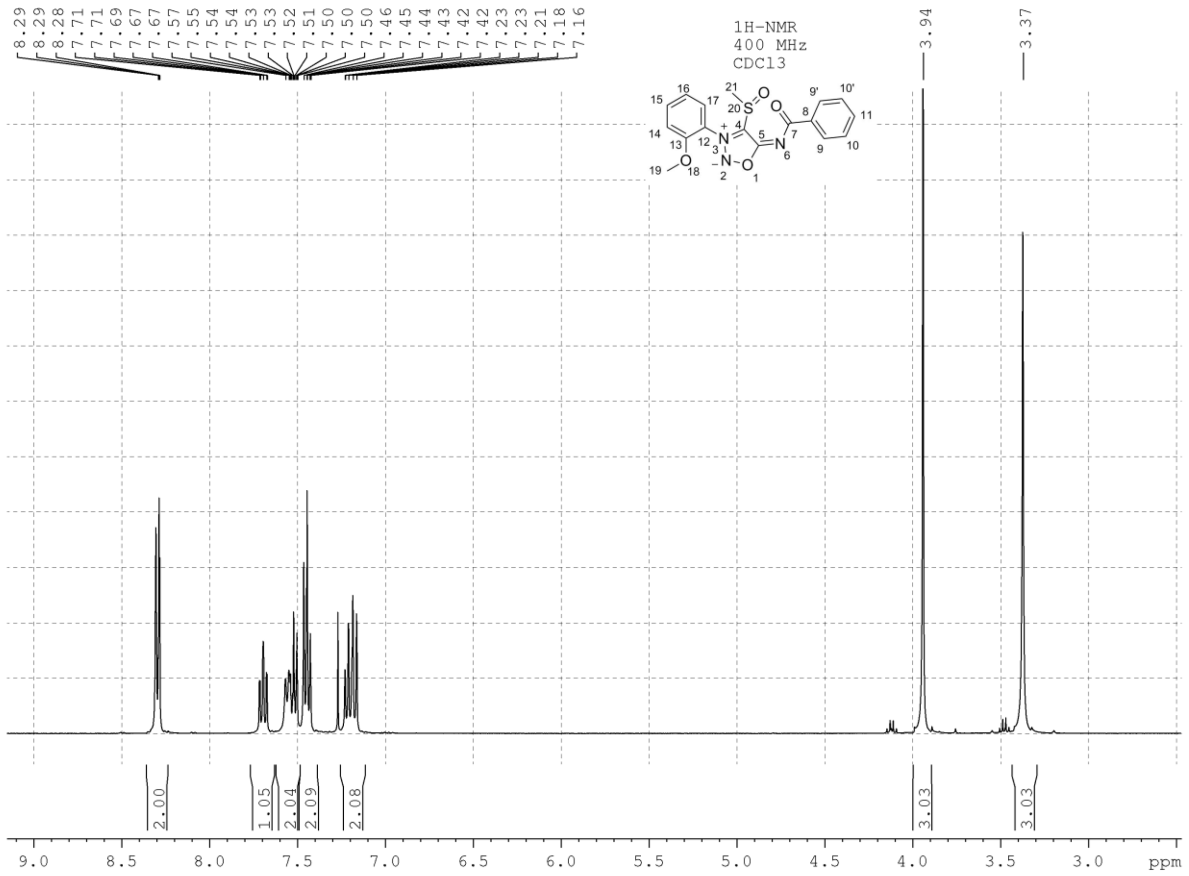
8.29
8.29
8.28
7.71
7.71
7.69
7.67
7.67
7.57
7.55
7.54
7.54
7.53
7.53
7.52
7.51
7.50
7.50
7.50
7.46
7.45
7.44
7.43
7.42
7.42
7.23
7.23
7.21
7.18
7.16
1H-NMR
400 MHz
CDCl3
3.94
3.37
2.00
1.05
2.04
2.09
2.08
3.03
3.03
ppm

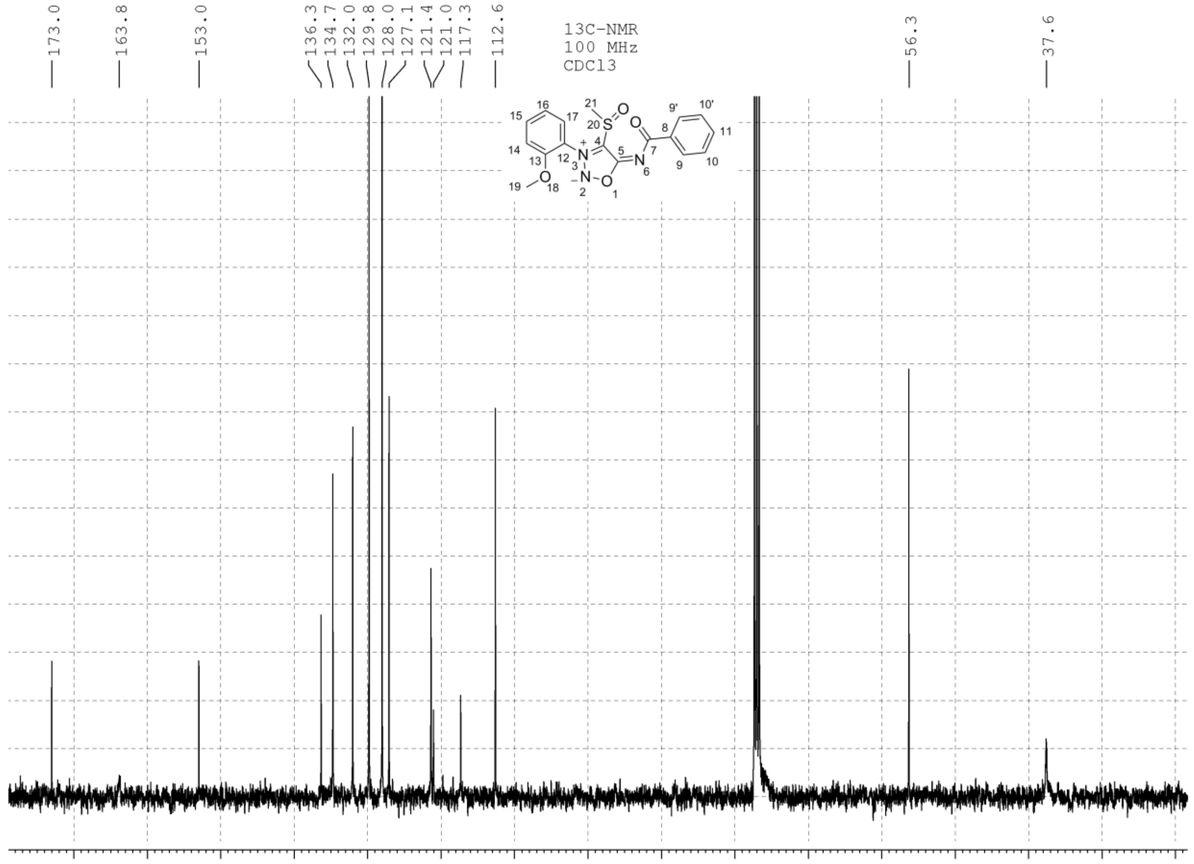
173.0
163.8
153.0
136.3
134.7
132.0
129.8
128.0
127.1
121.4
121.0
117.3
112.6
13C-NMR
100 MHz
CDCl3
56.3
37.6
ppm

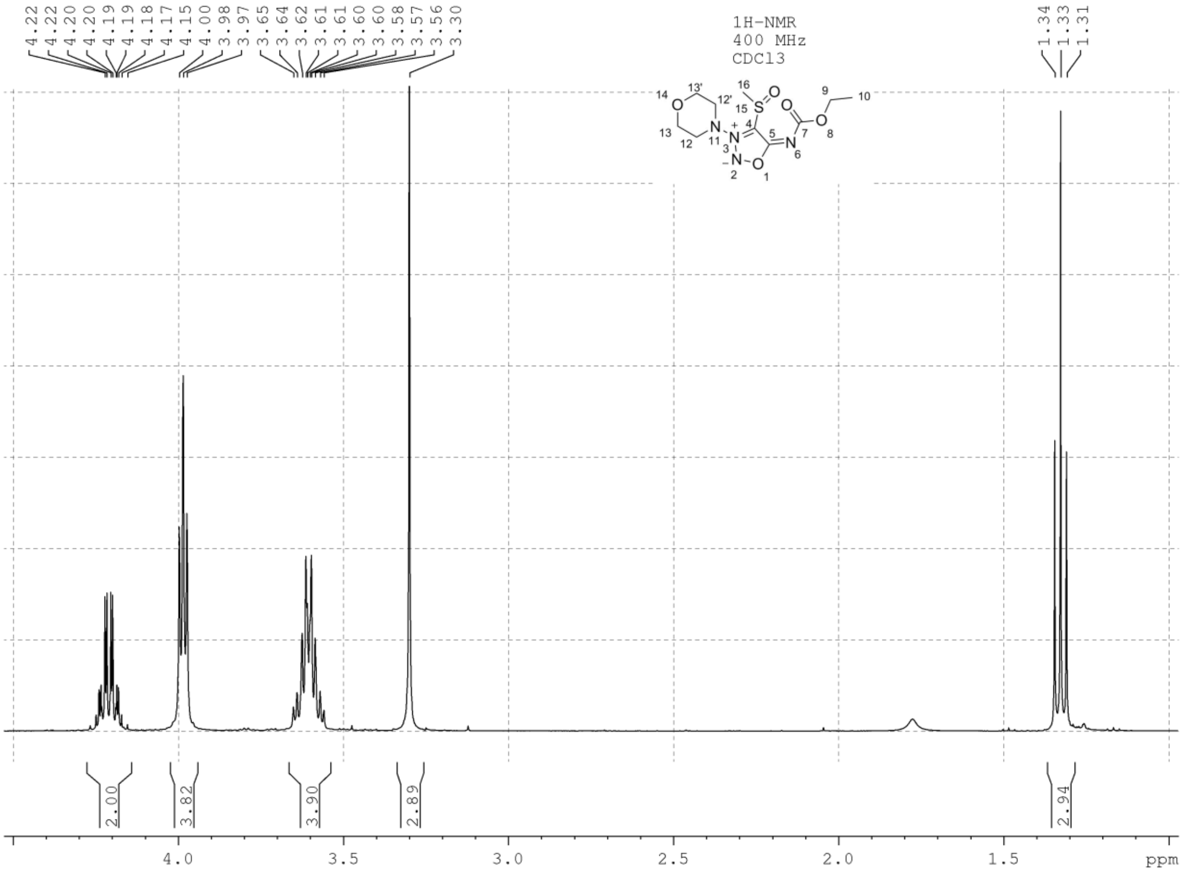
4.22 4.22 4.20 4.20 4.19 4.19 4.18 4.17 4.15 4.00 3.98 3.97 3.65 3.64 3.62 3.61 3.61 3.60 3.58 3.57 3.56 3.30
1H-NMR
400 MHz
CDCl3
1.34 1.33 1.31
2.00
3.82
3.90
2.89
2.94
4.0
3.5
3.0
2.5
2.0
1.5
ppm

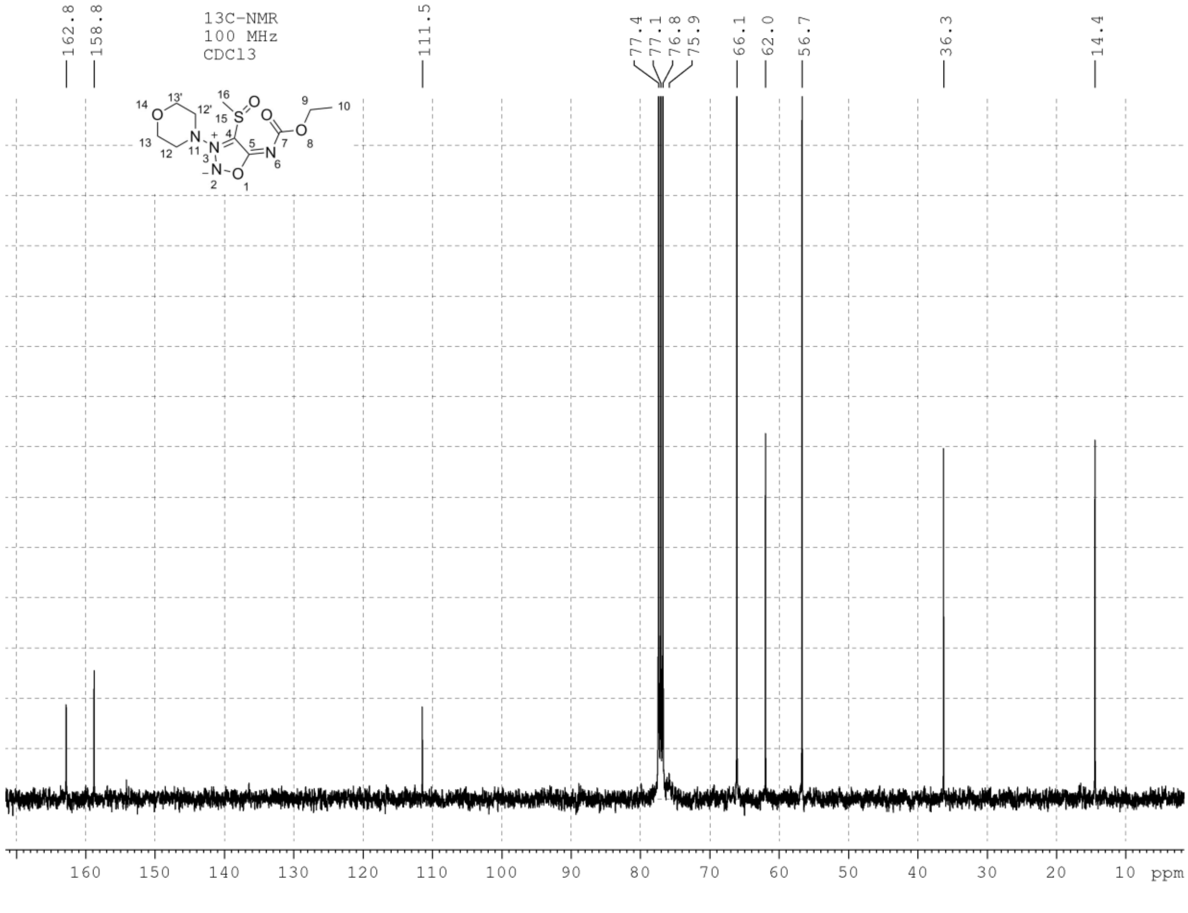
162.8
158.8
13C-NMR
100 MHz
CDCl3
111.5
77.4 77.1 76.8 75.9
66.1
62.0
56.7
36.3
14.4
160 150 140 130 120 110 100 90 80 70 60 50 40 30 20 10 ppm

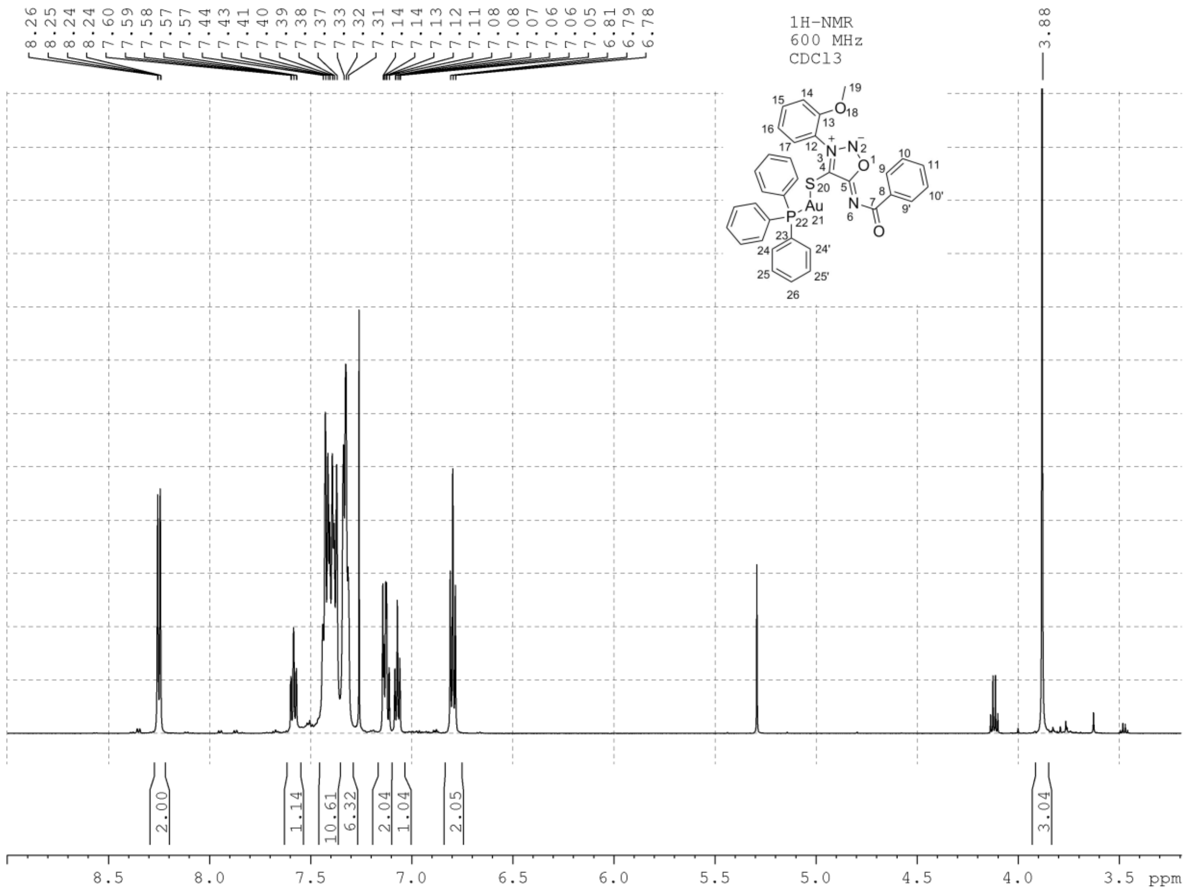
1H-NMR
600 MHz
CDCl3
8.26 8.25 8.24 8.24 7.60 7.59 7.58 7.57 7.44 7.43 7.41 7.40 7.39 7.38 7.37 7.33 7.32 7.31 7.14 7.14 7.13 7.12 7.11 7.08 7.08 7.07 7.06 7.05 6.81 6.79 6.78
3.88
2.00 1.14 10.61 6.32 2.04 1.04 2.05 3.04
8.5 8.0 7.5 7.0 6.5 6.0 5.5 5.0 4.5 4.0 3.5 ppm

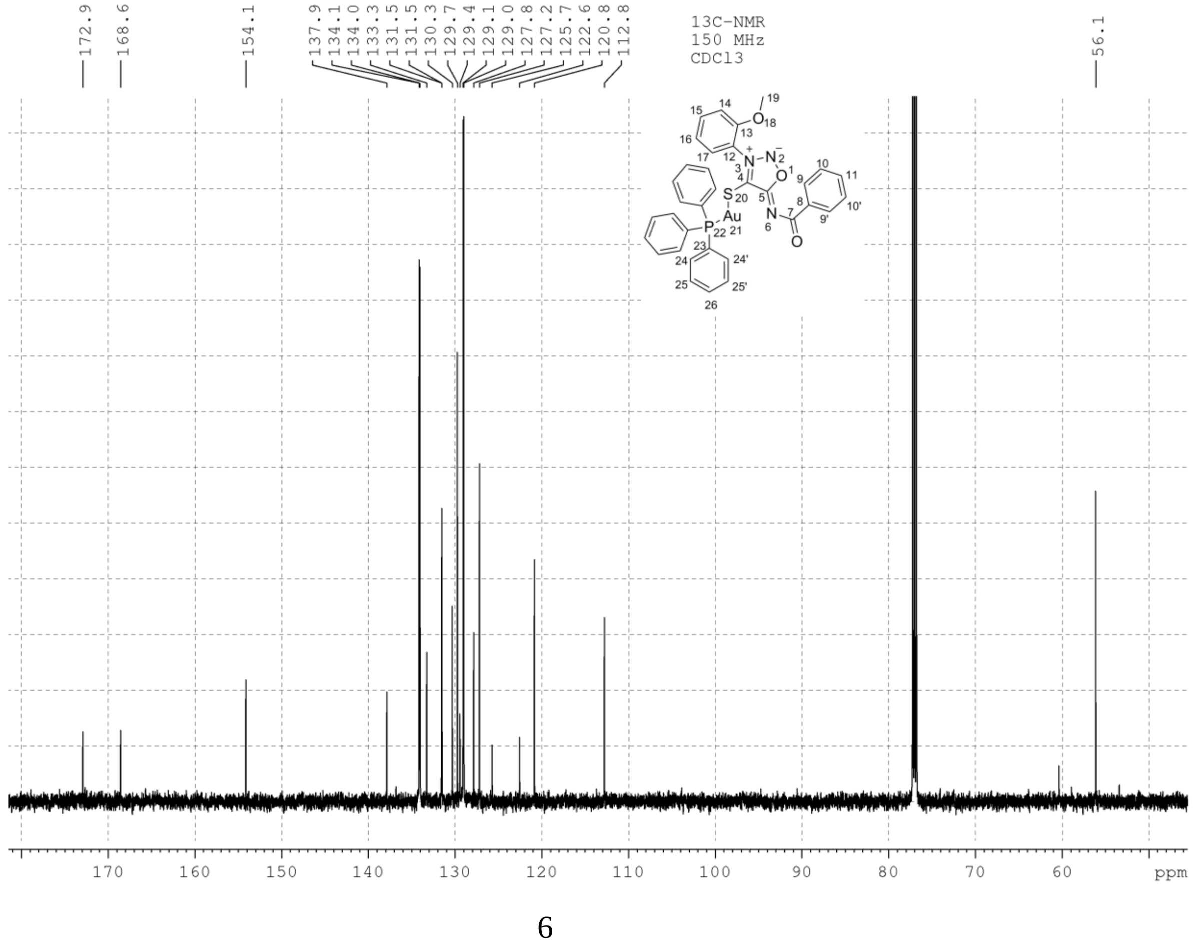
13C-NMR
150 MHz
CDCl3
172.9 168.6 154.1
137.9 134.1 134.0 133.3 131.5 131.5 130.3 129.7 129.4 129.1 129.0 127.8 127.2 125.7 122.6 120.8 112.8
56.1
170 160 150 140 130 120 110 100 90 80 70 60 ppm

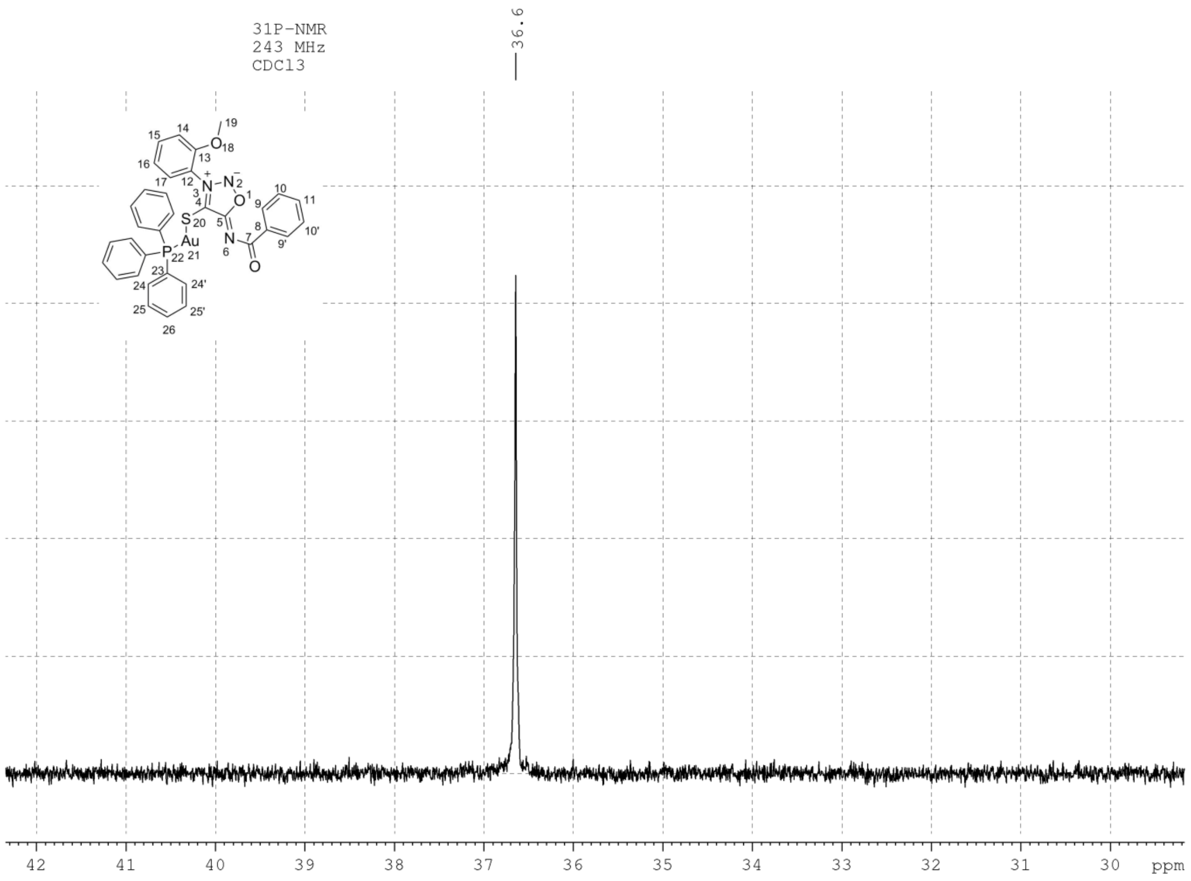
31P-NMR
243 MHz
CDCl3
36.6
ppm

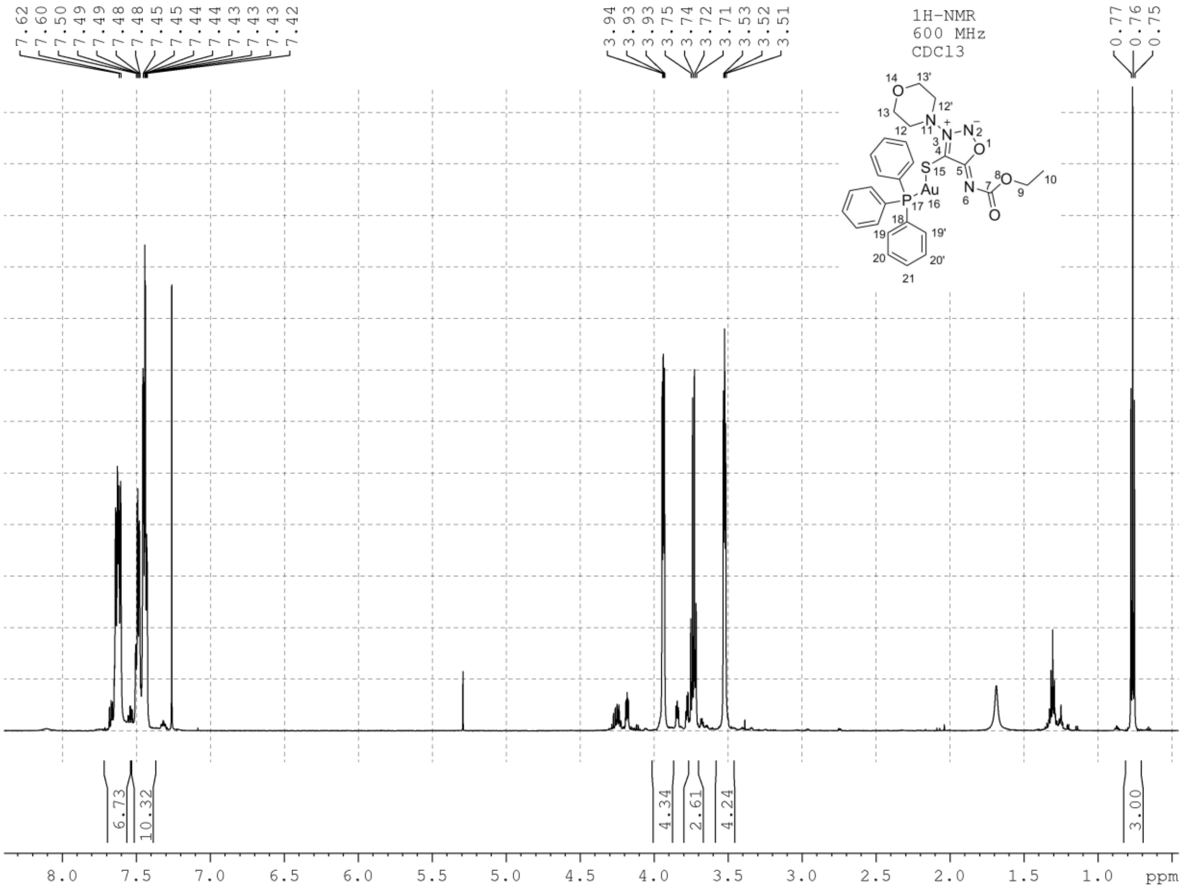
1H-NMR
600 MHz
CDCl3
7.62 7.60 7.50 7.49 7.49 7.48 7.48 7.45 7.45 7.44 7.44 7.43 7.43 7.43 7.43 7.42
3.94 3.93 3.93 3.75 3.74 3.72 3.71 3.53 3.52 3.51
0.77 0.76 0.75
6.73
10.32
4.34
2.61
4.24
3.00
ppm

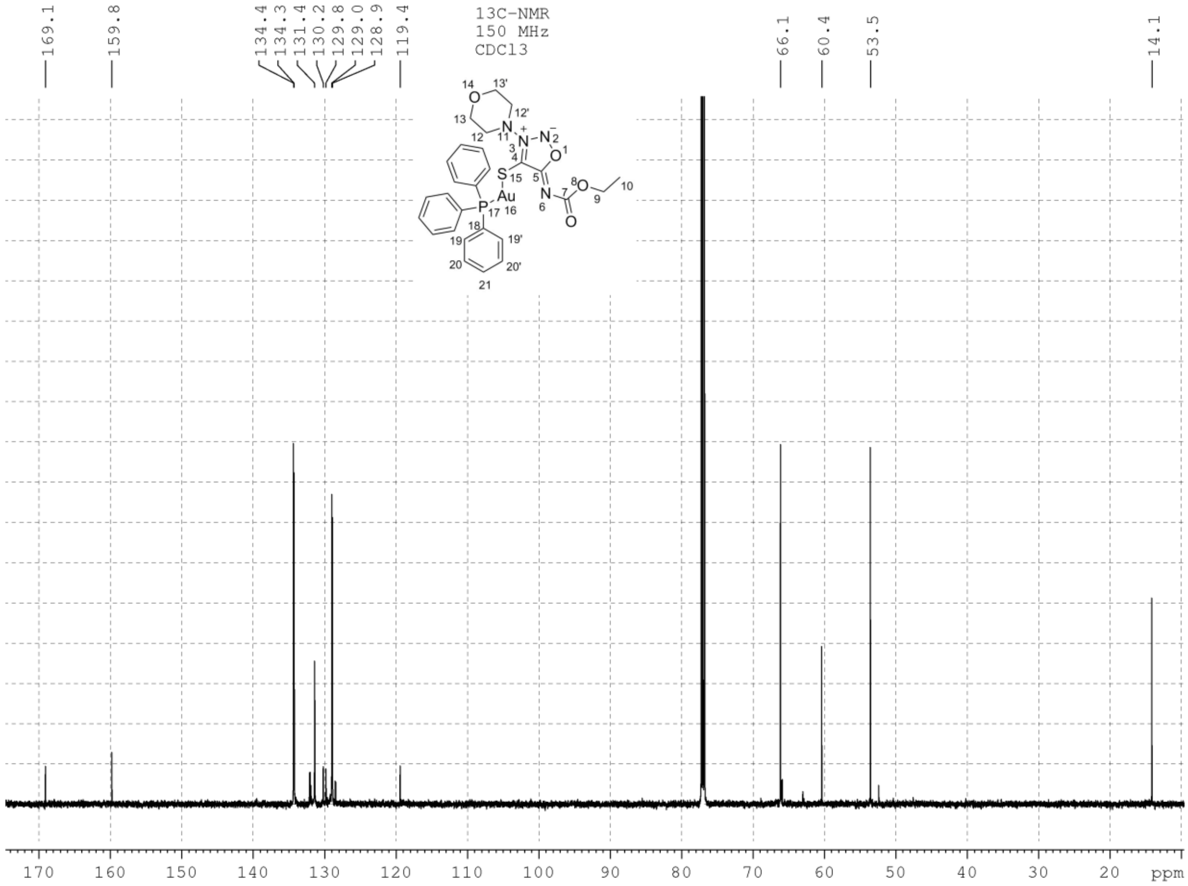

169.1
159.8
134.4
134.3
131.4
130.2
129.8
129.0
128.9
119.4
13C-NMR
150 MHz
CDCl3
66.1
60.4
53.5
14.1
ppm

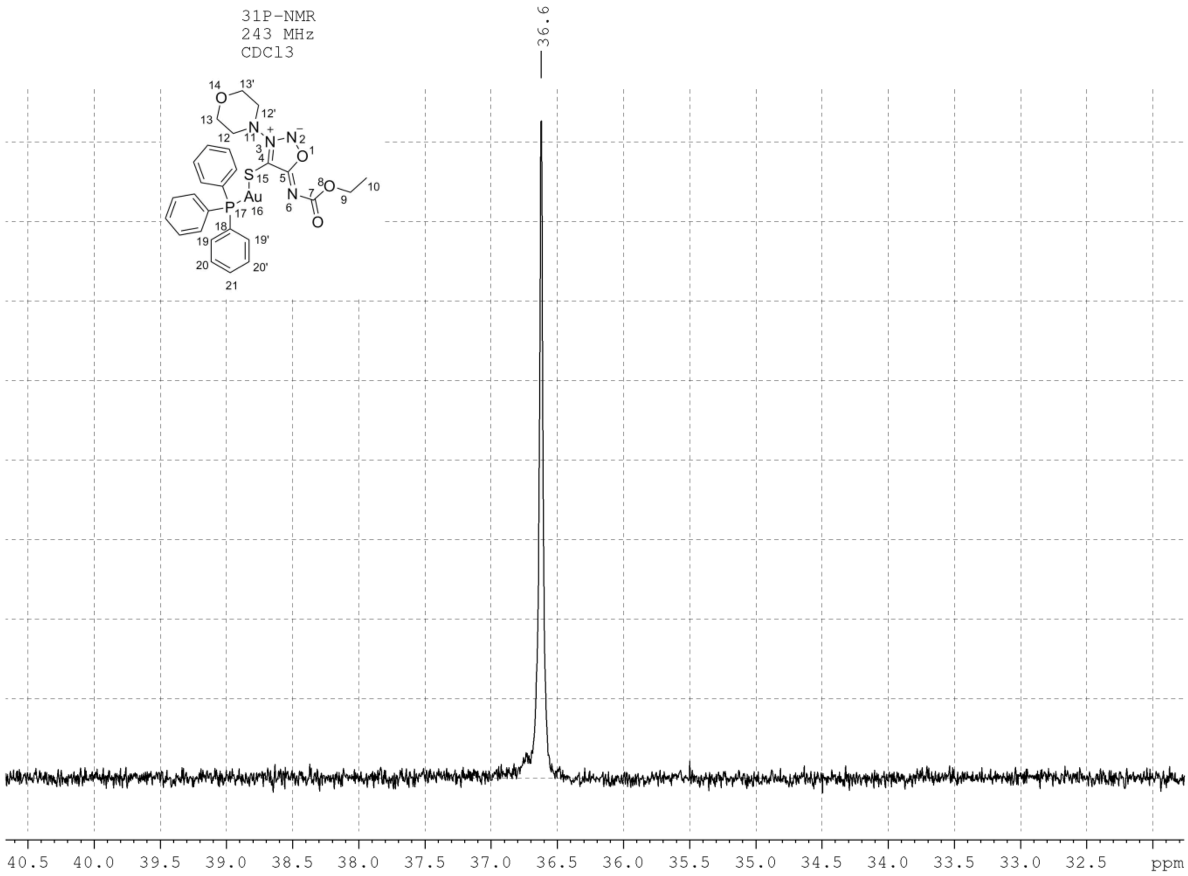

31P-NMR
243 MHz
CDCl3
36.6
ppm

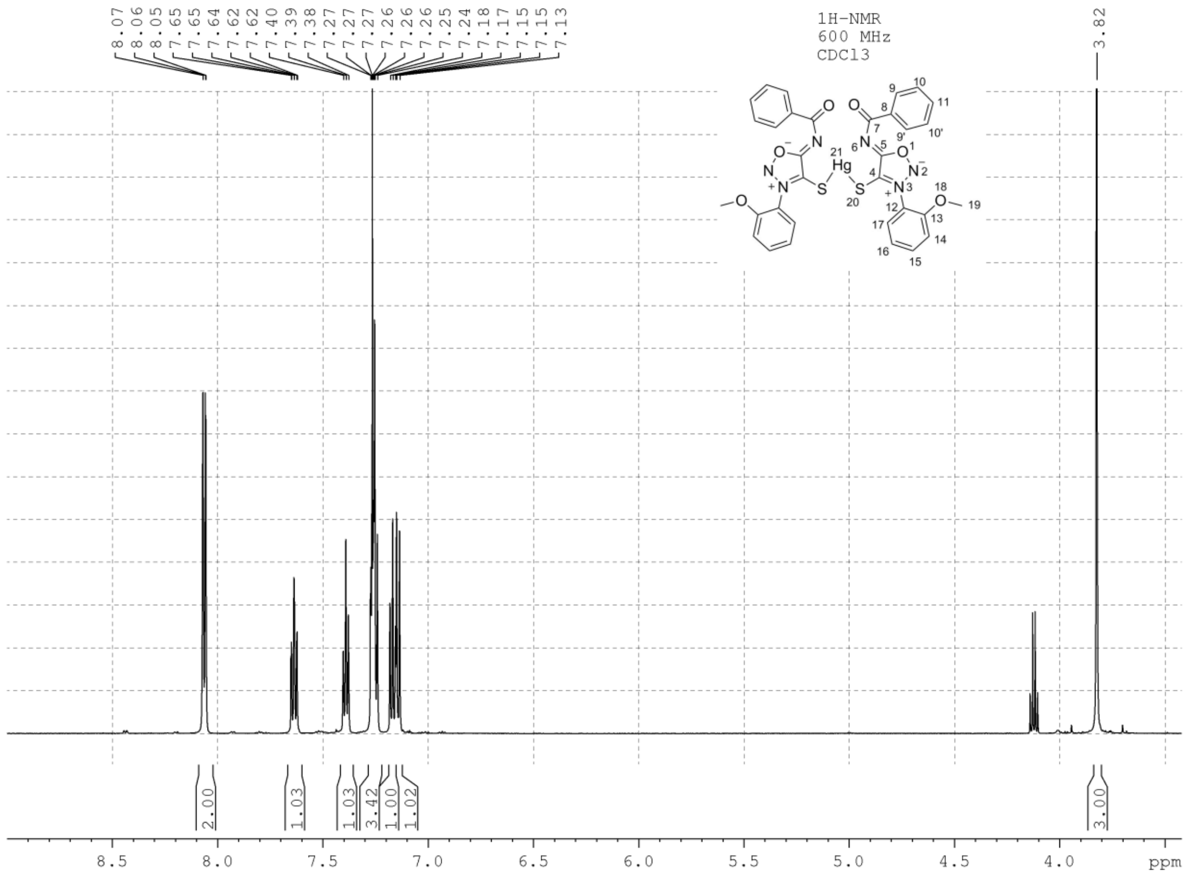
1H-NMR
600 MHz
CDCl3
8.07 8.06 8.05 7.65 7.64 7.62 7.62 7.40 7.39 7.38 7.27 7.27 7.27 7.26 7.26 7.26 7.25 7.24 7.18 7.17 7.15 7.15 7.13
3.82
2.00 1.03 1.03 3.42 1.00 1.02 3.00
8.5 8.0 7.5 7.0 6.5 6.0 5.5 5.0 4.5 4.0 ppm

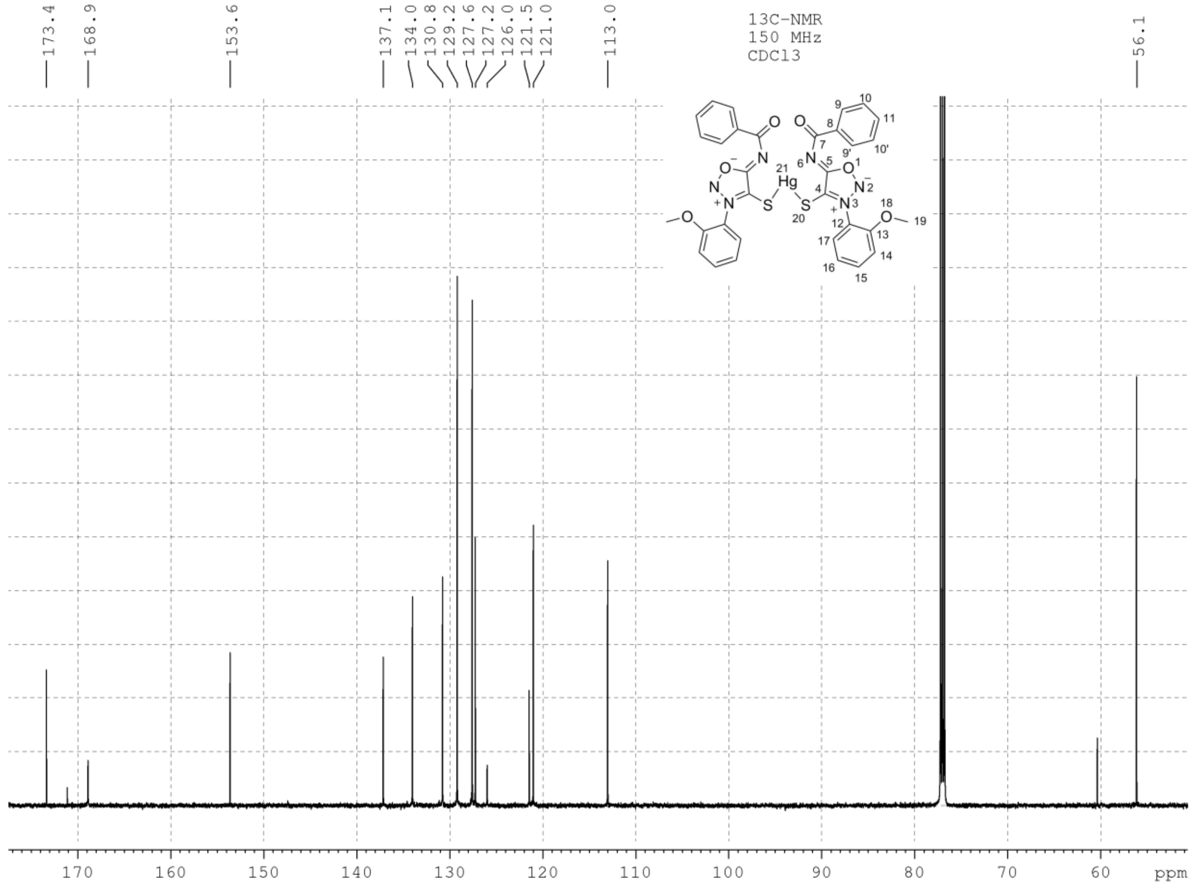
13C-NMR
150 MHz
CDCl3
173.4 168.9 153.6 137.1 134.0 130.8 129.2 127.6 127.2 126.0 121.5 121.0 113.0 56.1
170 160 150 140 130 120 110 100 90 80 70 60 ppm

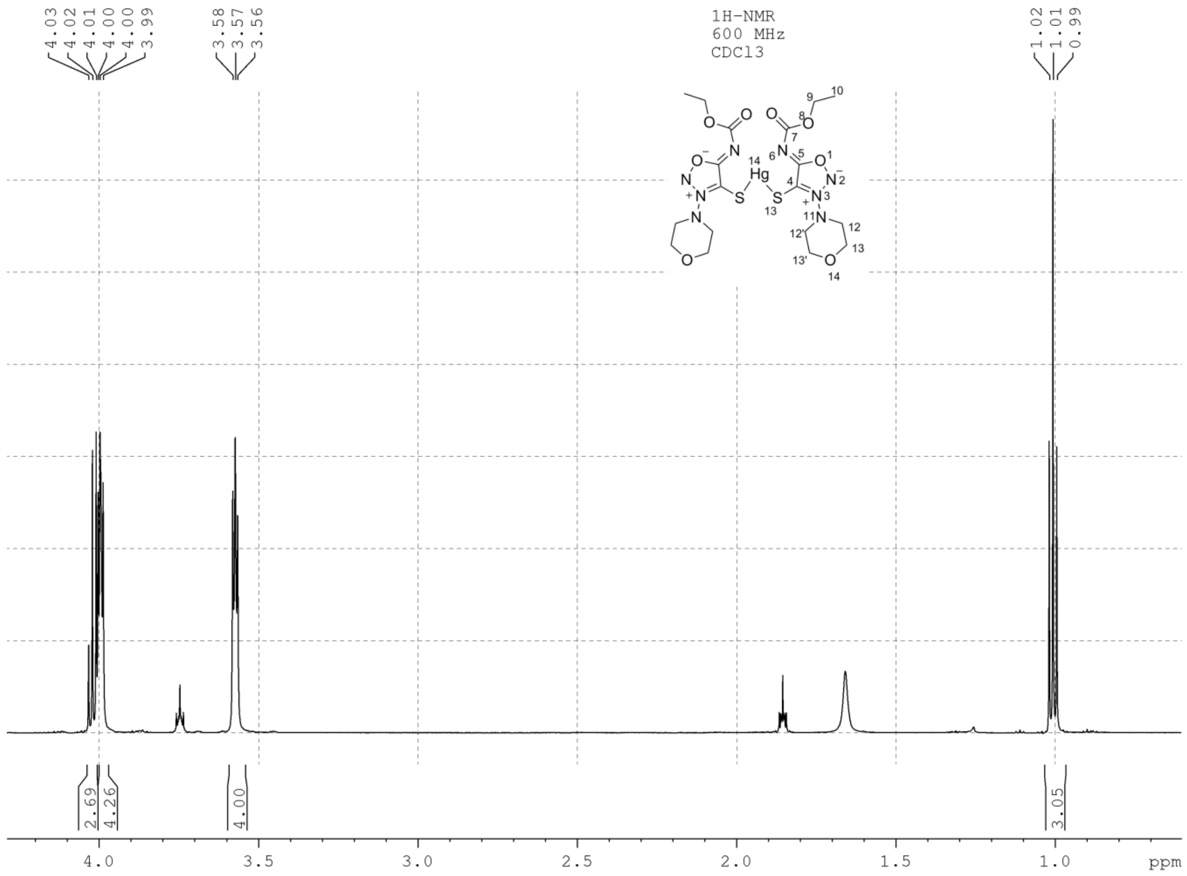
4.03
4.02
4.01
4.00
4.00
3.99
3.58
3.57
3.56
1H-NMR
600 MHz
CDCl3
1.02
1.01
0.99
2.69
4.26
4.00
3.05
4.0
3.5
3.0
2.5
2.0
1.5
1.0
ppm

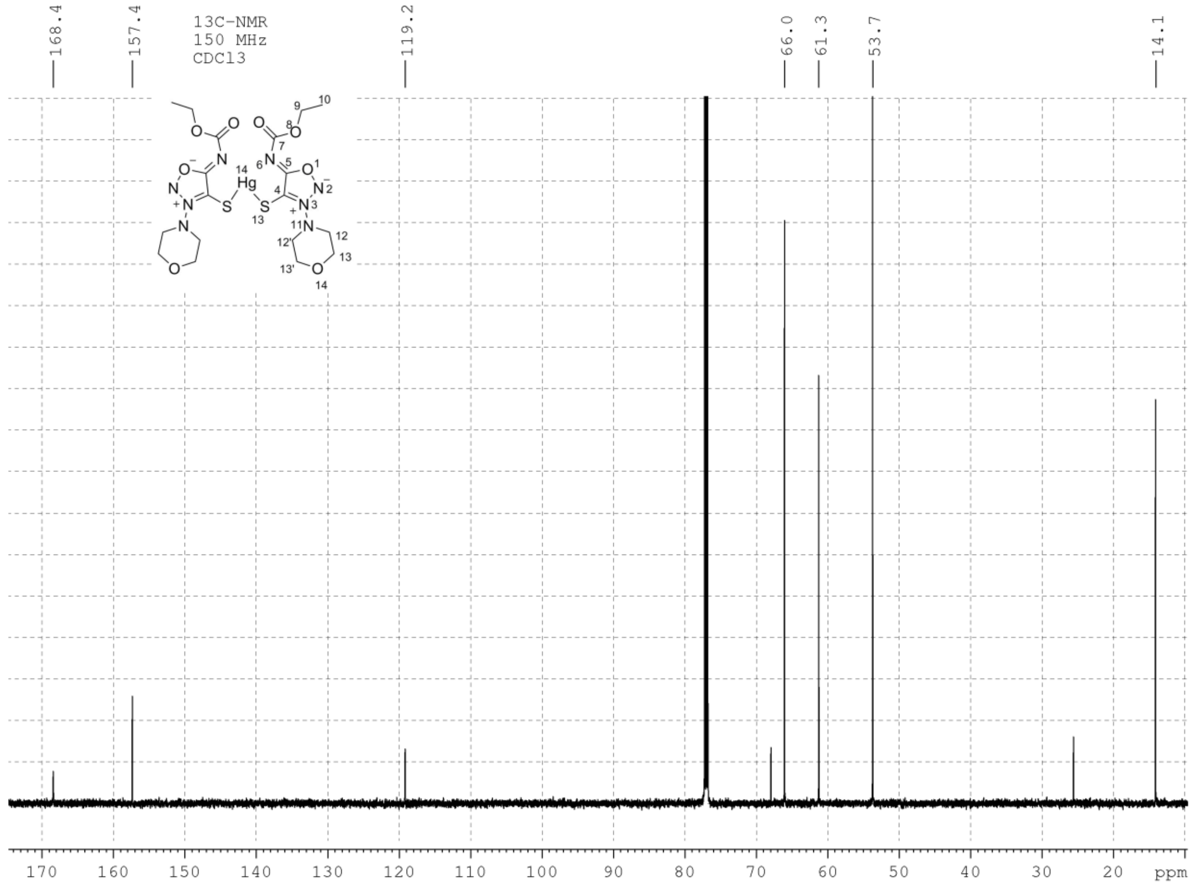
168.4
157.4
13C-NMR
150 MHz
CDCl3
119.2
66.0
61.3
53.7
14.1
170
160
150
140
130
120
110
100
90
80
70
60
50
40
30
20
ppm

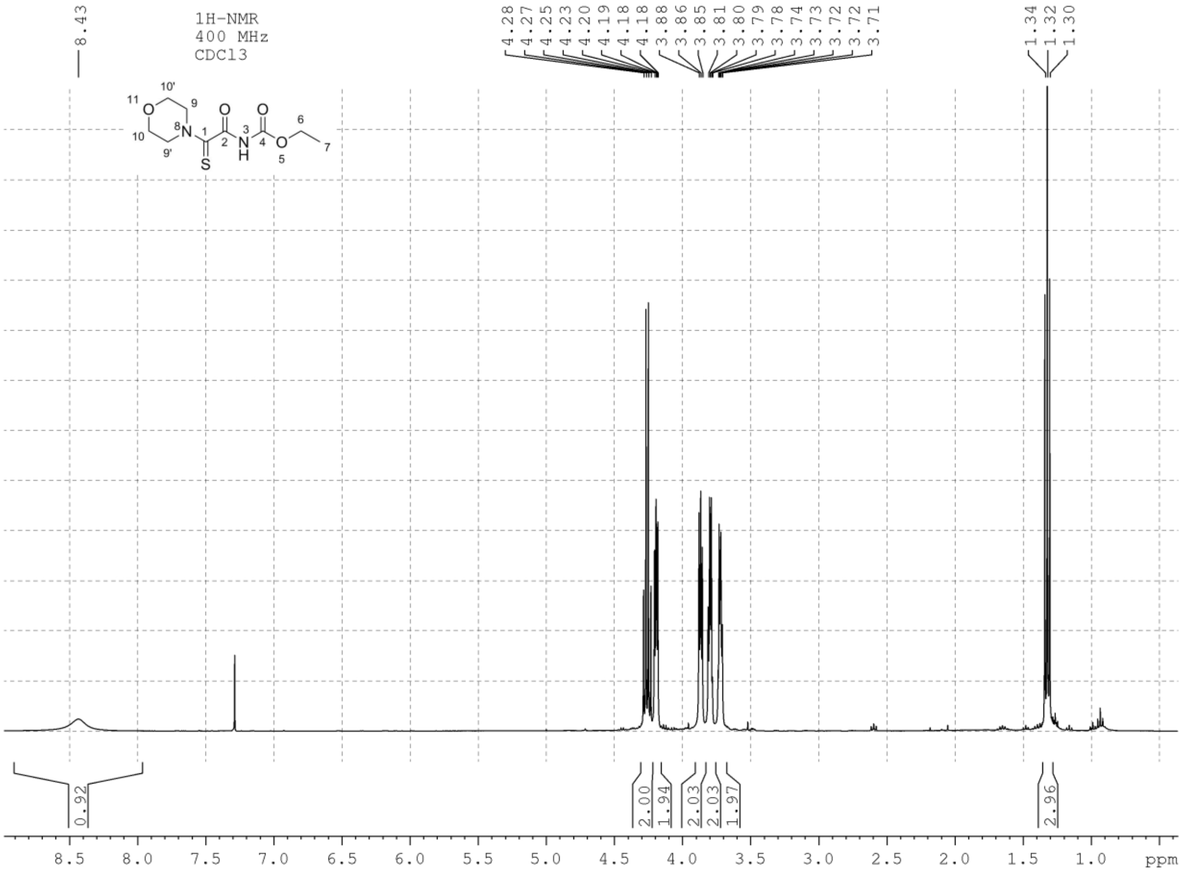
1H-NMR
400 MHz
CDCl3
8.43
4.28 4.27 4.25 4.23 4.20 4.19 4.18 4.18 3.88 3.86 3.85 3.81 3.80 3.79 3.78 3.74 3.73 3.72 3.72 3.71
1.34 1.32 1.30
0.92
2.00 1.94 2.03 2.03 1.97
2.96
8.5 8.0 7.5 7.0 6.5 6.0 5.5 5.0 4.5 4.0 3.5 3.0 2.5 2.0 1.5 1.0 ppm

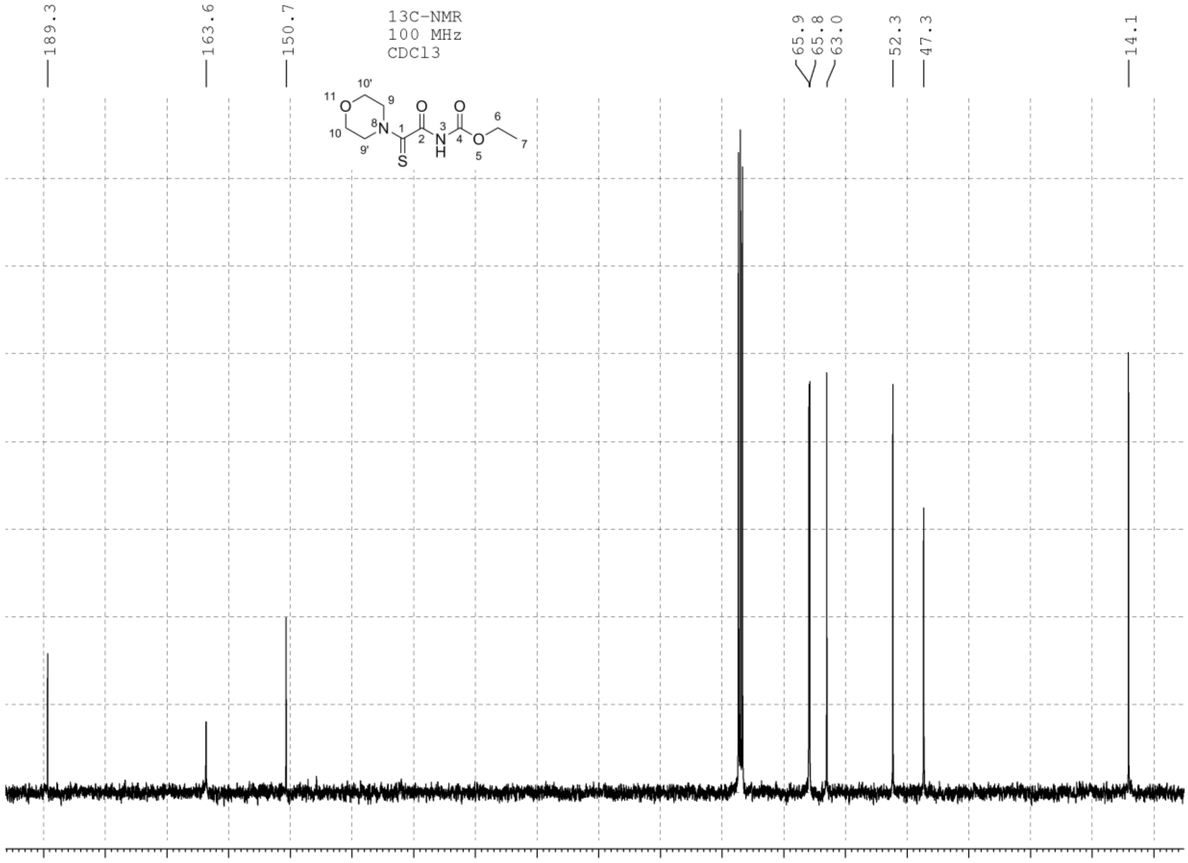
13C-NMR
100 MHz
CDCl3
189.3
163.6
150.7
65.9 65.8 63.0
52.3 47.3
14.1
190 180 170 160 150 140 130 120 110 100 90 80 70 60 50 40 30 20 ppm

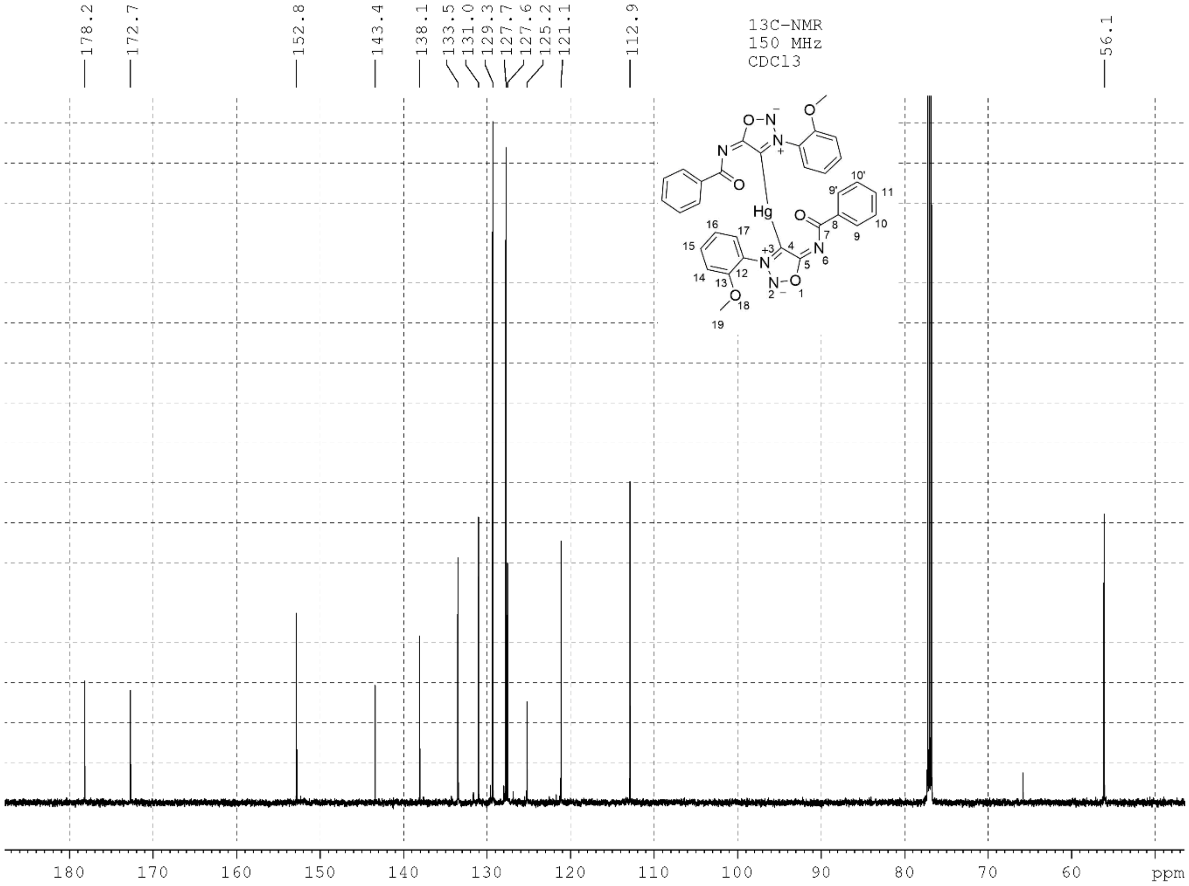
178.2
172.7
152.8
143.4
138.1
133.5
131.0
129.3
127.7
127.6
125.2
121.1
112.9
13C-NMR
150 MHz
CDCl3
56.1
180 170 160 150 140 130 120 110 100 90 80 70 60 ppm

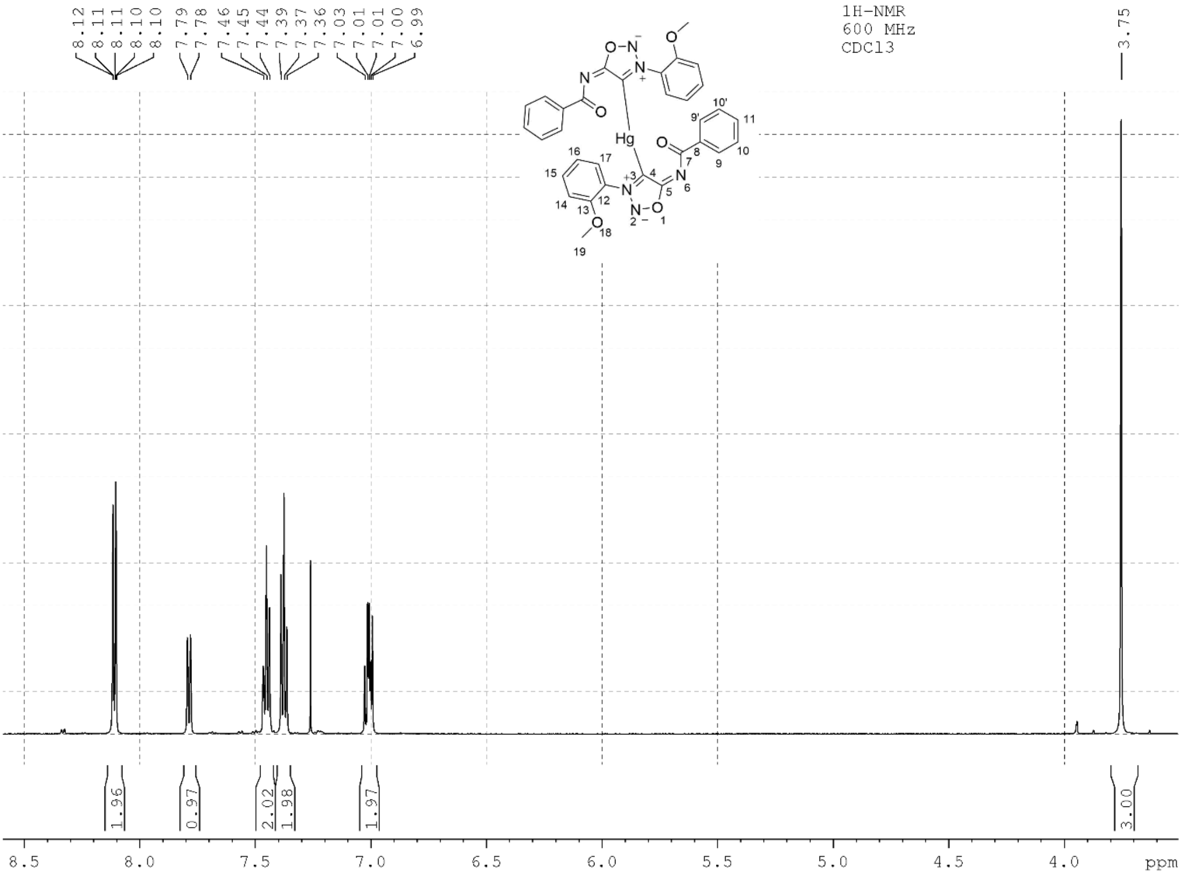
8.12
8.11
8.11
8.10
8.10
7.79
7.78
7.46
7.45
7.44
7.39
7.37
7.36
7.03
7.01
7.01
7.00
6.99
1H-NMR
600 MHz
CDCl3
3.75
1.96
0.97
2.02
1.98
1.97
3.00
8.5 8.0 7.5 7.0 6.5 6.0 5.5 5.0 4.5 4.0 ppm

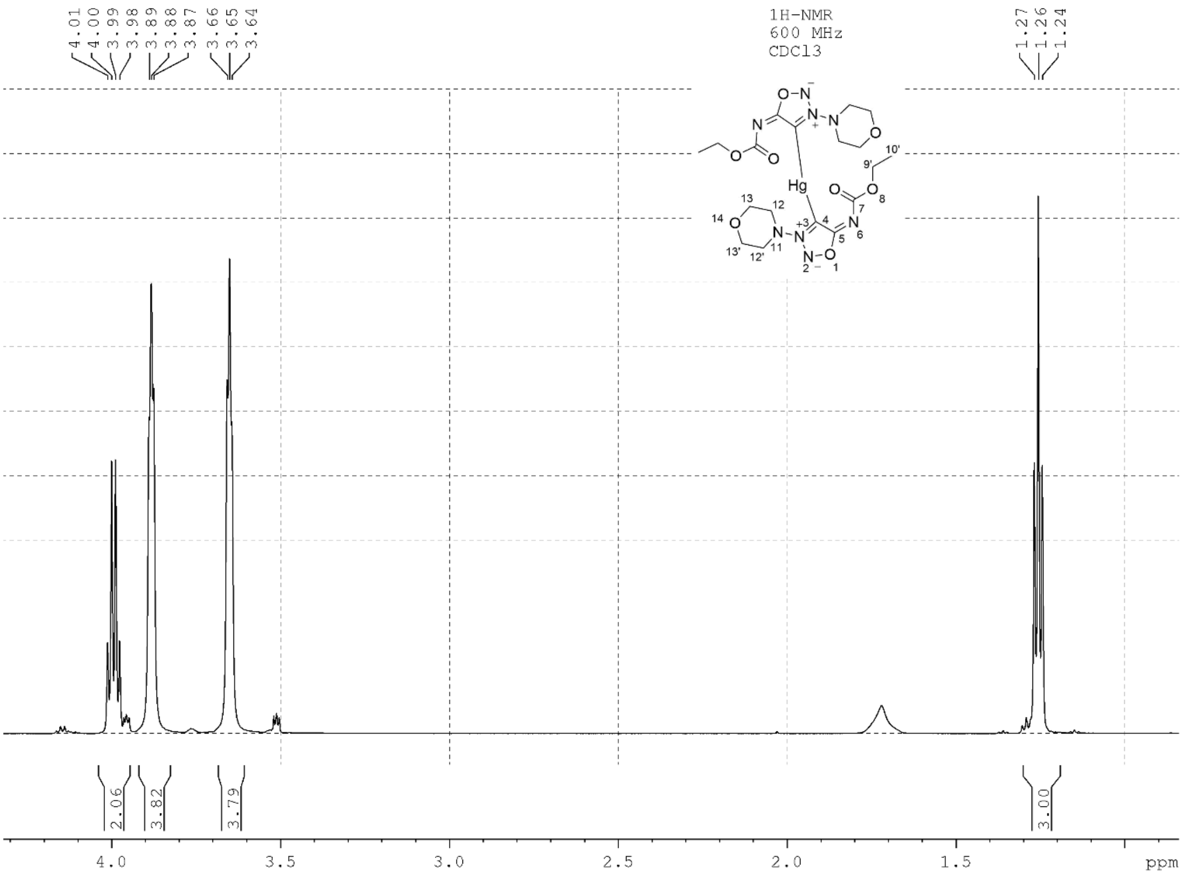
1H-NMR
600 MHz
CDCl3
4.01
4.00
3.99
3.98
3.89
3.88
3.87
3.66
3.65
3.64
1.27
1.26
1.24
2.06
3.82
3.79
3.00
ppm

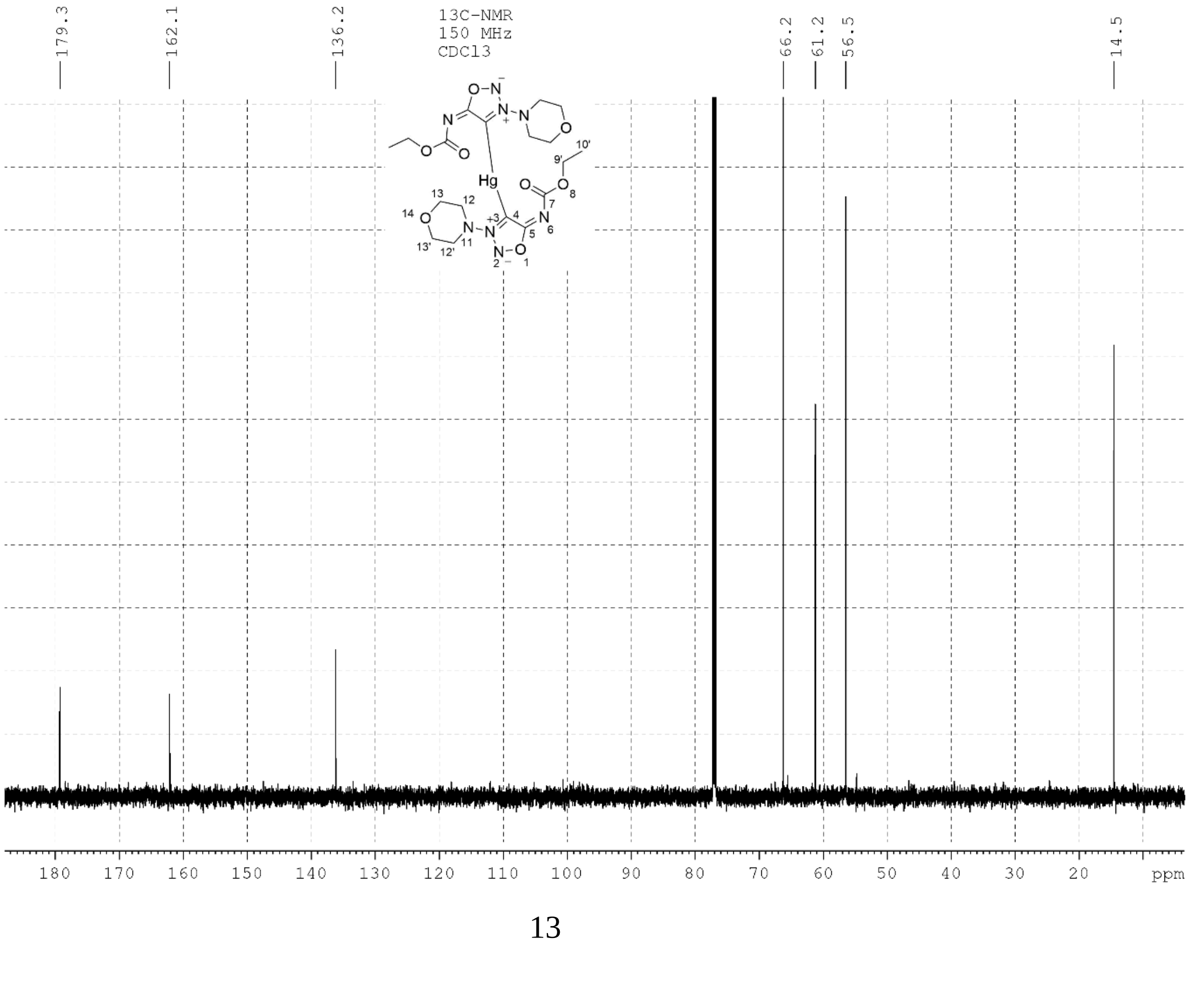
13C-NMR
150 MHz
CDCl3
179.3
162.1
136.2
66.2
61.2
56.5
14.5
ppm

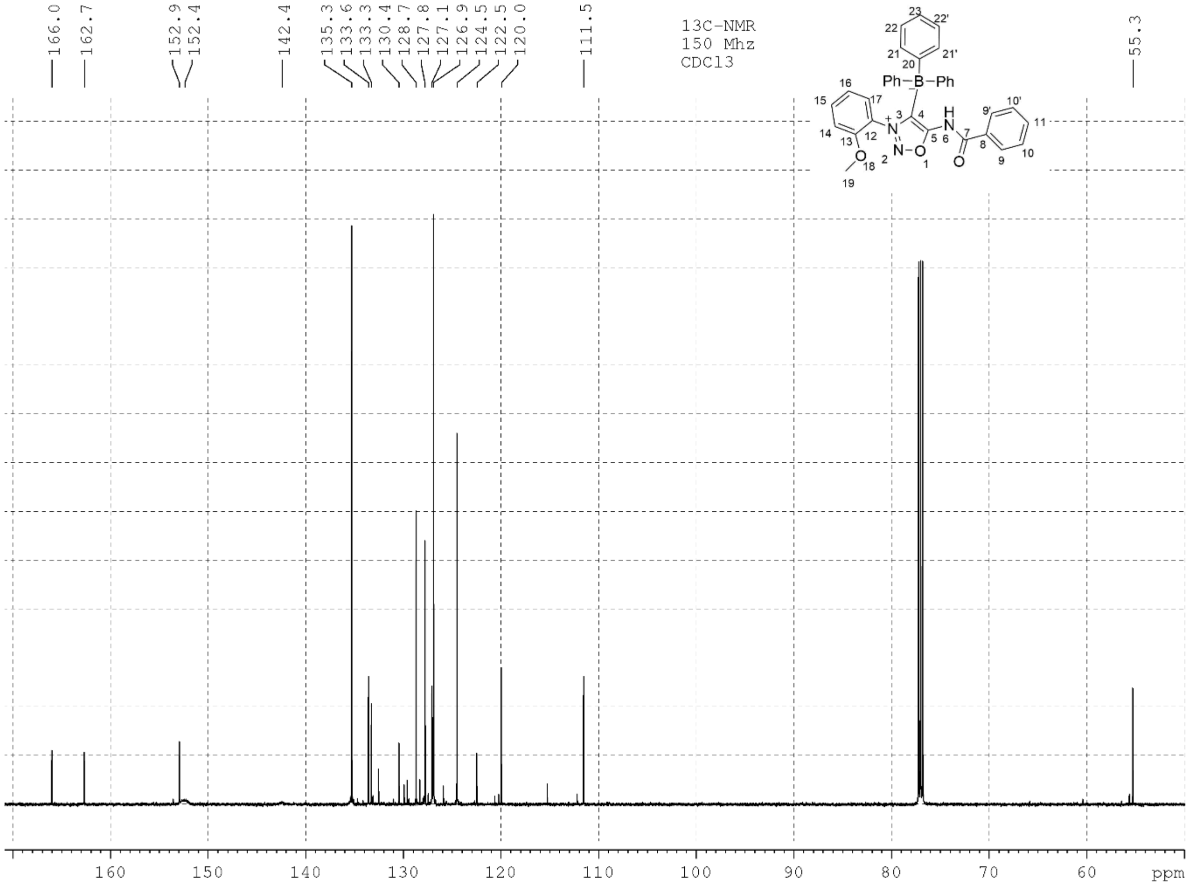
13C-NMR
150 Mhz
CDCl3
166.0
162.7
152.9
152.4
142.4
135.3
133.6
133.3
130.4
128.7
127.8
127.1
126.9
124.5
122.5
120.0
111.5
55.3
ppm

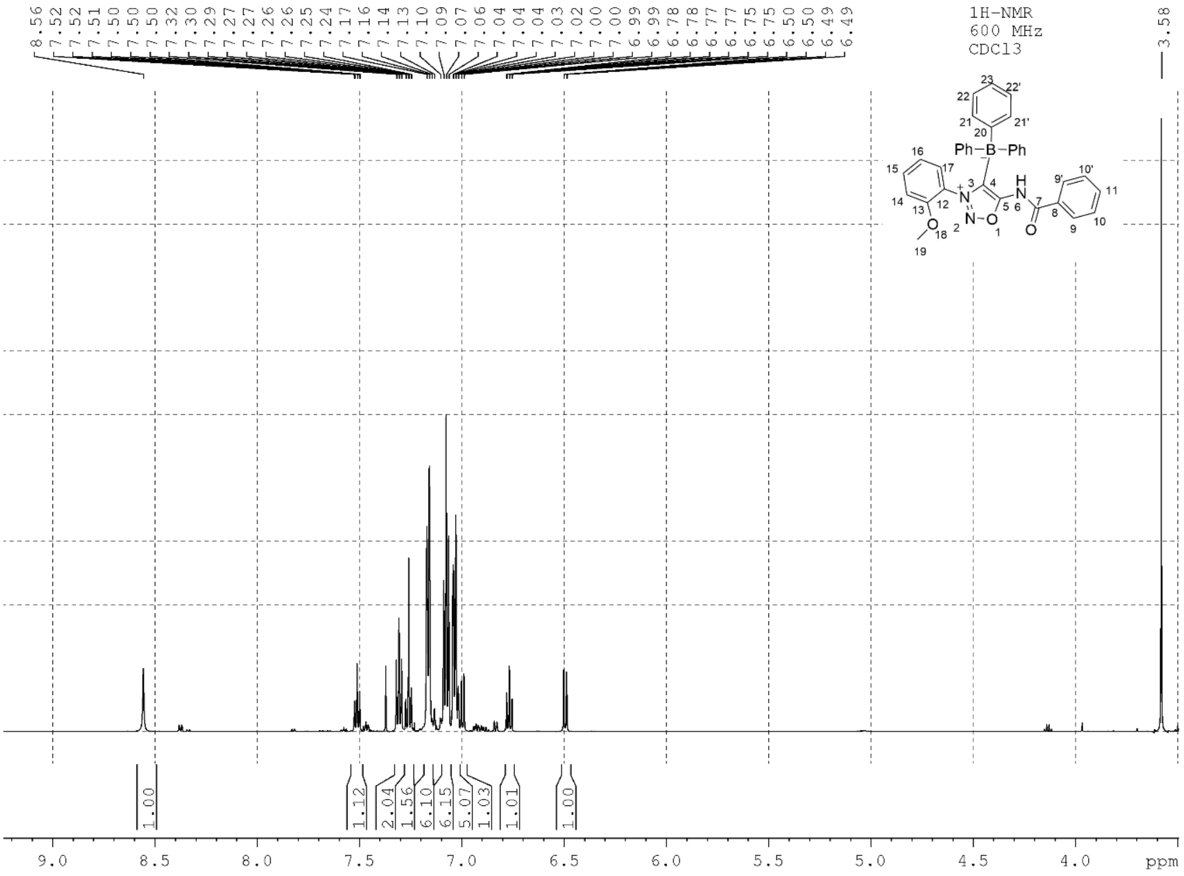
1H-NMR
600 MHz
CDCl3
8.56
3.58
1.00
1.12
2.04
1.56
6.10
6.15
5.07
1.03
1.01
1.00
ppm

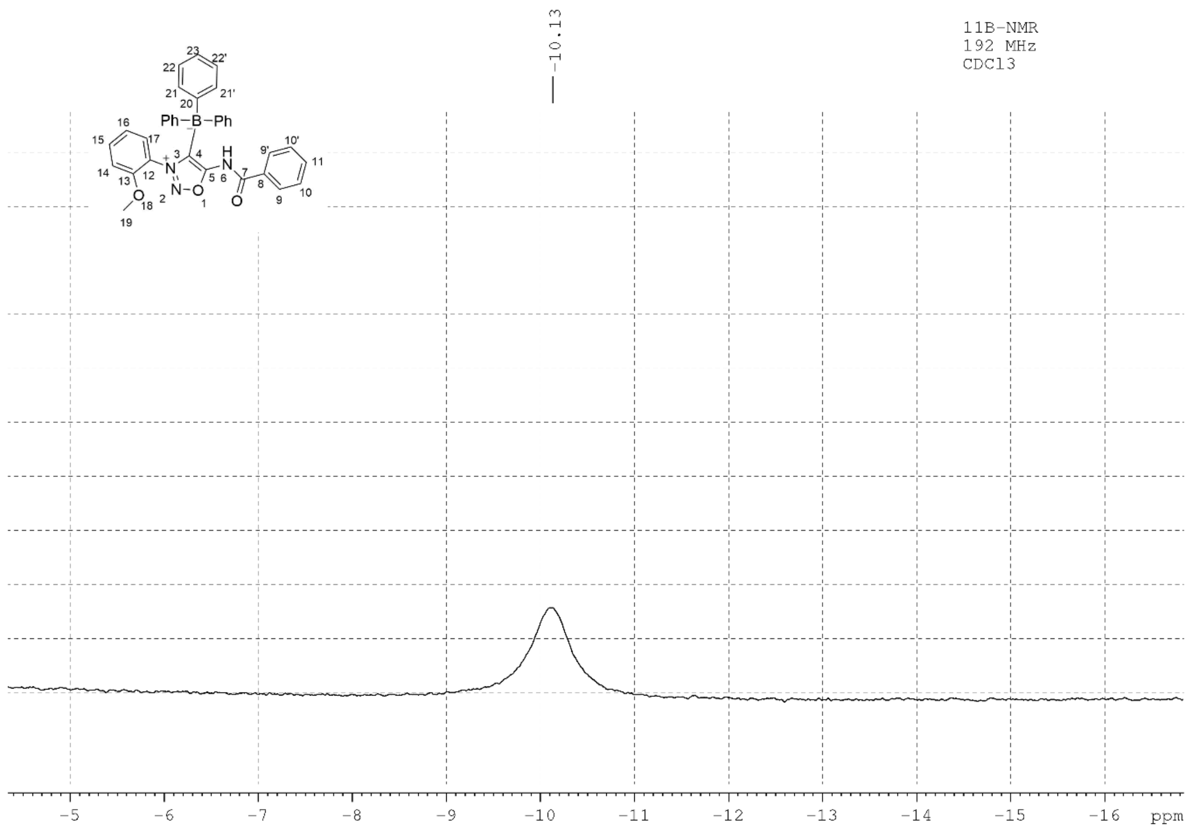
11B-NMR
192 MHz
CDCl3
-10.13
ppm

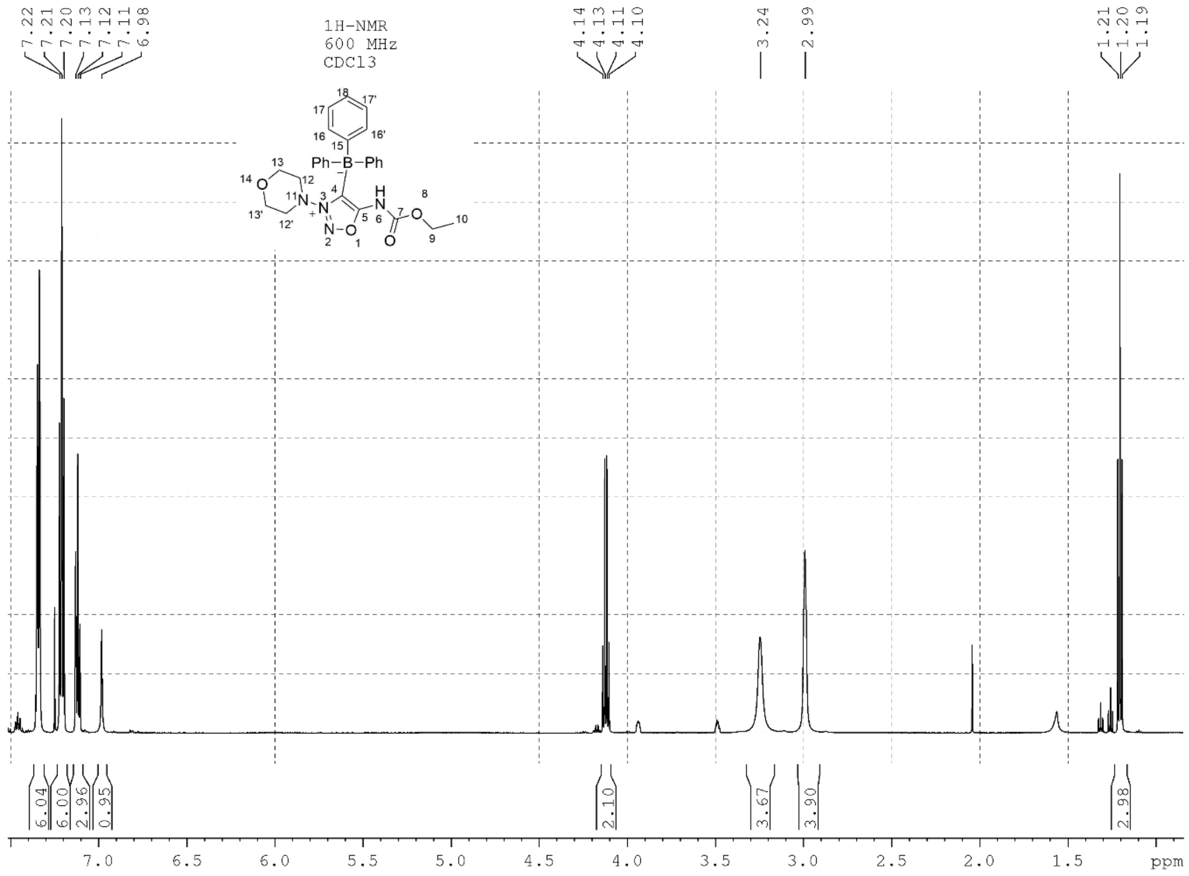
1H-NMR
600 MHz
CDCl3
7.22
7.21
7.20
7.13
7.12
7.11
6.98
4.14
4.13
4.11
4.10
3.24
2.99
1.21
1.20
1.19
6.04
6.00
2.96
0.95
2.10
3.67
3.90
2.98
ppm

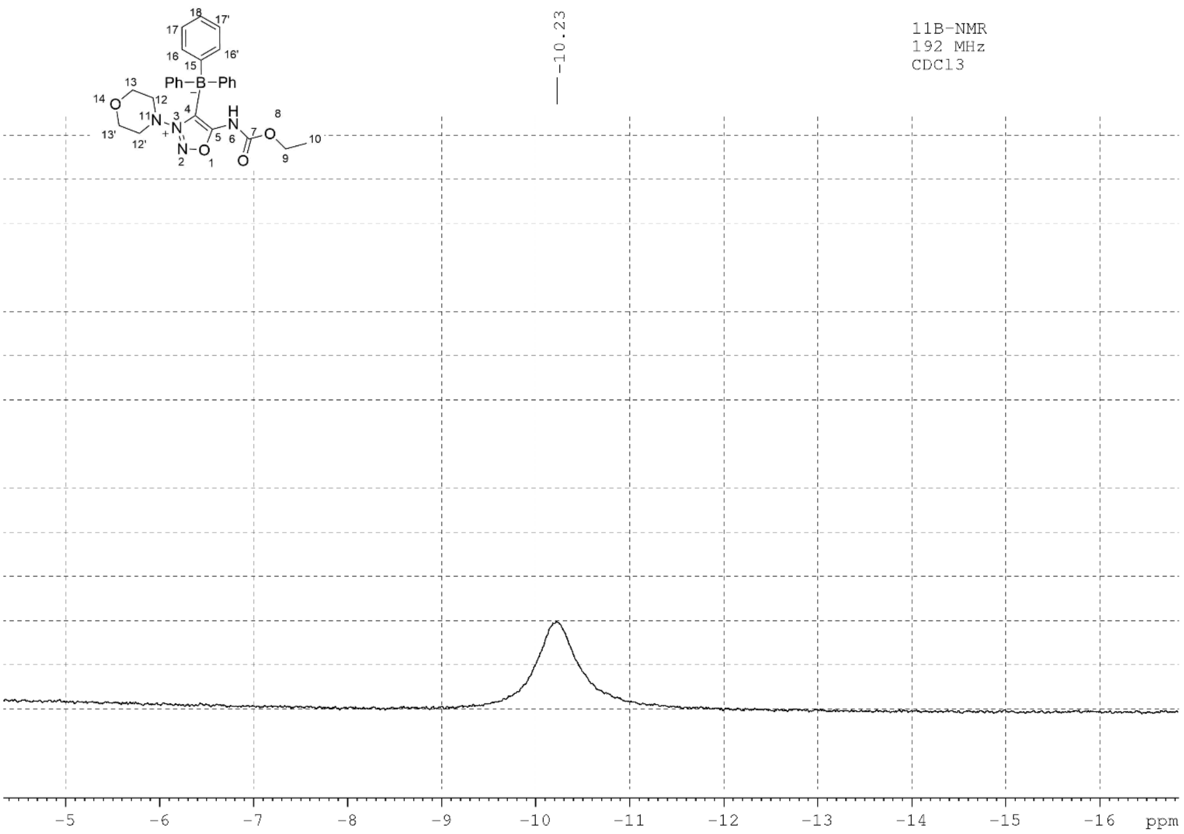
11B-NMR
192 MHz
CDCl3
-10.23
ppm

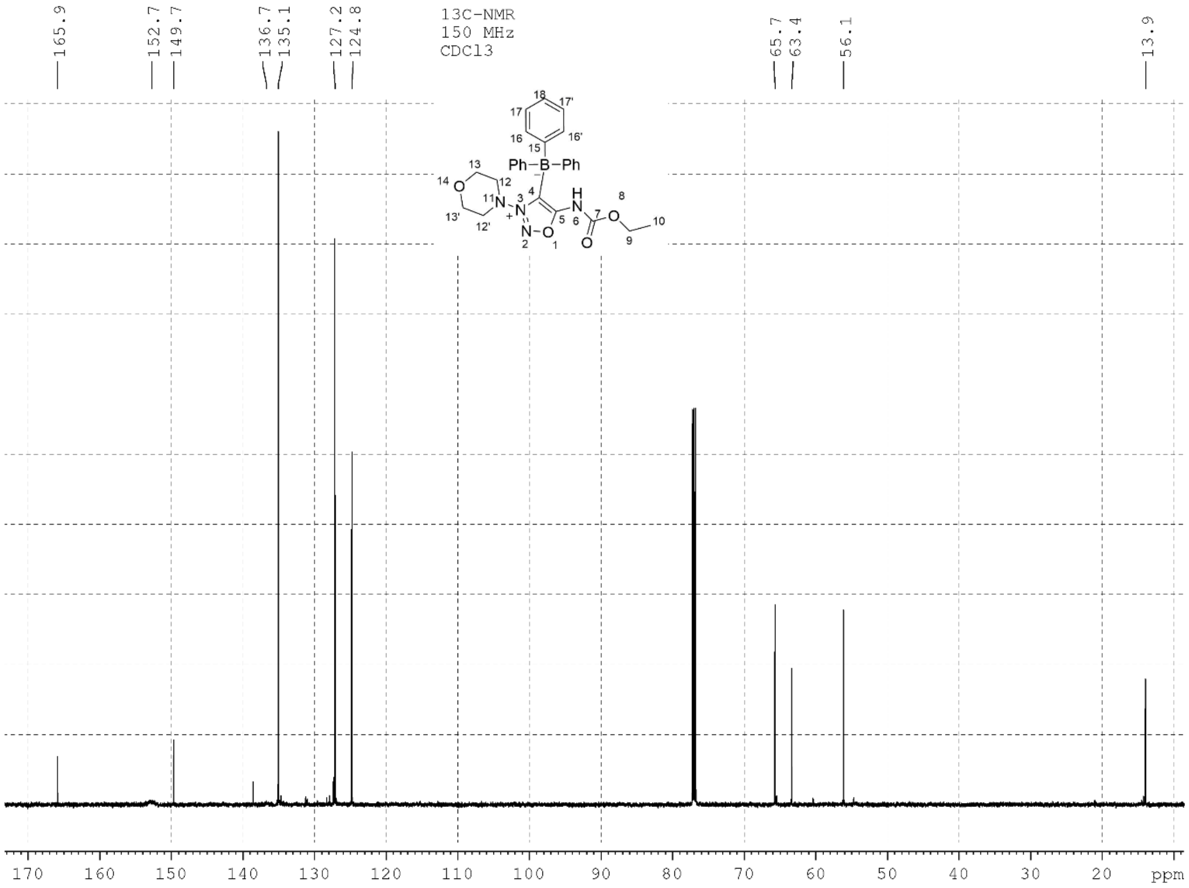
13C-NMR
150 MHz
CDCl3
165.9
152.7
149.7
136.7
135.1
127.2
124.8
65.7
63.4
56.1
13.9
ppm

2. Crystal Structure Determinations

Result of the single crystal X-ray analysis of 27a

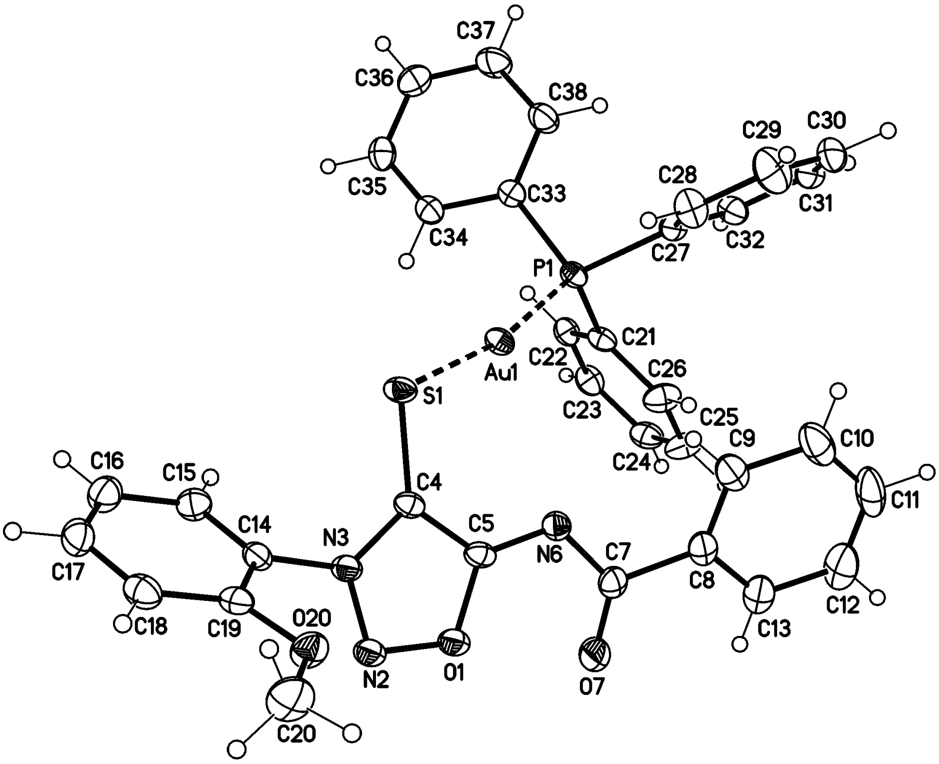

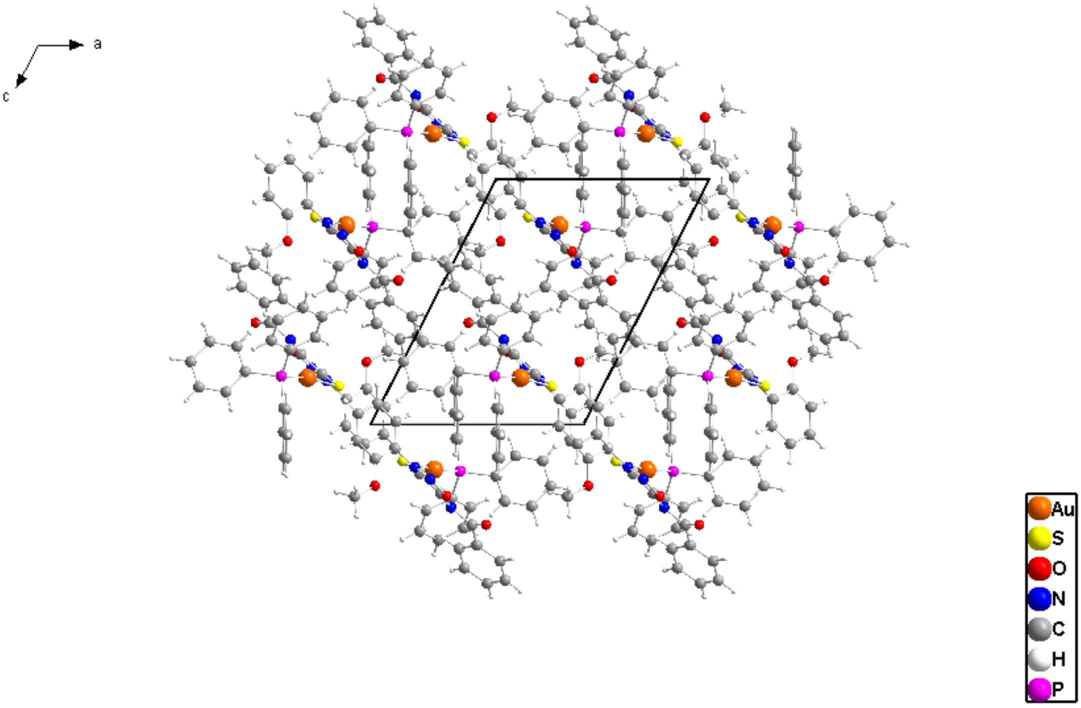

(6-(Benzoyl)-3-(2-methoxyphenyl)-sydnone imine-4-yl)thio)-(triphenylphosphin)-gold(I) - 27a

*Crystal data for **27a***

$C_{34}H_{27}AuN_3O_3PS$	$Z = 2$
$M_r = 785.58$	$F(000) = 772$
Triclinic, *P*-1 (no.2)	$D_x = 1.742$ Mg m^{-3}
$a = 11.3805$ (5) Å	Mo $K\alpha$ radiation, $\lambda = 0.71073$ Å
$b = 11.7754$ (5) Å	Cell parameters from 9992 reflections
$c = 13.5775$ (5) Å	$\theta = 2.2$–$27.5°$
$\alpha = 90.886$ (1)°	$\mu = 5.07$ mm^{-1}
$\beta = 114.586$ (1)°	$T = 123$ K
$\gamma = 112.289$ (1)°	Plates, yellow
$V = 1498.12$ (11) Å^3	0.10 × 0.05 × 0.02 mm

*Data collection for **27a***

Bruker D8 VENTURE diffractometer with PhotonII CPAD detector	6512 reflections with $I > 2\sigma(I)$
Radiation source: INCOATEC microfocus sealed tube	$R_{int} = 0.034$
rotation in ϕ and ω, 1°, shutterless scans	$\theta_{max} = 27.5°$, $\theta_{min} = 2.3°$
Absorption correction: multi-scan *SADABS* (Sheldrick, 2014)	$h = -14 \rightarrow 14$
$T_{min} = 0.711$, $T_{max} = 0.862$	$k = -15 \rightarrow 15$
43043 measured reflections	$l = -17 \rightarrow 17$
6890 independent reflections	

*Refinement for **27a***

Refinement on F^2	Primary atom site location: dual
Least-squares matrix: full	Secondary atom site location: difference Fourier map
$R[F^2 > 2\sigma(F^2)] = 0.016$	Hydrogen site location: difference Fourier map
$wR(F^2) = 0.036$	H-atom parameters constrained
$S = 1.07$	$w = 1/[\sigma^2(F_o^2) + (0.0132P)^2 + 0.9221P]$ where $P = (F_o^2 + 2F_c^2)/3$
6890 reflections	$(\Delta/\sigma)_{max} = 0.003$
389 parameters	$\Delta\rangle_{max} = 0.75$ e Å^{-3}
0 restraints	$\Delta\rangle_{min} = -0.47$ e Å^{-3}

Computing details

Data collection: *APEX3*; cell refinement: *APEX3*; data reduction: *SAINT*; program(s) used to solve structure: SHELXT; program(s) used to refine structure: *SHELXL2014*/7 (Sheldrick, 2014); software used to prepare material for publication: *publCIF*.

*Special details for **27a***

Experimental. dx = 40 mm, 1 deg., 6+1 runs, 886 frames, 20 sec./frame
Geometry. All esds (except the esd in the dihedral angle between two l.s. planes) are estimated using the full covariance matrix. The cell esds are taken into account individually in the estimation of esds in distances, angles and torsion angles; correlations between esds in cell parameters are only used when they are defined by crystal symmetry. An approximate (isotropic) treatment of cell esds is used for estimating esds involving l.s. planes.

*Fractional atomic coordinates and isotropic or equivalent isotropic displacement parameters (Å^2) for **27a***

	x	*y*	*z*	U_{iso}*/U_{eq}
Au1	0.59229 (2)	0.50837 (2)	0.81216 (2)	0.01817 (3)
S1	0.75889 (5)	0.42619 (5)	0.84664 (4)	0.02033 (10)
O1	0.47116 (16)	0.08137 (14)	0.70498 (13)	0.0247 (3)
N2	0.5870 (2)	0.05924 (17)	0.77225 (16)	0.0260 (4)
N3	0.68945 (19)	0.17328 (16)	0.82328 (14)	0.0195 (3)
C4	0.6505 (2)	0.26824 (18)	0.79574 (16)	0.0178 (4)
C5	0.5063 (2)	0.20727 (19)	0.71596 (16)	0.0192 (4)
N6	0.42260 (19)	0.25811 (16)	0.65756 (14)	0.0201 (4)
C7	0.2825 (2)	0.1839 (2)	0.58678 (17)	0.0225 (4)
O7	0.21554 (17)	0.07334 (15)	0.58645 (14)	0.0327 (4)
C8	0.2104 (3)	0.2481 (2)	0.50410 (17)	0.0243 (5)
C9	0.2865 (3)	0.3655 (2)	0.48922 (19)	0.0297 (5)
H9	0.3870	0.4074	0.5328	0.036*
C10	0.2150 (4)	0.4207 (3)	0.4107 (2)	0.0421 (7)
H10	0.2666	0.5008	0.4007	0.051*
C11	0.0686 (4)	0.3592 (3)	0.3470 (2)	0.0470 (8)
H11	0.0196	0.3978	0.2940	0.056*
C12	-0.0069 (3)	0.2418 (3)	0.3601 (2)	0.0420 (7)
H12	-0.1071	0.1992	0.3149	0.050*
C13	0.0631 (3)	0.1863 (2)	0.43854 (18)	0.0298 (5)
H13	0.0109	0.1061	0.4479	0.036*
C14	0.8275 (2)	0.18064 (19)	0.89872 (17)	0.0198 (4)
C15	0.8871 (2)	0.2341 (2)	1.00910 (18)	0.0250 (4)
H15	0.8391	0.2681	1.0349	0.030*
C16	1.0179 (3)	0.2375 (2)	1.08189 (19)	0.0289 (5)

H16	1.0607	0.2745	1.1581	0.035*
C17	1.0853 (2)	0.1869 (2)	1.04280 (19)	0.0280 (5)
H17	1.1740	0.1881	1.0931	0.034*
C18	1.0265 (2)	0.1344 (2)	0.93173 (19)	0.0264 (5)
H18	1.0751	0.1006	0.9065	0.032*
C19	0.8960 (2)	0.13147 (19)	0.85740 (18)	0.0215 (4)
O20	0.83042 (17)	0.08623 (15)	0.74676 (12)	0.0273 (3)
C20	0.9018 (3)	0.0389 (3)	0.7031 (2)	0.0371 (6)
H20A	0.9958	0.1053	0.7206	0.056*
H20B	0.8449	0.0108	0.6226	0.056*
H20C	0.9134	-0.0320	0.7361	0.056*
P1	0.46619 (6)	0.61953 (5)	0.80565 (4)	0.01714 (10)
C21	0.2855 (2)	0.52799 (18)	0.78623 (17)	0.0188 (4)
C22	0.2444 (2)	0.5315 (2)	0.86932 (17)	0.0204 (4)
H22	0.3117	0.5845	0.9399	0.024*
C23	0.1049 (2)	0.4572 (2)	0.84897 (18)	0.0241 (4)
H23	0.0775	0.4589	0.9060	0.029*
C24	0.0061 (2)	0.3812 (2)	0.74608 (19)	0.0268 (5)
H24	-0.0894	0.3313	0.7322	0.032*
C25	0.0462 (3)	0.3776 (2)	0.6631 (2)	0.0309 (5)
H25	-0.0218	0.3254	0.5923	0.037*
C26	0.1855 (3)	0.4502 (2)	0.68330 (19)	0.0277 (5)
H26	0.2129	0.4468	0.6265	0.033*
C27	0.4418 (2)	0.70648 (19)	0.69561 (16)	0.0184 (4)
C28	0.5315 (3)	0.7331 (2)	0.64597 (19)	0.0279 (5)
H28	0.6060	0.7061	0.6700	0.033*
C29	0.5117 (3)	0.7993 (3)	0.5609 (2)	0.0379 (6)
H29	0.5738	0.8184	0.5276	0.045*
C30	0.4025 (3)	0.8375 (2)	0.52449 (19)	0.0310 (5)
H30	0.3882	0.8807	0.4650	0.037*
C31	0.3144 (2)	0.8129 (2)	0.57454 (18)	0.0259 (5)
H31	0.2411	0.8413	0.5512	0.031*
C32	0.3335 (2)	0.7467 (2)	0.65905 (18)	0.0231 (4)
H32	0.2716	0.7286	0.6925	0.028*
C33	0.5541 (2)	0.73126 (19)	0.93548 (17)	0.0187 (4)
C34	0.6103 (2)	0.6912 (2)	1.03326 (17)	0.0219 (4)
H34	0.6050	0.6087	1.0300	0.026*
C35	0.6738 (2)	0.7699 (2)	1.13519 (18)	0.0251 (5)
H35	0.7098	0.7407	1.2013	0.030*
C36	0.6848 (3)	0.8911 (2)	1.14053 (19)	0.0283 (5)

H36	0.7284	0.9456	1.2102	0.034*
C37	0.6318 (3)	0.9325 (2)	1.0437 (2)	0.0340 (6)
H37	0.6407	1.0162	1.0473	0.041*
C38	0.5656 (3)	0.8532 (2)	0.94101 (19)	0.0282 (5)
H38	0.5285	0.8822	0.8750	0.034*

*Atomic displacement parameters (Å^2) for **27a***

	U^{11}	U^{22}	U^{33}	U^{12}	U^{13}	U^{23}
Au1	0.02044 (4)	0.01726 (4)	0.02239 (4)	0.01109 (3)	0.01188 (3)	0.00504 (3)
S1	0.0179 (2)	0.0147 (2)	0.0286 (3)	0.0078 (2)	0.0102 (2)	0.00194 (19)
O1	0.0223 (8)	0.0169 (7)	0.0293 (8)	0.0092 (6)	0.0061 (7)	0.0015 (6)
N2	0.0207 (9)	0.0167 (9)	0.0328 (10)	0.0087 (8)	0.0047 (8)	0.0023 (8)
N3	0.0207 (9)	0.0152 (8)	0.0220 (8)	0.0075 (7)	0.0094 (7)	0.0028 (7)
C4	0.0219 (10)	0.0156 (9)	0.0199 (9)	0.0088 (8)	0.0122 (8)	0.0035 (8)
C5	0.0251 (11)	0.0157 (10)	0.0200 (10)	0.0088 (8)	0.0131 (9)	0.0028 (8)
N6	0.0232 (9)	0.0205 (9)	0.0191 (8)	0.0118 (8)	0.0097 (7)	0.0036 (7)
C7	0.0270 (11)	0.0257 (11)	0.0175 (10)	0.0138 (10)	0.0102 (9)	0.0032 (8)
O7	0.0242 (8)	0.0272 (9)	0.0324 (9)	0.0072 (7)	0.0036 (7)	0.0078 (7)
C8	0.0340 (12)	0.0301 (12)	0.0179 (10)	0.0215 (10)	0.0128 (9)	0.0041 (9)
C9	0.0456 (15)	0.0317 (13)	0.0248 (11)	0.0239 (12)	0.0205 (11)	0.0094 (10)
C10	0.076 (2)	0.0427 (16)	0.0315 (13)	0.0408 (16)	0.0317 (15)	0.0178 (12)
C11	0.075 (2)	0.064 (2)	0.0278 (13)	0.0590 (19)	0.0188 (14)	0.0158 (13)
C12	0.0445 (16)	0.0608 (19)	0.0323 (13)	0.0421 (15)	0.0102 (12)	0.0034 (12)
C13	0.0337 (13)	0.0404 (14)	0.0237 (11)	0.0253 (11)	0.0118 (10)	0.0035 (10)
C14	0.0205 (10)	0.0162 (10)	0.0233 (10)	0.0093 (8)	0.0093 (8)	0.0062 (8)
C15	0.0268 (11)	0.0240 (11)	0.0255 (11)	0.0112 (9)	0.0127 (9)	0.0029 (9)
C16	0.0287 (12)	0.0293 (12)	0.0231 (11)	0.0099 (10)	0.0089 (10)	0.0042 (9)
C17	0.0214 (11)	0.0284 (12)	0.0291 (11)	0.0093 (10)	0.0079 (9)	0.0085 (9)
C18	0.0250 (11)	0.0232 (11)	0.0358 (12)	0.0125 (9)	0.0161 (10)	0.0077 (9)
C19	0.0226 (11)	0.0149 (10)	0.0260 (11)	0.0064 (8)	0.0116 (9)	0.0040 (8)
O20	0.0295 (9)	0.0294 (9)	0.0235 (8)	0.0141 (7)	0.0113 (7)	-0.0009 (6)
C20	0.0414 (15)	0.0421 (15)	0.0338 (13)	0.0217 (13)	0.0192 (12)	-0.0019 (11)
P1	0.0194 (3)	0.0168 (2)	0.0202 (2)	0.0100 (2)	0.0113 (2)	0.0057 (2)
C21	0.0199 (10)	0.0153 (9)	0.0259 (10)	0.0093 (8)	0.0129 (9)	0.0064 (8)
C22	0.0213 (10)	0.0224 (11)	0.0224 (10)	0.0122 (9)	0.0117 (9)	0.0077 (8)
C23	0.0258 (11)	0.0268 (11)	0.0272 (11)	0.0131 (10)	0.0167 (9)	0.0113 (9)
C24	0.0241 (11)	0.0205 (11)	0.0366 (12)	0.0069 (9)	0.0169 (10)	0.0076 (9)
C25	0.0274 (12)	0.0239 (12)	0.0323 (12)	0.0022 (10)	0.0138 (10)	-0.0060 (9)
C26	0.0310 (12)	0.0244 (11)	0.0291 (11)	0.0066 (10)	0.0195 (10)	-0.0015 (9)
C27	0.0209 (10)	0.0169 (10)	0.0180 (9)	0.0071 (8)	0.0101 (8)	0.0034 (8)

C28	0.0317 (12)	0.0358 (13)	0.0286 (11)	0.0203 (11)	0.0192 (10)	0.0116 (10)
C29	0.0511 (16)	0.0484 (16)	0.0352 (13)	0.0271 (14)	0.0324 (13)	0.0205 (12)
C30	0.0422 (14)	0.0279 (12)	0.0211 (11)	0.0146 (11)	0.0129 (10)	0.0090 (9)
C31	0.0230 (11)	0.0203 (11)	0.0270 (11)	0.0084 (9)	0.0055 (9)	0.0045 (9)
C32	0.0219 (11)	0.0205 (11)	0.0282 (11)	0.0093 (9)	0.0124 (9)	0.0074 (9)
C33	0.0189 (10)	0.0191 (10)	0.0217 (10)	0.0096 (8)	0.0112 (8)	0.0051 (8)
C34	0.0225 (11)	0.0211 (10)	0.0247 (10)	0.0109 (9)	0.0115 (9)	0.0070 (8)
C35	0.0252 (11)	0.0318 (12)	0.0212 (10)	0.0137 (10)	0.0115 (9)	0.0090 (9)
C36	0.0283 (12)	0.0294 (12)	0.0255 (11)	0.0097 (10)	0.0134 (10)	0.0003 (9)
C37	0.0476 (15)	0.0229 (12)	0.0339 (13)	0.0187 (11)	0.0174 (12)	0.0028 (10)
C38	0.0399 (14)	0.0224 (11)	0.0256 (11)	0.0173 (10)	0.0141 (10)	0.0073 (9)

*Geometric parameters (Å, º) for **27a***

Au1—P1	2.2591 (5)	C20—H20B	0.9800
Au1—S1	2.3276 (5)	C20—H20C	0.9800
Au1—N6	2.9897 (17)	P1—C27	1.810 (2)
S1—C4	1.723 (2)	P1—C33	1.817 (2)
O1—C5	1.369 (2)	P1—C21	1.821 (2)
O1—N2	1.381 (2)	C21—C26	1.390 (3)
N2—N3	1.324 (2)	C21—C22	1.395 (3)
N3—C4	1.356 (3)	C22—C23	1.391 (3)
N3—C14	1.442 (3)	C22—H22	0.9500
C4—C5	1.409 (3)	C23—C24	1.381 (3)
C5—N6	1.309 (3)	C23—H23	0.9500
N6—C7	1.368 (3)	C24—C25	1.387 (3)
C7—O7	1.233 (3)	C24—H24	0.9500
C7—C8	1.495 (3)	C25—C26	1.386 (3)
C8—C13	1.394 (3)	C25—H25	0.9500
C8—C9	1.396 (3)	C26—H26	0.9500
C9—C10	1.386 (3)	C27—C28	1.390 (3)
C9—H9	0.9500	C27—C32	1.390 (3)
C10—C11	1.384 (4)	C28—C29	1.392 (3)
C10—H10	0.9500	C28—H28	0.9500
C11—C12	1.384 (4)	C29—C30	1.382 (4)
C11—H11	0.9500	C29—H29	0.9500
C12—C13	1.380 (3)	C30—C31	1.380 (3)
C12—H12	0.9500	C30—H30	0.9500
C13—H13	0.9500	C31—C32	1.386 (3)
C14—C15	1.380 (3)	C31—H31	0.9500
C14—C19	1.398 (3)	C32—H32	0.9500

C15—C16	1.387 (3)	C33—C38	1.389 (3)
C15—H15	0.9500	C33—C34	1.391 (3)
C16—C17	1.378 (3)	C34—C35	1.384 (3)
C16—H16	0.9500	C34—H34	0.9500
C17—C18	1.386 (3)	C35—C36	1.383 (3)
C17—H17	0.9500	C35—H35	0.9500
C18—C19	1.392 (3)	C36—C37	1.383 (3)
C18—H18	0.9500	C36—H36	0.9500
C19—O20	1.356 (3)	C37—C38	1.391 (3)
O20—C20	1.434 (3)	C37—H37	0.9500
C20—H20A	0.9800	C38—H38	0.9500
P1—Au1—S1	168.824 (19)	H20A—C20—H20C	109.5
P1—Au1—N6	114.79 (4)	H20B—C20—H20C	109.5
S1—Au1—N6	76.37 (4)	C27—P1—C33	107.45 (9)
C4—S1—Au1	101.65 (7)	C27—P1—C21	103.74 (10)
C5—O1—N2	111.04 (16)	C33—P1—C21	104.93 (9)
N3—N2—O1	103.41 (15)	C27—P1—Au1	114.50 (7)
N2—N3—C4	115.11 (17)	C33—P1—Au1	110.02 (7)
N2—N3—C14	116.32 (16)	C21—P1—Au1	115.44 (7)
C4—N3—C14	128.55 (17)	C26—C21—C22	119.2 (2)
N3—C4—C5	104.07 (17)	C26—C21—P1	117.48 (16)
N3—C4—S1	126.27 (16)	C22—C21—P1	123.27 (16)
C5—C4—S1	129.64 (16)	C23—C22—C21	120.1 (2)
N6—C5—O1	125.52 (19)	C23—C22—H22	120.0
N6—C5—C4	128.02 (19)	C21—C22—H22	120.0
O1—C5—C4	106.33 (17)	C24—C23—C22	120.2 (2)
C5—N6—C7	120.01 (18)	C24—C23—H23	119.9
C5—N6—Au1	95.46 (13)	C22—C23—H23	119.9
C7—N6—Au1	137.43 (13)	C23—C24—C25	120.0 (2)
O7—C7—N6	125.9 (2)	C23—C24—H24	120.0
O7—C7—C8	120.0 (2)	C25—C24—H24	120.0
N6—C7—C8	114.11 (19)	C26—C25—C24	120.0 (2)
C13—C8—C9	119.6 (2)	C26—C25—H25	120.0
C13—C8—C7	118.4 (2)	C24—C25—H25	120.0
C9—C8—C7	122.0 (2)	C25—C26—C21	120.5 (2)
C10—C9—C8	119.9 (3)	C25—C26—H26	119.8
C10—C9—H9	120.1	C21—C26—H26	119.8
C8—C9—H9	120.1	C28—C27—C32	119.14 (19)
C11—C10—C9	120.0 (3)	C28—C27—P1	119.93 (16)

C11—C10—H10	120.0	C32—C27—P1	120.93 (15)
C9—C10—H10	120.0	C27—C28—C29	119.7 (2)
C12—C11—C10	120.3 (2)	C27—C28—H28	120.1
C12—C11—H11	119.9	C29—C28—H28	120.1
C10—C11—H11	119.9	C30—C29—C28	120.6 (2)
C13—C12—C11	120.2 (3)	C30—C29—H29	119.7
C13—C12—H12	119.9	C28—C29—H29	119.7
C11—C12—H12	119.9	C31—C30—C29	120.0 (2)
C12—C13—C8	120.0 (3)	C31—C30—H30	120.0
C12—C13—H13	120.0	C29—C30—H30	120.0
C8—C13—H13	120.0	C30—C31—C32	119.7 (2)
C15—C14—C19	122.0 (2)	C30—C31—H31	120.2
C15—C14—N3	119.46 (18)	C32—C31—H31	120.2
C19—C14—N3	118.56 (18)	C31—C32—C27	120.9 (2)
C14—C15—C16	119.2 (2)	C31—C32—H32	119.5
C14—C15—H15	120.4	C27—C32—H32	119.5
C16—C15—H15	120.4	C38—C33—C34	119.1 (2)
C17—C16—C15	119.5 (2)	C38—C33—P1	122.99 (16)
C17—C16—H16	120.3	C34—C33—P1	117.92 (16)
C15—C16—H16	120.3	C35—C34—C33	121.0 (2)
C16—C17—C18	121.5 (2)	C35—C34—H34	119.5
C16—C17—H17	119.2	C33—C34—H34	119.5
C18—C17—H17	119.2	C36—C35—C34	119.8 (2)
C17—C18—C19	119.7 (2)	C36—C35—H35	120.1
C17—C18—H18	120.2	C34—C35—H35	120.1
C19—C18—H18	120.2	C35—C36—C37	119.6 (2)
O20—C19—C18	124.97 (19)	C35—C36—H36	120.2
O20—C19—C14	116.92 (19)	C37—C36—H36	120.2
C18—C19—C14	118.1 (2)	C36—C37—C38	120.9 (2)
C19—O20—C20	117.38 (18)	C36—C37—H37	119.6
O20—C20—H20A	109.5	C38—C37—H37	119.6
O20—C20—H20B	109.5	C33—C38—C37	119.7 (2)
H20A—C20—H20B	109.5	C33—C38—H38	120.2
O20—C20—H20C	109.5	C37—C38—H38	120.2
P1—Au1—S1—C4	156.51 (11)	N3—C14—C19—O20	3.7 (3)
N6—Au1—S1—C4	-20.16 (8)	C15—C14—C19—C18	1.7 (3)
C5—O1—N2—N3	0.6 (2)	N3—C14—C19—C18	-177.43 (18)
O1—N2—N3—C4	0.4 (2)	C18—C19—O20—C20	-0.6 (3)
O1—N2—N3—C14	-178.52 (16)	C14—C19—O20—C20	178.3 (2)

N2—N3—C4—C5	-1.2 (2)	S1—Au1—P1—C27	100.74 (12)
C14—N3—C4—C5	177.58 (18)	N6—Au1—P1—C27	-82.83 (8)
N2—N3—C4—S1	-179.50 (15)	S1—Au1—P1—C33	-20.38 (13)
C14—N3—C4—S1	-0.8 (3)	N6—Au1—P1—C33	156.04 (8)
Au1—S1—C4—N3	-158.61 (16)	S1—Au1—P1—C21	-138.87 (11)
Au1—S1—C4—C5	23.47 (19)	N6—Au1—P1—C21	37.55 (8)
N2—O1—C5—N6	174.85 (19)	C27—P1—C21—C26	55.78 (18)
N2—O1—C5—C4	-1.3 (2)	C33—P1—C21—C26	168.40 (17)
N3—C4—C5—N6	-174.61 (19)	Au1—P1—C21—C26	-70.32 (18)
S1—C4—C5—N6	3.7 (3)	C27—P1—C21—C22	-125.00 (17)
N3—C4—C5—O1	1.4 (2)	C33—P1—C21—C22	-12.4 (2)
S1—C4—C5—O1	179.69 (15)	Au1—P1—C21—C22	108.90 (16)
O1—C5—N6—C7	7.6 (3)	C26—C21—C22—C23	0.3 (3)
C4—C5—N6—C7	-177.10 (19)	P1—C21—C22—C23	-178.92 (16)
O1—C5—N6—Au1	163.01 (17)	C21—C22—C23—C24	-0.9 (3)
C4—C5—N6—Au1	-21.7 (2)	C22—C23—C24—C25	0.7 (3)
P1—Au1—N6—C5	-154.40 (11)	C23—C24—C25—C26	0.1 (4)
S1—Au1—N6—C5	24.89 (11)	C24—C25—C26—C21	-0.7 (4)
P1—Au1—N6—C7	-6.6 (2)	C22—C21—C26—C25	0.5 (3)
S1—Au1—N6—C7	172.7 (2)	P1—C21—C26—C25	179.76 (19)
C5—N6—C7—O7	15.5 (3)	C33—P1—C27—C28	102.97 (19)
Au1—N6—C7—O7	-126.7 (2)	C21—P1—C27—C28	-146.25 (18)
C5—N6—C7—C8	-164.16 (18)	Au1—P1—C27—C28	-19.6 (2)
Au1—N6—C7—C8	53.6 (3)	C33—P1—C27—C32	-77.49 (19)
O7—C7—C8—C13	9.0 (3)	C21—P1—C27—C32	33.3 (2)
N6—C7—C8—C13	-171.27 (18)	Au1—P1—C27—C32	159.98 (15)
O7—C7—C8—C9	-169.4 (2)	C32—C27—C28—C29	0.0 (3)
N6—C7—C8—C9	10.4 (3)	P1—C27—C28—C29	179.60 (19)
C13—C8—C9—C10	1.0 (3)	C27—C28—C29—C30	-0.8 (4)
C7—C8—C9—C10	179.4 (2)	C28—C29—C30—C31	1.7 (4)
C8—C9—C10—C11	-0.3 (4)	C29—C30—C31—C32	-1.9 (4)
C9—C10—C11—C12	-1.0 (4)	C30—C31—C32—C27	1.1 (3)
C10—C11—C12—C13	1.4 (4)	C28—C27—C32—C31	-0.2 (3)
C11—C12—C13—C8	-0.7 (4)	P1—C27—C32—C31	-179.78 (17)
C9—C8—C13—C12	-0.5 (3)	C27—P1—C33—C38	13.7 (2)
C7—C8—C13—C12	-179.0 (2)	C21—P1—C33—C38	-96.3 (2)
N2—N3—C14—C15	-119.0 (2)	Au1—P1—C33—C38	138.97 (18)
C4—N3—C14—C15	62.3 (3)	C27—P1—C33—C34	-167.17 (16)
N2—N3—C14—C19	60.1 (3)	C21—P1—C33—C34	82.87 (18)
C4—N3—C14—C19	-118.6 (2)	Au1—P1—C33—C34	-41.91 (18)

C19—C14—C15—C16	-1.0 (3)	C38—C33—C34—C35	1.6 (3)
N3—C14—C15—C16	178.1 (2)	P1—C33—C34—C35	-177.52 (16)
C14—C15—C16—C17	-0.4 (3)	C33—C34—C35—C36	-1.4 (3)
C15—C16—C17—C18	1.2 (4)	C34—C35—C36—C37	0.1 (3)
C16—C17—C18—C19	-0.5 (3)	C35—C36—C37—C38	1.0 (4)
C17—C18—C19—O20	177.9 (2)	C34—C33—C38—C37	-0.5 (3)
C17—C18—C19—C14	-1.0 (3)	P1—C33—C38—C37	178.60 (19)
C15—C14—C19—O20	-177.20 (19)	C36—C37—C38—C33	-0.8 (4)

*Hydrogen-bond geometry (Å, º) for **27a***

D—H···*A*	*D*—H	H···*A*	*D*···*A*	*D*—H···*A*
C38—H38···N2^{i}	0.95	2.56	3.374 (3)	144

Symmetry code: (i) *x*, *y*+1, *z*.

Result of the single crystal X-ray analysis of 28a

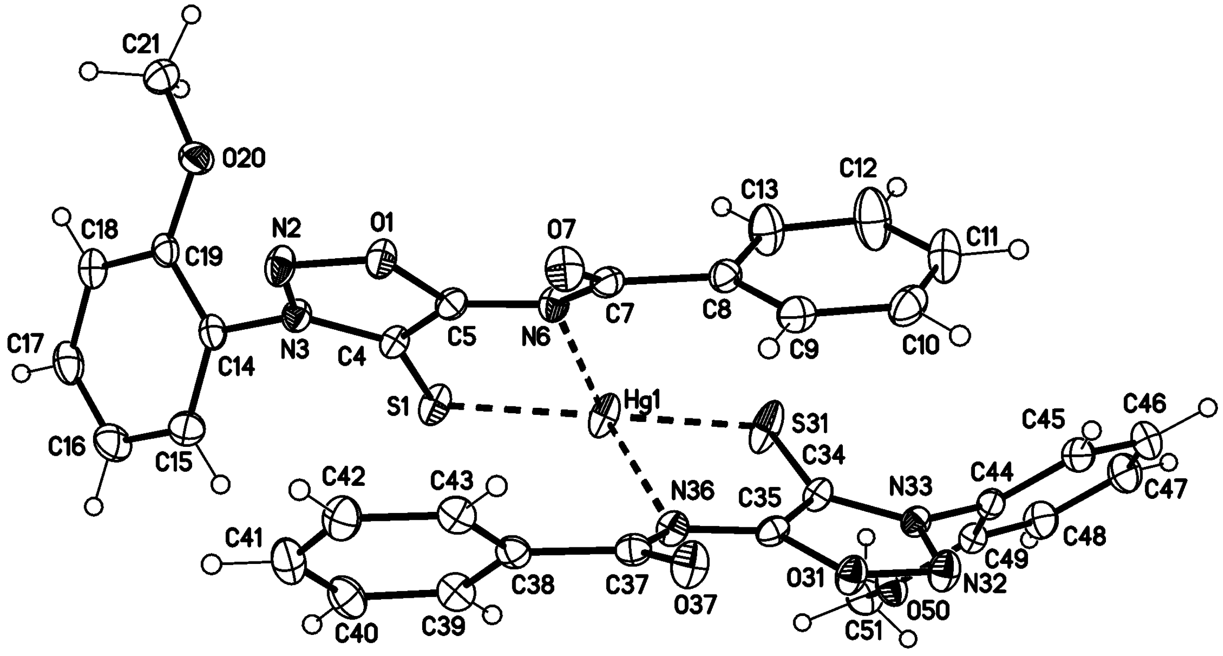

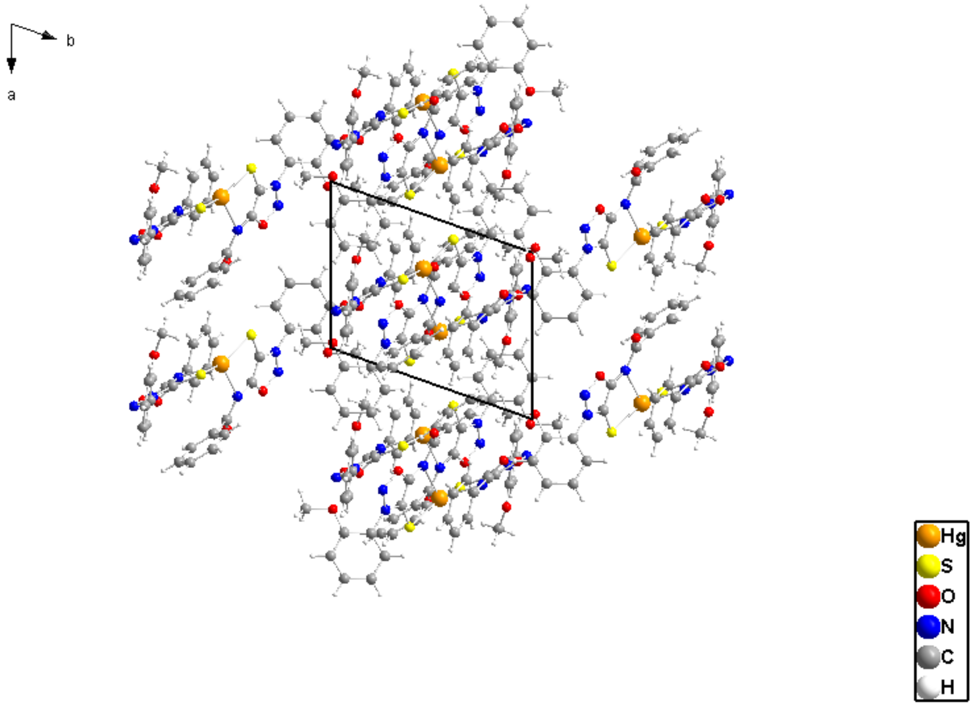

Bis((6-(benzoyl)-3-(2-methoxyphenyl)-sydnone imine-4-yl)thio)mercury(II) - 28a

Crystal data for **28a**

$C_{32}H_{24}HgN_6O_6S_2$	$Z = 2$
$M_r = 853.28$	$F(000) = 836$
Triclinic, *P*-1 (no.2)	$D_x = 1.819$ Mg m^{-3}
$a = 8.5954$ (11) Å	Mo $K\alpha$ radiation, $\lambda = 0.71073$ Å
$b = 11.3147$ (14) Å	Cell parameters from 9918 reflections
$c = 17.4308$ (19) Å	$\theta = 2.3$–27.5°
$\alpha = 77.613$ (4)°	$\mu = 5.13$ mm^{-1}
$\beta = 86.366$ (4)°	$T = 123$ K
$\gamma = 70.249$ (4)°	Blocks, yellow
$V = 1558.3$ (3) Å^3	0.16 × 0.10 × 0.04 mm

Data collection for **28a**

Bruker D8 VENTURE diffractometer with PhotonII CPAD detector	6733 reflections with $I > 2\sigma(I)$
Radiation source: INCOATEC microfocus sealed tube	$R_{int} = 0.031$
rotation in ϕ and ω, 1°, shutterless scans	$\theta_{max} = 27.5°$, $\theta_{min} = 2.4°$
Absorption correction: multi-scan	$h = -11 \rightarrow 11$

SADABS (Sheldrick, 2014)	
T_{min} = 0.607, T_{max} = 0.746	k = -14→14
38298 measured reflections	l = -22→22
7157 independent reflections	

Refinement for **28a**

Refinement on F^2	Secondary atom site location: difference Fourier map
Least-squares matrix: full	Hydrogen site location: difference Fourier map
$R[F^2 > 2\sigma(F^2)]$ = 0.018	H-atom parameters constrained
$wR(F^2)$ = 0.037	$w = 1/[\sigma^2(F_o^2) + (0.0094P)^2 + 1.3275P]$ where $P = (F_o^2 + 2F_c^2)/3$
S = 1.08	$(\Delta/\sigma)_{max}$ = 0.005
7157 reflections	$\Delta\rangle_{max}$ = 0.71 e Å^{-3}
427 parameters	$\Delta\rangle_{min}$ = -0.67 e Å^{-3}
0 restraints	Extinction correction: *SHELXL2014*/7 (Sheldrick 2014), $Fc^*=kFc[1+0.001xFc^2\lambda^3/\sin(2\theta)]^{-1/4}$
Primary atom site location: dual	Extinction coefficient: 0.00111 (14)

Computing details

Data collection: *APEX3*; cell refinement: *APEX3*; data reduction: *SAINT*; program(s) used to solve structure: SHELXT; program(s) used to refine structure: *SHELXL2014*/7 (Sheldrick, 2014); software used to prepare material for publication: *publCIF*.

Special details for **28a**

Experimental. dx = 40 mm, 1 deg., 5+1 runs, 690 frames, 5 sec./frame
Geometry. All esds (except the esd in the dihedral angle between two l.s. planes) are estimated using the full covariance matrix. The cell esds are taken into account individually in the estimation of esds in distances, angles and torsion angles; correlations between esds in cell parameters are only used when they are defined by crystal symmetry. An approximate (isotropic) treatment of cell esds is used for estimating esds involving l.s. planes.

Fractional atomic coordinates and isotropic or equivalent isotropic displacement parameters (Å²) for **28a**

	x	y	z	$U_{iso}*/U_{eq}$
Hg1	0.67183 (2)	0.54114 (2)	0.64410 (2)	0.02331 (4)
S1	0.91000 (6)	0.39202 (5)	0.71842 (3)	0.02259 (11)
O1	0.59612 (17)	0.33488 (14)	0.88166 (8)	0.0200 (3)
N2	0.7436 (2)	0.25937 (17)	0.92062 (10)	0.0212 (4)

N3	0.8571 (2)	0.27247 (16)	0.86749 (10)	0.0172 (3)
C4	0.7967 (2)	0.35090 (19)	0.79793 (11)	0.0169 (4)
C5	0.6245 (2)	0.38943 (19)	0.80713 (11)	0.0164 (4)
N6	0.5116 (2)	0.45893 (16)	0.75243 (10)	0.0180 (3)
C7	0.3472 (2)	0.51305 (18)	0.77106 (12)	0.0172 (4)
O7	0.29345 (18)	0.51209 (15)	0.83783 (8)	0.0232 (3)
C8	0.2355 (2)	0.57735 (19)	0.70096 (12)	0.0175 (4)
C9	0.1032 (2)	0.6892 (2)	0.70385 (13)	0.0207 (4)
H9	0.0879	0.7253	0.7494	0.025*
C10	-0.0058 (3)	0.7482 (2)	0.64115 (14)	0.0256 (5)
H10	-0.0930	0.8260	0.6431	0.031*
C11	0.0117 (3)	0.6944 (2)	0.57570 (14)	0.0311 (5)
H11	-0.0644	0.7340	0.5330	0.037*
C12	0.1414 (3)	0.5821 (3)	0.57292 (15)	0.0389 (6)
H12	0.1531	0.5439	0.5284	0.047*
C13	0.2542 (3)	0.5249 (2)	0.63463 (13)	0.0289 (5)
H13	0.3446	0.4494	0.6314	0.035*
C14	1.0288 (2)	0.2017 (2)	0.88696 (11)	0.0177 (4)
C15	1.1358 (3)	0.2692 (2)	0.88735 (13)	0.0243 (4)
H15	1.0960	0.3604	0.8771	0.029*
C16	1.3023 (3)	0.2020 (2)	0.90297 (14)	0.0289 (5)
H16	1.3777	0.2466	0.9044	0.035*
C17	1.3573 (3)	0.0695 (2)	0.91643 (13)	0.0261 (5)
H17	1.4716	0.0237	0.9261	0.031*
C18	1.2499 (3)	0.0019 (2)	0.91616 (12)	0.0224 (4)
H18	1.2905	-0.0892	0.9258	0.027*
C19	1.0821 (2)	0.0680 (2)	0.90167 (11)	0.0175 (4)
O20	0.96520 (18)	0.01249 (14)	0.90073 (9)	0.0224 (3)
C21	1.0203 (3)	-0.1232 (2)	0.90237 (13)	0.0243 (4)
H21A	1.0933	-0.1418	0.8578	0.036*
H21B	0.9245	-0.1503	0.8989	0.036*
H21C	1.0807	-0.1700	0.9515	0.036*
S31	0.56410 (8)	0.64108 (5)	0.51422 (3)	0.03154 (14)
O31	0.28538 (17)	0.92852 (13)	0.60275 (8)	0.0198 (3)
N32	0.2328 (2)	0.97167 (17)	0.52487 (10)	0.0217 (4)
N33	0.3280 (2)	0.87866 (15)	0.49031 (10)	0.0158 (3)
C34	0.4365 (2)	0.78049 (19)	0.53884 (11)	0.0171 (4)
C35	0.4121 (2)	0.81524 (18)	0.61211 (12)	0.0158 (4)
N36	0.4999 (2)	0.75212 (16)	0.67691 (10)	0.0178 (3)
C37	0.4453 (2)	0.78093 (19)	0.74910 (12)	0.0180 (4)

O37	0.30956 (18)	0.85713 (14)	0.76061 (8)	0.0236 (3)
C38	0.5648 (2)	0.70838 (19)	0.81545 (12)	0.0176 (4)
C39	0.7347 (3)	0.6793 (2)	0.80512 (13)	0.0219 (4)
H39	0.7765	0.7030	0.7545	0.026*
C40	0.8437 (3)	0.6158 (2)	0.86798 (13)	0.0262 (5)
H40	0.9593	0.5974	0.8606	0.031*
C41	0.7827 (3)	0.5795 (2)	0.94152 (13)	0.0269 (5)
H41	0.8567	0.5357	0.9846	0.032*
C42	0.6138 (3)	0.6070 (2)	0.95244 (13)	0.0243 (5)
H42	0.5725	0.5810	1.0028	0.029*
C43	0.5055 (3)	0.6724 (2)	0.88995 (12)	0.0198 (4)
H43	0.3898	0.6929	0.8979	0.024*
C44	0.3037 (2)	0.89203 (18)	0.40763 (11)	0.0168 (4)
C45	0.1479 (3)	0.9129 (2)	0.37956 (13)	0.0223 (4)
H45	0.0576	0.9175	0.4145	0.027*
C46	0.1251 (3)	0.9269 (2)	0.29962 (14)	0.0280 (5)
H46	0.0184	0.9425	0.2792	0.034*
C47	0.2582 (3)	0.9180 (2)	0.24977 (13)	0.0291 (5)
H47	0.2424	0.9263	0.1952	0.035*
C48	0.4147 (3)	0.8973 (2)	0.27797 (12)	0.0246 (5)
H48	0.5049	0.8910	0.2428	0.029*
C49	0.4393 (2)	0.88584 (19)	0.35772 (12)	0.0179 (4)
O50	0.58381 (17)	0.87183 (15)	0.39094 (8)	0.0225 (3)
C51	0.7302 (3)	0.8324 (2)	0.34526 (14)	0.0252 (5)
H51A	0.7331	0.7569	0.3255	0.038*
H51B	0.8281	0.8110	0.3782	0.038*
H51C	0.7294	0.9024	0.3009	0.038*

Atomic displacement parameters (Å^2) for **28a**

	U^{11}	U^{22}	U^{33}	U^{12}	U^{13}	U^{23}
Hg1	0.02347 (5)	0.02021 (5)	0.01601 (5)	0.00244 (3)	0.00284 (3)	0.00108 (3)
S1	0.0161 (2)	0.0235 (3)	0.0201 (2)	-0.0012 (2)	0.00492 (19)	0.0024 (2)
O1	0.0146 (7)	0.0230 (7)	0.0176 (7)	-0.0044 (6)	0.0001 (5)	0.0024 (6)
N2	0.0140 (8)	0.0252 (9)	0.0188 (8)	-0.0039 (7)	-0.0004 (7)	0.0031 (7)
N3	0.0152 (8)	0.0184 (8)	0.0161 (8)	-0.0052 (7)	0.0000 (6)	-0.0005 (7)
C4	0.0171 (9)	0.0151 (9)	0.0159 (9)	-0.0036 (7)	0.0002 (7)	-0.0006 (7)
C5	0.0176 (9)	0.0165 (9)	0.0146 (9)	-0.0060 (8)	0.0022 (7)	-0.0021 (7)
N6	0.0157 (8)	0.0181 (8)	0.0174 (8)	-0.0031 (7)	-0.0003 (6)	-0.0016 (7)
C7	0.0169 (9)	0.0138 (9)	0.0205 (10)	-0.0049 (7)	0.0007 (8)	-0.0029 (8)
O7	0.0186 (7)	0.0300 (8)	0.0190 (7)	-0.0065 (6)	0.0038 (6)	-0.0045 (6)

C8	0.0158 (9)	0.0167 (9)	0.0186 (9)	-0.0048 (8)	0.0006 (7)	-0.0021 (8)
C9	0.0169 (10)	0.0181 (10)	0.0262 (11)	-0.0042 (8)	0.0034 (8)	-0.0064 (8)
C10	0.0189 (10)	0.0181 (10)	0.0336 (12)	-0.0019 (8)	0.0019 (9)	0.0006 (9)
C11	0.0210 (11)	0.0383 (14)	0.0240 (11)	-0.0010 (10)	-0.0037 (9)	0.0009 (10)
C12	0.0315 (13)	0.0507 (16)	0.0249 (12)	0.0044 (12)	-0.0058 (10)	-0.0151 (12)
C13	0.0227 (11)	0.0312 (12)	0.0259 (11)	0.0038 (9)	-0.0025 (9)	-0.0113 (10)
C14	0.0137 (9)	0.0221 (10)	0.0155 (9)	-0.0045 (8)	-0.0019 (7)	-0.0017 (8)
C15	0.0227 (11)	0.0254 (11)	0.0259 (11)	-0.0108 (9)	-0.0001 (9)	-0.0027 (9)
C16	0.0213 (11)	0.0379 (13)	0.0310 (12)	-0.0167 (10)	-0.0018 (9)	-0.0027 (10)
C17	0.0151 (10)	0.0372 (13)	0.0224 (11)	-0.0058 (9)	-0.0026 (8)	-0.0025 (10)
C18	0.0192 (10)	0.0254 (11)	0.0182 (10)	-0.0027 (8)	-0.0031 (8)	-0.0024 (8)
C19	0.0176 (9)	0.0225 (10)	0.0123 (9)	-0.0070 (8)	-0.0011 (7)	-0.0020 (8)
O20	0.0187 (7)	0.0198 (7)	0.0293 (8)	-0.0065 (6)	-0.0037 (6)	-0.0050 (6)
C21	0.0271 (11)	0.0194 (10)	0.0255 (11)	-0.0070 (9)	-0.0016 (9)	-0.0034 (9)
S31	0.0430 (3)	0.0210 (3)	0.0143 (2)	0.0109 (2)	0.0004 (2)	-0.0046 (2)
O31	0.0213 (7)	0.0172 (7)	0.0166 (7)	0.0000 (6)	-0.0007 (6)	-0.0044 (6)
N32	0.0222 (9)	0.0204 (9)	0.0177 (8)	0.0000 (7)	-0.0008 (7)	-0.0051 (7)
N33	0.0143 (8)	0.0145 (8)	0.0171 (8)	-0.0033 (6)	0.0014 (6)	-0.0030 (6)
C34	0.0175 (9)	0.0144 (9)	0.0160 (9)	-0.0021 (8)	0.0011 (7)	-0.0016 (7)
C35	0.0142 (9)	0.0119 (9)	0.0196 (9)	-0.0024 (7)	0.0037 (7)	-0.0039 (7)
N36	0.0183 (8)	0.0180 (8)	0.0151 (8)	-0.0042 (7)	0.0020 (6)	-0.0030 (7)
C37	0.0196 (10)	0.0177 (10)	0.0178 (9)	-0.0078 (8)	0.0027 (8)	-0.0044 (8)
O37	0.0208 (7)	0.0246 (8)	0.0197 (7)	0.0002 (6)	0.0033 (6)	-0.0059 (6)
C38	0.0189 (10)	0.0168 (9)	0.0181 (9)	-0.0053 (8)	0.0005 (8)	-0.0072 (8)
C39	0.0204 (10)	0.0253 (11)	0.0206 (10)	-0.0078 (9)	0.0038 (8)	-0.0069 (9)
C40	0.0175 (10)	0.0330 (12)	0.0292 (12)	-0.0069 (9)	-0.0013 (9)	-0.0106 (10)
C41	0.0253 (11)	0.0302 (12)	0.0228 (11)	-0.0041 (9)	-0.0061 (9)	-0.0073 (9)
C42	0.0272 (11)	0.0270 (11)	0.0174 (10)	-0.0072 (9)	0.0018 (8)	-0.0050 (9)
C43	0.0192 (10)	0.0206 (10)	0.0200 (10)	-0.0057 (8)	0.0026 (8)	-0.0074 (8)
C44	0.0180 (9)	0.0150 (9)	0.0158 (9)	-0.0039 (8)	-0.0015 (7)	-0.0019 (7)
C45	0.0191 (10)	0.0200 (10)	0.0269 (11)	-0.0056 (8)	-0.0015 (8)	-0.0039 (9)
C46	0.0253 (11)	0.0288 (12)	0.0296 (12)	-0.0084 (9)	-0.0107 (9)	-0.0032 (10)
C47	0.0361 (13)	0.0321 (12)	0.0199 (10)	-0.0116 (10)	-0.0081 (9)	-0.0040 (9)
C48	0.0281 (11)	0.0285 (11)	0.0165 (10)	-0.0096 (9)	0.0015 (8)	-0.0033 (9)
C49	0.0182 (10)	0.0165 (9)	0.0176 (9)	-0.0050 (8)	-0.0015 (8)	-0.0015 (8)
O50	0.0159 (7)	0.0322 (8)	0.0195 (7)	-0.0093 (6)	0.0012 (6)	-0.0035 (6)
C51	0.0171 (10)	0.0265 (11)	0.0315 (12)	-0.0065 (9)	0.0062 (9)	-0.0075 (9)

Geometric parameters (Å, º) for **28a**

Hg1—S31	2.3919 (6)	C21—H21B	0.9800

Hg1—S1	2.4042 (6)	C21—H21C	0.9800
Hg1—N6	2.4717 (17)	S31—C34	1.714 (2)
Hg1—N36	2.5107 (17)	O31—C35	1.359 (2)
S1—C4	1.716 (2)	O31—N32	1.389 (2)
O1—C5	1.359 (2)	N32—N33	1.329 (2)
O1—N2	1.388 (2)	N33—C34	1.348 (2)
N2—N3	1.325 (2)	N33—C44	1.438 (2)
N3—C4	1.354 (3)	C34—C35	1.398 (3)
N3—C14	1.443 (2)	C35—N36	1.324 (3)
C4—C5	1.404 (3)	N36—C37	1.381 (2)
C5—N6	1.322 (3)	C37—O37	1.228 (2)
N6—C7	1.385 (2)	C37—C38	1.497 (3)
C7—O7	1.224 (2)	C38—C39	1.393 (3)
C7—C8	1.497 (3)	C38—C43	1.393 (3)
C8—C13	1.386 (3)	C39—C40	1.390 (3)
C8—C9	1.394 (3)	C39—H39	0.9500
C9—C10	1.383 (3)	C40—C41	1.386 (3)
C9—H9	0.9500	C40—H40	0.9500
C10—C11	1.382 (3)	C41—C42	1.388 (3)
C10—H10	0.9500	C41—H41	0.9500
C11—C12	1.386 (3)	C42—C43	1.385 (3)
C11—H11	0.9500	C42—H42	0.9500
C12—C13	1.388 (3)	C43—H43	0.9500
C12—H12	0.9500	C44—C45	1.381 (3)
C13—H13	0.9500	C44—C49	1.401 (3)
C14—C15	1.381 (3)	C45—C46	1.387 (3)
C14—C19	1.395 (3)	C45—H45	0.9500
C15—C16	1.388 (3)	C46—C47	1.381 (3)
C15—H15	0.9500	C46—H46	0.9500
C16—C17	1.382 (3)	C47—C48	1.388 (3)
C16—H16	0.9500	C47—H47	0.9500
C17—C18	1.385 (3)	C48—C49	1.391 (3)
C17—H17	0.9500	C48—H48	0.9500
C18—C19	1.393 (3)	C49—O50	1.350 (2)
C18—H18	0.9500	O50—C51	1.436 (2)
C19—O20	1.355 (2)	C51—H51A	0.9800
O20—C21	1.440 (2)	C51—H51B	0.9800
C21—H21A	0.9800	C51—H51C	0.9800
S31—Hg1—S1	144.170 (19)	O20—C21—H21C	109.5

S31—Hg1—N6	123.82 (4)	H21A—C21—H21C	109.5
S1—Hg1—N6	84.90 (4)	H21B—C21—H21C	109.5
S31—Hg1—N36	84.09 (4)	C34—S31—Hg1	95.39 (7)
S1—Hg1—N36	124.38 (4)	C35—O31—N32	110.86 (14)
N6—Hg1—N36	81.91 (5)	N33—N32—O31	103.16 (15)
C4—S1—Hg1	94.02 (7)	N32—N33—C34	114.55 (16)
C5—O1—N2	110.87 (14)	N32—N33—C44	117.86 (16)
N3—N2—O1	103.26 (15)	C34—N33—C44	127.59 (16)
N2—N3—C4	114.91 (16)	N33—C34—C35	104.83 (16)
N2—N3—C14	118.79 (16)	N33—C34—S31	125.85 (15)
C4—N3—C14	126.28 (17)	C35—C34—S31	129.20 (15)
N3—C4—C5	104.18 (17)	N36—C35—O31	126.99 (17)
N3—C4—S1	126.57 (15)	N36—C35—C34	126.46 (17)
C5—C4—S1	129.24 (15)	O31—C35—C34	106.50 (16)
N6—C5—O1	126.52 (18)	C35—N36—C37	121.05 (17)
N6—C5—C4	126.62 (18)	C35—N36—Hg1	103.85 (12)
O1—C5—C4	106.74 (17)	C37—N36—Hg1	129.37 (13)
C5—N6—C7	121.66 (17)	O37—C37—N36	124.61 (19)
C5—N6—Hg1	103.82 (12)	O37—C37—C38	120.94 (18)
C7—N6—Hg1	128.55 (13)	N36—C37—C38	114.44 (17)
O7—C7—N6	125.00 (19)	C39—C38—C43	118.92 (19)
O7—C7—C8	121.10 (18)	C39—C38—C37	121.46 (18)
N6—C7—C8	113.89 (17)	C43—C38—C37	119.59 (18)
C13—C8—C9	118.84 (19)	C40—C39—C38	120.7 (2)
C13—C8—C7	121.75 (18)	C40—C39—H39	119.6
C9—C8—C7	119.31 (18)	C38—C39—H39	119.6
C10—C9—C8	120.7 (2)	C41—C40—C39	119.6 (2)
C10—C9—H9	119.7	C41—C40—H40	120.2
C8—C9—H9	119.7	C39—C40—H40	120.2
C11—C10—C9	120.3 (2)	C40—C41—C42	120.2 (2)
C11—C10—H10	119.9	C40—C41—H41	119.9
C9—C10—H10	119.9	C42—C41—H41	119.9
C10—C11—C12	119.3 (2)	C43—C42—C41	120.0 (2)
C10—C11—H11	120.3	C43—C42—H42	120.0
C12—C11—H11	120.3	C41—C42—H42	120.0
C11—C12—C13	120.5 (2)	C42—C43—C38	120.5 (2)
C11—C12—H12	119.7	C42—C43—H43	119.7
C13—C12—H12	119.7	C38—C43—H43	119.7
C8—C13—C12	120.3 (2)	C45—C44—C49	122.02 (19)
C8—C13—H13	119.9	C45—C44—N33	119.44 (18)

C12—C13—H13	119.9	C49—C44—N33	118.53 (17)
C15—C14—C19	122.34 (19)	C44—C45—C46	119.1 (2)
C15—C14—N3	118.66 (18)	C44—C45—H45	120.5
C19—C14—N3	118.95 (18)	C46—C45—H45	120.5
C14—C15—C16	119.1 (2)	C47—C46—C45	119.7 (2)
C14—C15—H15	120.5	C47—C46—H46	120.2
C16—C15—H15	120.5	C45—C46—H46	120.2
C17—C16—C15	119.2 (2)	C46—C47—C48	121.2 (2)
C17—C16—H16	120.4	C46—C47—H47	119.4
C15—C16—H16	120.4	C48—C47—H47	119.4
C16—C17—C18	121.7 (2)	C47—C48—C49	119.9 (2)
C16—C17—H17	119.2	C47—C48—H48	120.0
C18—C17—H17	119.2	C49—C48—H48	120.0
C17—C18—C19	119.7 (2)	O50—C49—C48	124.97 (19)
C17—C18—H18	120.1	O50—C49—C44	117.01 (17)
C19—C18—H18	120.1	C48—C49—C44	118.01 (19)
O20—C19—C18	125.10 (19)	C49—O50—C51	116.78 (16)
O20—C19—C14	116.95 (17)	O50—C51—H51A	109.5
C18—C19—C14	117.95 (19)	O50—C51—H51B	109.5
C19—O20—C21	117.71 (16)	H51A—C51—H51B	109.5
O20—C21—H21A	109.5	O50—C51—H51C	109.5
O20—C21—H21B	109.5	H51A—C51—H51C	109.5
H21A—C21—H21B	109.5	H51B—C51—H51C	109.5
S31—Hg1—S1—C4	-152.98 (7)	S1—Hg1—S31—C34	-149.63 (7)
N6—Hg1—S1—C4	-6.78 (8)	N6—Hg1—S31—C34	72.21 (9)
N36—Hg1—S1—C4	69.78 (9)	N36—Hg1—S31—C34	-3.91 (8)
C5—O1—N2—N3	0.6 (2)	C35—O31—N32—N33	2.1 (2)
O1—N2—N3—C4	0.8 (2)	O31—N32—N33—C34	-0.2 (2)
O1—N2—N3—C14	-177.59 (16)	O31—N32—N33—C44	179.03 (16)
N2—N3—C4—C5	-1.8 (2)	N32—N33—C34—C35	-1.8 (2)
C14—N3—C4—C5	176.48 (18)	C44—N33—C34—C35	179.15 (18)
N2—N3—C4—S1	177.82 (15)	N32—N33—C34—S31	174.50 (15)
C14—N3—C4—S1	-3.9 (3)	C44—N33—C34—S31	-4.6 (3)
Hg1—S1—C4—N3	-175.17 (18)	Hg1—S31—C34—N33	-175.31 (17)
Hg1—S1—C4—C5	4.3 (2)	Hg1—S31—C34—C35	0.0 (2)
N2—O1—C5—N6	174.66 (19)	N32—O31—C35—N36	174.45 (19)
N2—O1—C5—C4	-1.6 (2)	N32—O31—C35—C34	-3.2 (2)
N3—C4—C5—N6	-174.31 (19)	N33—C34—C35—N36	-174.75 (19)
S1—C4—C5—N6	6.1 (3)	S31—C34—C35—N36	9.2 (3)

N3—C4—C5—O1	2.0 (2)	N33—C34—C35—O31	2.9 (2)
S1—C4—C5—O1	-177.60 (15)	S31—C34—C35—O31	-173.14 (16)
O1—C5—N6—C7	17.6 (3)	O31—C35—N36—C37	15.8 (3)
C4—C5—N6—C7	-166.86 (19)	C34—C35—N36—C37	-167.0 (2)
O1—C5—N6—Hg1	172.50 (17)	O31—C35—N36—Hg1	171.41 (17)
C4—C5—N6—Hg1	-11.9 (2)	C34—C35—N36—Hg1	-11.4 (2)
S31—Hg1—N6—C5	167.44 (11)	S31—Hg1—N36—C35	8.19 (12)
S1—Hg1—N6—C5	10.52 (12)	S1—Hg1—N36—C35	164.64 (11)
N36—Hg1—N6—C5	-115.30 (13)	N6—Hg1—N36—C35	-117.26 (13)
S31—Hg1—N6—C7	-40.02 (18)	S31—Hg1—N36—C37	161.00 (17)
S1—Hg1—N6—C7	163.06 (16)	S1—Hg1—N36—C37	-42.55 (18)
N36—Hg1—N6—C7	37.24 (16)	N6—Hg1—N36—C37	35.55 (17)
C5—N6—C7—O7	6.6 (3)	C35—N36—C37—O37	6.1 (3)
Hg1—N6—C7—O7	-141.66 (17)	Hg1—N36—C37—O37	-142.70 (17)
C5—N6—C7—C8	-174.34 (18)	C35—N36—C37—C38	-175.31 (18)
Hg1—N6—C7—C8	37.4 (2)	Hg1—N36—C37—C38	35.9 (2)
O7—C7—C8—C13	-141.0 (2)	O37—C37—C38—C39	-144.9 (2)
N6—C7—C8—C13	39.9 (3)	N36—C37—C38—C39	36.4 (3)
O7—C7—C8—C9	35.4 (3)	O37—C37—C38—C43	33.0 (3)
N6—C7—C8—C9	-143.73 (19)	N36—C37—C38—C43	-145.63 (19)
C13—C8—C9—C10	-1.1 (3)	C43—C38—C39—C40	-0.3 (3)
C7—C8—C9—C10	-177.59 (19)	C37—C38—C39—C40	177.69 (19)
C8—C9—C10—C11	2.2 (3)	C38—C39—C40—C41	0.9 (3)
C9—C10—C11—C12	-1.2 (4)	C39—C40—C41—C42	-0.4 (3)
C10—C11—C12—C13	-0.9 (4)	C40—C41—C42—C43	-0.9 (3)
C9—C8—C13—C12	-1.0 (4)	C41—C42—C43—C38	1.5 (3)
C7—C8—C13—C12	175.4 (2)	C39—C38—C43—C42	-1.0 (3)
C11—C12—C13—C8	2.0 (4)	C37—C38—C43—C42	-178.97 (19)
N2—N3—C14—C15	-118.6 (2)	N32—N33—C44—C45	-54.4 (3)
C4—N3—C14—C15	63.2 (3)	C34—N33—C44—C45	124.7 (2)
N2—N3—C14—C19	63.9 (3)	N32—N33—C44—C49	124.4 (2)
C4—N3—C14—C19	-114.3 (2)	C34—N33—C44—C49	-56.5 (3)
C19—C14—C15—C16	-0.1 (3)	C49—C44—C45—C46	0.6 (3)
N3—C14—C15—C16	-177.49 (19)	N33—C44—C45—C46	179.39 (19)
C14—C15—C16—C17	1.1 (3)	C44—C45—C46—C47	0.9 (3)
C15—C16—C17—C18	-1.2 (3)	C45—C46—C47—C48	-1.1 (4)
C16—C17—C18—C19	0.3 (3)	C46—C47—C48—C49	-0.4 (4)
C17—C18—C19—O20	-179.25 (19)	C47—C48—C49—O50	-176.6 (2)
C17—C18—C19—C14	0.7 (3)	C47—C48—C49—C44	1.8 (3)
C15—C14—C19—O20	179.12 (18)	C45—C44—C49—O50	176.61 (19)

N3—C14—C19—O20	-3.4 (3)	N33—C44—C49—O50	-2.2 (3)
C15—C14—C19—C18	-0.8 (3)	C45—C44—C49—C48	-2.0 (3)
N3—C14—C19—C18	176.60 (17)	N33—C44—C49—C48	179.23 (18)
C18—C19—O20—C21	-10.4 (3)	C48—C49—O50—C51	-17.2 (3)
C14—C19—O20—C21	169.64 (18)	C44—C49—O50—C51	164.29 (18)

Hydrogen-bond geometry (Å, º) for **28a**

D—H···*A*	*D*—H	H···*A*	*D*···*A*	*D*—H···*A*
C21—H21*A*···O37^{i}	0.98	2.43	3.379 (3)	162
C21—H21*C*···N2^{ii}	0.98	2.59	3.557 (3)	170
C45—H45···N32^{iii}	0.95	2.65	3.542 (3)	156
C51—H51*C*···O37^{iv}	0.98	2.62	3.514 (3)	153

Symmetry codes: (i) *x*+1, *y*-1, *z*; (ii) -*x*+2, -*y*, -*z*+2; (iii) -*x*, -*y*+2, -*z*+1; (iv) -*x*+1, -*y*+2, -*z*+1.

Result of the single crystal X-ray analysis of 29

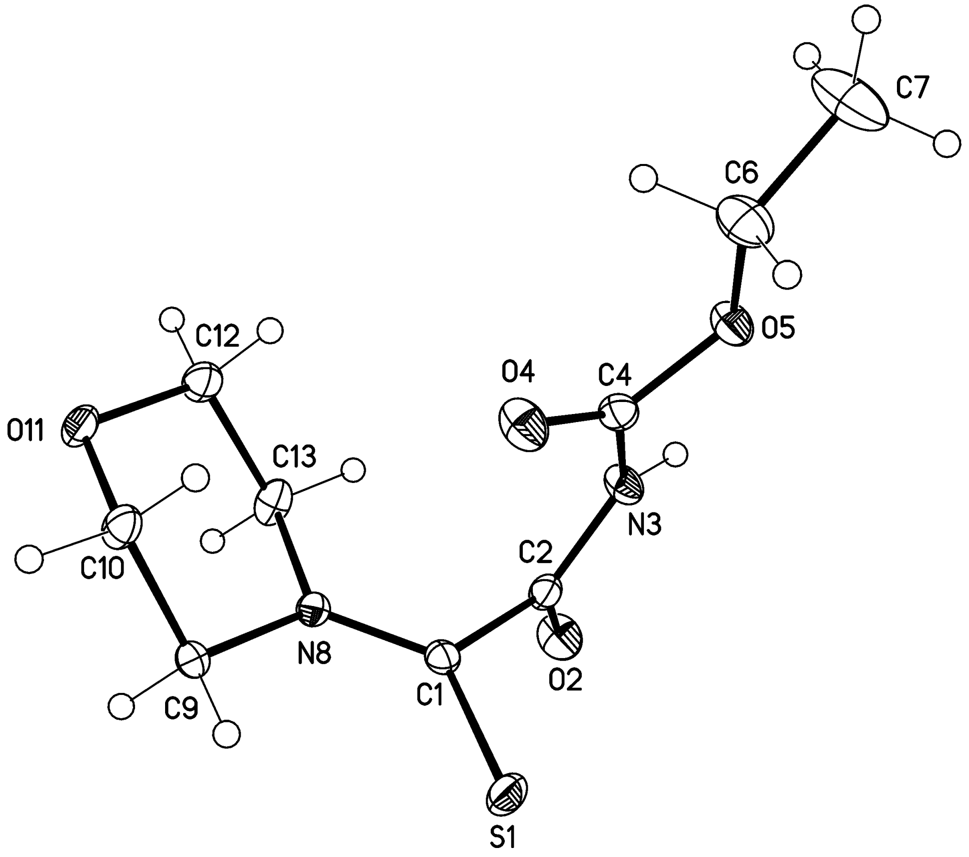

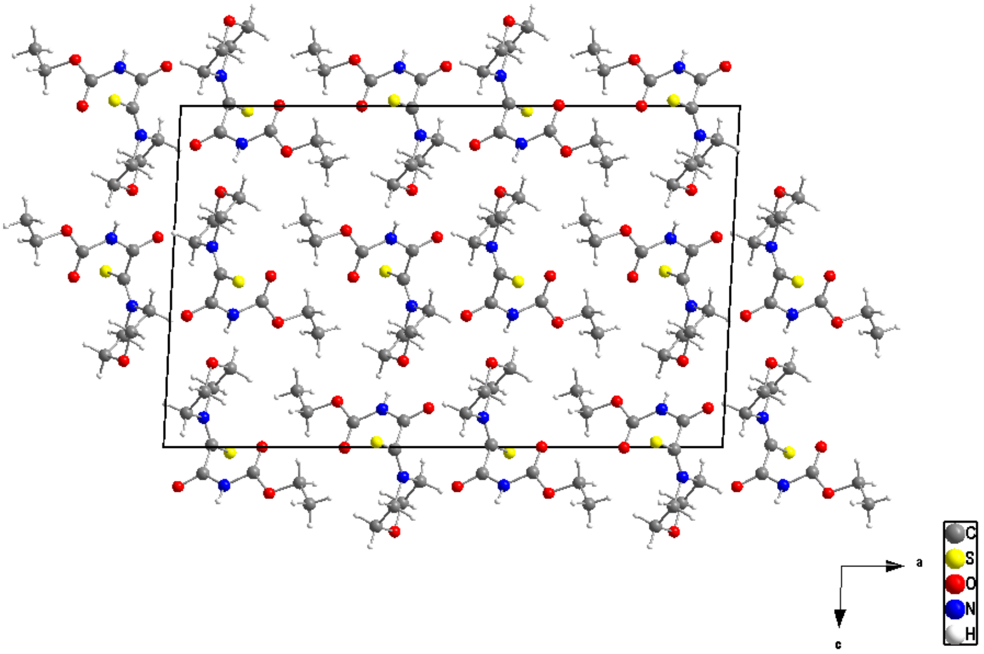

ethyl (2-morpholino-2-thioxoacetyl)carbamate – 29

Crystal data for **29**

$C_9H_{14}N_2O_4S$	*F*(000) = 1040
M_r = 246.28	D_x = 1.426 Mg m^{-3}
Monoclinic, *C*2/*c (no.15)*	Cu *K*α radiation, λ = 1.54178 Å
a = 23.9648 (12) Å	Cell parameters from 9893 reflections
b = 6.5378 (3) Å	θ = 3.7–72.1°
c = 14.6598 (8) Å	μ = 2.56 mm^{-1}
β = 92.902 (1)°	*T* = 123 K
V = 2293.9 (2) Å^3	Blocks, colourless
Z = 8	0.45 × 0.35 × 0.15 mm

Data collection for **29**

Bruker D8 VENTURE diffractometer with Photon100 detector	2240 independent reflections
Radiation source: INCOATEC microfocus sealed tube	2230 reflections with $I > 2\sigma(I)$
Detector resolution: 10.4167 pixels mm^{-1}	R_{int} = 0.023
rotation in ϕ and ω, 1°, shutterless scans	θ_{max} = 72.2°, θ_{min} = 3.7°
Absorption correction: multi-scan *SADABS* (Sheldrick, 2014)	*h* = -29→29
T_{min} = 0.487, T_{max} = 0.697	*k* = -8→7
14352 measured reflections	*l* = -18→15

Refinement for **29**

Refinement on F^2	Secondary atom site location: difference Fourier map
Least-squares matrix: full	Hydrogen site location: difference Fourier map
$R[F^2 > 2\sigma(F^2)]$ = 0.029	H atoms treated by a mixture of independent and constrained refinement
$wR(F^2)$ = 0.073	$w = 1/[\sigma^2(F_o^2) + (0.0329P)^2 + 2.6048P]$ where $P = (F_o^2 + 2F_c^2)/3$
S = 1.09	$(\Delta/\sigma)_{max}$ = 0.001
2240 reflections	$\Delta\rangle_{max}$ = 0.36 e Å^{-3}
149 parameters	$\Delta\rangle_{min}$ = -0.27 e Å^{-3}
1 restraint	Extinction correction: *SHELXL2014*/7 (Sheldrick 2014, $Fc^*=kFc[1+0.001xFc^2\lambda^3/\sin(2\theta)]^{-1/4}$
Primary atom site location: structure-invariant direct methods	Extinction coefficient: 0.00204 (13)

Computing details

Data collection: *APEX3*; cell refinement: *APEX3*; data reduction: *SAINT*; program(s) used to solve structure: *SHELXS97*; program(s) used to refine structure: *SHELXL2014*/7 (Sheldrick,

2014); software used to prepare material for publication: *publCIF*.

Special details for **29**

Experimental. dx = 40 mm, 1 deg., 14+1 runs, 2190 frames, 12/18/24 sec./frame
Geometry. All esds (except the esd in the dihedral angle between two l.s. planes) are estimated using the full covariance matrix. The cell esds are taken into account individually in the estimation of esds in distances, angles and torsion angles; correlations between esds in cell parameters are only used when they are defined by crystal symmetry. An approximate (isotropic) treatment of cell esds is used for estimating esds involving l.s. planes.

Fractional atomic coordinates and isotropic or equivalent isotropic displacement parameters (Å^2) for **29**

	x	*y*	*z*	U_{iso}*/U_{eq}
C1	0.08627 (5)	0.42167 (18)	0.49312 (8)	0.0122 (2)
S1	0.11941 (2)	0.64148 (5)	0.51512 (2)	0.01956 (12)
C2	0.07274 (5)	0.29887 (18)	0.57690 (8)	0.0129 (2)
O2	0.02941 (4)	0.32681 (15)	0.61498 (6)	0.0201 (2)
N3	0.11208 (4)	0.16342 (16)	0.61129 (7)	0.0146 (2)
H3	0.1030 (6)	0.097 (2)	0.6579 (10)	0.018*
C4	0.16277 (5)	0.11672 (18)	0.57337 (8)	0.0139 (3)
O4	0.17622 (4)	0.16659 (15)	0.49879 (6)	0.0204 (2)
O5	0.19309 (4)	0.00609 (14)	0.63420 (6)	0.0173 (2)
C6	0.24680 (5)	-0.0642 (2)	0.60360 (10)	0.0228 (3)
H6A	0.2743	0.0491	0.6064	0.027*
H6B	0.2427	-0.1137	0.5398	0.027*
C7	0.26590 (7)	-0.2338 (3)	0.66589 (12)	0.0376 (4)
H7A	0.3020	-0.2852	0.6474	0.056*
H7B	0.2698	-0.1827	0.7287	0.056*
H7C	0.2384	-0.3448	0.6624	0.056*
N8	0.06899 (4)	0.34705 (15)	0.41310 (7)	0.0119 (2)
C9	0.08219 (5)	0.44404 (19)	0.32665 (8)	0.0148 (3)
H9A	0.1060	0.5657	0.3388	0.018*
H9B	0.0473	0.4891	0.2934	0.018*
C10	0.11263 (5)	0.29090 (19)	0.26919 (8)	0.0156 (3)
H10A	0.1211	0.3543	0.2101	0.019*
H10B	0.1485	0.2523	0.3012	0.019*
O11	0.07913 (4)	0.11140 (13)	0.25295 (6)	0.0158 (2)
C12	0.06849 (5)	0.01314 (19)	0.33752 (8)	0.0169 (3)
H12A	0.1044	-0.0296	0.3682	0.020*
H12B	0.0457	-0.1111	0.3252	0.020*

C13	0.03789 (5)	0.15485 (19)	0.40019 (8)	0.0162 (3)
H13A	0.0000	0.1839	0.3732	0.019*
H13B	0.0339	0.0877	0.4600	0.019*

Atomic displacement parameters (Å^2) for **29**

	U^{11}	U^{22}	U^{33}	U^{12}	U^{13}	U^{23}
C1	0.0132 (5)	0.0124 (6)	0.0110 (5)	0.0031 (4)	0.0015 (4)	0.0002 (4)
S1	0.0333 (2)	0.01299 (18)	0.01233 (18)	-0.00659 (12)	0.00009 (12)	-0.00138 (10)
C2	0.0159 (6)	0.0126 (6)	0.0103 (5)	-0.0009 (4)	0.0008 (4)	-0.0016 (4)
O2	0.0178 (4)	0.0258 (5)	0.0175 (5)	0.0045 (4)	0.0073 (3)	0.0029 (4)
N3	0.0166 (5)	0.0160 (5)	0.0116 (5)	0.0015 (4)	0.0038 (4)	0.0053 (4)
C4	0.0148 (6)	0.0113 (6)	0.0154 (6)	-0.0012 (4)	-0.0004 (4)	0.0002 (4)
O4	0.0185 (4)	0.0260 (5)	0.0171 (5)	0.0040 (4)	0.0051 (3)	0.0057 (4)
O5	0.0157 (4)	0.0199 (5)	0.0163 (4)	0.0041 (3)	0.0003 (3)	0.0029 (3)
C6	0.0151 (6)	0.0265 (7)	0.0268 (7)	0.0055 (5)	0.0030 (5)	0.0038 (6)
C7	0.0271 (8)	0.0413 (10)	0.0450 (10)	0.0164 (7)	0.0084 (7)	0.0189 (8)
N8	0.0144 (5)	0.0113 (5)	0.0100 (5)	0.0008 (4)	0.0016 (4)	0.0000 (4)
C9	0.0233 (6)	0.0118 (6)	0.0094 (5)	0.0012 (5)	0.0010 (4)	0.0018 (4)
C10	0.0209 (6)	0.0147 (6)	0.0115 (5)	-0.0035 (5)	0.0039 (4)	-0.0010 (5)
O11	0.0218 (4)	0.0144 (4)	0.0113 (4)	-0.0025 (3)	0.0023 (3)	-0.0034 (3)
C12	0.0232 (6)	0.0125 (6)	0.0149 (6)	-0.0034 (5)	0.0019 (5)	-0.0002 (5)
C13	0.0163 (6)	0.0192 (7)	0.0134 (6)	-0.0068 (5)	0.0028 (5)	-0.0023 (5)

Geometric parameters (Å, º) for **29**

C1—N8	1.3177 (16)	C7—H7C	0.9800
C1—C2	1.5161 (16)	N8—C9	1.4659 (15)
C1—S1	1.6656 (12)	N8—C13	1.4683 (15)
C2—O2	1.2174 (15)	C9—C10	1.5187 (17)
C2—N3	1.3707 (16)	C9—H9A	0.9900
N3—C4	1.3951 (16)	C9—H9B	0.9900
N3—H3	0.847 (13)	C10—O11	1.4350 (15)
C4—O4	1.2004 (16)	C10—H10A	0.9900
C4—O5	1.3345 (15)	C10—H10B	0.9900
O5—C6	1.4587 (15)	O11—C12	1.4307 (15)
C6—C7	1.494 (2)	C12—C13	1.5193 (17)
C6—H6A	0.9900	C12—H12A	0.9900
C6—H6B	0.9900	C12—H12B	0.9900
C7—H7A	0.9800	C13—H13A	0.9900
C7—H7B	0.9800	C13—H13B	0.9900

N8—C1—C2	117.05 (10)	C9—N8—C13	112.90 (9)
N8—C1—S1	128.10 (9)	N8—C9—C10	108.97 (10)
C2—C1—S1	114.80 (8)	N8—C9—H9A	109.9
O2—C2—N3	121.00 (11)	C10—C9—H9A	109.9
O2—C2—C1	120.95 (11)	N8—C9—H9B	109.9
N3—C2—C1	117.96 (10)	C10—C9—H9B	109.9
C2—N3—C4	126.25 (10)	H9A—C9—H9B	108.3
C2—N3—H3	115.3 (11)	O11—C10—C9	110.53 (10)
C4—N3—H3	118.3 (11)	O11—C10—H10A	109.5
O4—C4—O5	126.41 (11)	C9—C10—H10A	109.5
O4—C4—N3	125.65 (11)	O11—C10—H10B	109.5
O5—C4—N3	107.93 (10)	C9—C10—H10B	109.5
C4—O5—C6	115.10 (10)	H10A—C10—H10B	108.1
O5—C6—C7	107.07 (11)	C12—O11—C10	110.25 (9)
O5—C6—H6A	110.3	O11—C12—C13	111.28 (10)
C7—C6—H6A	110.3	O11—C12—H12A	109.4
O5—C6—H6B	110.3	C13—C12—H12A	109.4
C7—C6—H6B	110.3	O11—C12—H12B	109.4
H6A—C6—H6B	108.6	C13—C12—H12B	109.4
C6—C7—H7A	109.5	H12A—C12—H12B	108.0
C6—C7—H7B	109.5	N8—C13—C12	110.03 (10)
H7A—C7—H7B	109.5	N8—C13—H13A	109.7
C6—C7—H7C	109.5	C12—C13—H13A	109.7
H7A—C7—H7C	109.5	N8—C13—H13B	109.7
H7B—C7—H7C	109.5	C12—C13—H13B	109.7
C1—N8—C9	122.53 (10)	H13A—C13—H13B	108.2
C1—N8—C13	124.48 (10)		
N8—C1—C2—O2	-89.47 (14)	S1—C1—N8—C9	6.45 (17)
S1—C1—C2—O2	88.20 (13)	C2—C1—N8—C13	-0.03 (16)
N8—C1—C2—N3	93.96 (13)	S1—C1—N8—C13	-177.35 (9)
S1—C1—C2—N3	-88.37 (12)	C1—N8—C9—C10	122.21 (12)
O2—C2—N3—C4	179.16 (12)	C13—N8—C9—C10	-54.39 (13)
C1—C2—N3—C4	-4.28 (18)	N8—C9—C10—O11	58.12 (13)
C2—N3—C4—O4	-10.8 (2)	C9—C10—O11—C12	-61.61 (12)
C2—N3—C4—O5	169.71 (11)	C10—O11—C12—C13	59.66 (13)
O4—C4—O5—C6	-2.15 (18)	C1—N8—C13—C12	-123.79 (12)
N3—C4—O5—C6	177.32 (10)	C9—N8—C13—C12	52.73 (13)
C4—O5—C6—C7	-162.61 (12)	O11—C12—C13—N8	-54.50 (13)

C2—C1—N8—C9	-176.23 (10)		

Hydrogen-bond geometry (Å, º) for **29**

D—H···*A*	*D*—H	H···*A*	*D*···*A*	*D*—H···*A*
N3—H3···O11^{i}	0.85 (1)	2.05 (1)	2.8862 (13)	169 (2)
C9—H9*A*···S1	0.99	2.64	3.1379 (12)	112
C9—H9*B*···O2^{ii}	0.99	2.62	3.2189 (15)	119
C10—H10*A*···S1^{iii}	0.99	2.86	3.7624 (12)	152
C12—H12*B*···O2^{iv}	0.99	2.48	3.3307 (15)	144
C13—H13*A*···O11^{v}	0.99	2.62	3.5139 (15)	150

Symmetry codes: (i) *x*, -*y*, *z*+1/2; (ii) -*x*, -*y*+1, -*z*+1; (iii) *x*, -*y*+1, *z*-1/2; (iv) -*x*, -*y*, -*z*+1; (v) -*x*, *y*, -*z*+1/2.

Result of the single crystal X-ray analysis of 30a

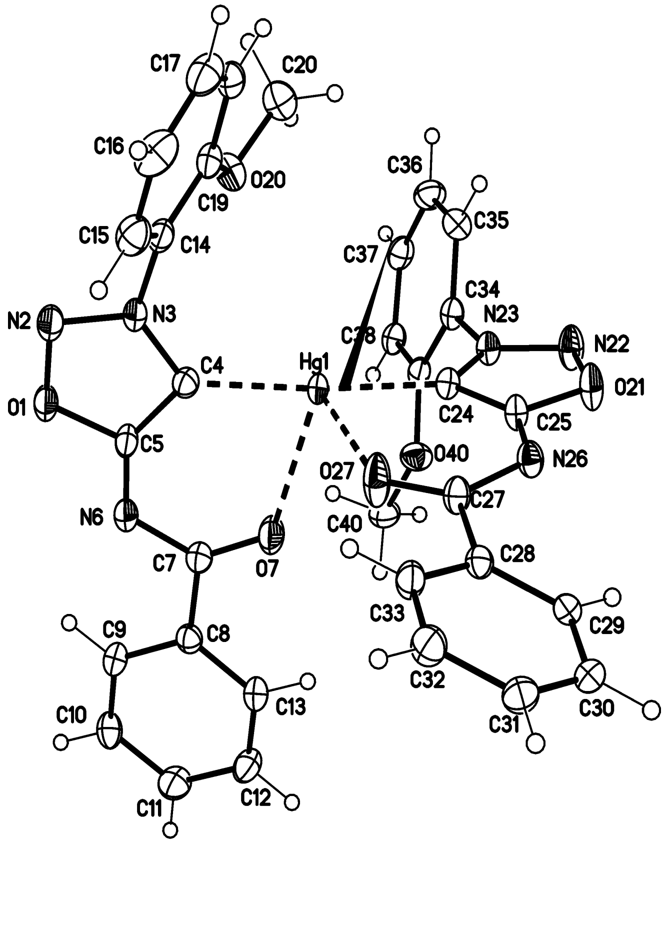

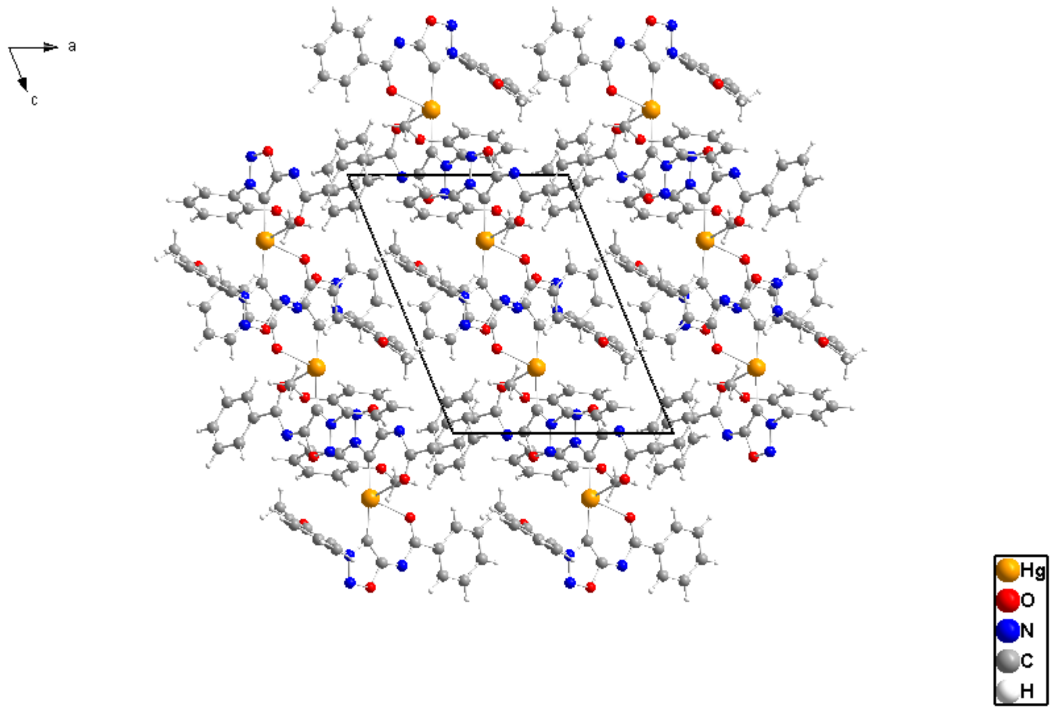

Bis(6-benzoyl-3-(2-methoxyphenyl)-sydnone imine-4-yl)-mercury(II) - 30a

*Crystal data for **30a***

$C_{32}H_{24}HgN_6O_6$	$Z = 2$
$M_r = 789.16$	$F(000) = 772$
Triclinic, *P*-1 (no.2)	$D_x = 1.783$ Mg m^{-3}
$a = 10.4627$ (5) Å	Mo *K*α radiation, λ = 0.71073 Å
$b = 11.4660$ (6) Å	Cell parameters from 9975 reflections
$c = 13.4682$ (7) Å	θ = 2.6–27.5°
α = 79.335 (2)°	μ = 5.29 mm^{-1}
β = 68.151 (2)°	$T = 123$ K
γ = 89.637 (2)°	Blocks, colourless
$V = 1470.28$ (13) Å^3	0.04 × 0.02 × 0.01 mm

*Data collection for **30a***

Bruker D8 VENTURE diffractometer with PhotonII CPAD detector	6372 reflections with $I > 2\sigma(I)$
Radiation source: INCOATEC microfocus sealed tube	$R_{int} = 0.044$
rotation in ϕ and ω, 1°, shutterless scans	$\theta_{max} = 27.5°$, $\theta_{min} = 2.2°$
Absorption correction: multi-scan *SADABS* (Sheldrick, 2014)	$h = -13 \rightarrow 13$

T_{min} = 0.865, T_{max} = 0.942	k = -14→14
55028 measured reflections	l = -17→17
6727 independent reflections	

*Refinement for **30a***

Refinement on F^2	Primary atom site location: dual
Least-squares matrix: full	Secondary atom site location: difference Fourier map
$R[F^2 > 2\sigma(F^2)]$ = 0.024	Hydrogen site location: difference Fourier map
$wR(F^2)$ = 0.050	H-atom parameters constrained
S = 1.19	$w = 1/[\sigma^2(F_o^2) + (0.005P)^2 + 3.850P]$ where $P = (F_o^2 + 2F_c^2)/3$
6727 reflections	$(\Delta/\sigma)_{max}$ = 0.002
408 parameters	$\Delta\rangle_{max}$ = 1.66 e Å^{-3}
0 restraints	$\Delta\rangle_{min}$ = -2.23 e Å^{-3}

Computing details

Data collection: *APEX3*; cell refinement: *APEX3*; data reduction: *SAINT*; program(s) used to solve structure: SHELXT; program(s) used to refine structure: *SHELXL2014*/7 (Sheldrick, 2014); software used to prepare material for publication: *publCIF*.

*Special details for **30a***

Experimental. dx = 40 mm, 1 deg., 5+1 runs, 1126 frames, 60 sec./frame
Geometry. All esds (except the esd in the dihedral angle between two l.s. planes) are estimated using the full covariance matrix. The cell esds are taken into account individually in the estimation of esds in distances, angles and torsion angles; correlations between esds in cell parameters are only used when they are defined by crystal symmetry. An approximate (isotropic) treatment of cell esds is used for estimating esds involving l.s. planes.

*Fractional atomic coordinates and isotropic or equivalent isotropic displacement parameters (Å^2) for **30a***

	x	y	z	U_{iso}*/U_{eq}
Hg1	0.50136 (2)	0.34246 (2)	0.25360 (2)	0.01582 (4)
O1	0.3377 (3)	0.2759 (3)	0.59927 (19)	0.0321 (6)
N2	0.2535 (3)	0.3620 (3)	0.5802 (2)	0.0315 (7)
N3	0.3011 (3)	0.3920 (3)	0.4734 (2)	0.0209 (6)
C4	0.4096 (3)	0.3330 (3)	0.4187 (3)	0.0192 (6)
C5	0.4355 (3)	0.2568 (3)	0.5021 (3)	0.0221 (7)
N6	0.5219 (3)	0.1768 (3)	0.5142 (2)	0.0228 (6)

C7	0.6241 (3)	0.1535 (3)	0.4243 (3)	0.0207 (7)
O7	0.6466 (3)	0.1985 (3)	0.3273 (2)	0.0341 (6)
C8	0.7183 (3)	0.0638 (3)	0.4481 (3)	0.0195 (6)
C9	0.6882 (3)	-0.0034 (3)	0.5530 (3)	0.0216 (7)
H9	0.6063	0.0084	0.6111	0.026*
C10	0.7756 (4)	-0.0860 (3)	0.5727 (3)	0.0254 (7)
H10	0.7538	-0.1315	0.6441	0.031*
C11	0.8956 (4)	-0.1032 (3)	0.4887 (3)	0.0287 (8)
H11	0.9559	-0.1608	0.5022	0.034*
C12	0.9274 (4)	-0.0360 (3)	0.3850 (3)	0.0277 (8)
H12	1.0104	-0.0467	0.3275	0.033*
C13	0.8393 (4)	0.0465 (3)	0.3646 (3)	0.0234 (7)
H13	0.8615	0.0917	0.2930	0.028*
C14	0.2372 (3)	0.4895 (3)	0.4292 (3)	0.0209 (7)
C15	0.2543 (4)	0.6011 (3)	0.4504 (3)	0.0285 (8)
H15	0.3091	0.6128	0.4907	0.034*
C16	0.1907 (4)	0.6949 (3)	0.4120 (3)	0.0329 (9)
H16	0.2027	0.7724	0.4243	0.039*
C17	0.1089 (4)	0.6744 (3)	0.3554 (3)	0.0315 (9)
H17	0.0630	0.7384	0.3308	0.038*
C18	0.0924 (4)	0.5636 (3)	0.3337 (3)	0.0246 (7)
H18	0.0365	0.5522	0.2942	0.029*
C19	0.1579 (3)	0.4689 (3)	0.3700 (3)	0.0207 (7)
O20	0.1508 (3)	0.3565 (2)	0.3534 (2)	0.0261 (5)
C20	0.0734 (4)	0.3347 (3)	0.2888 (3)	0.0303 (8)
H20A	-0.0229	0.3524	0.3247	0.045*
H20B	0.0771	0.2511	0.2818	0.045*
H20C	0.1134	0.3858	0.2163	0.045*
O21	0.6802 (3)	0.3999 (2)	-0.09195 (19)	0.0276 (6)
N22	0.5952 (3)	0.3008 (3)	-0.0722 (2)	0.0271 (7)
N23	0.5379 (3)	0.2744 (2)	0.0351 (2)	0.0187 (5)
C24	0.5759 (3)	0.3471 (3)	0.0892 (3)	0.0174 (6)
C25	0.6734 (3)	0.4291 (3)	0.0052 (3)	0.0195 (6)
N26	0.7592 (3)	0.5197 (2)	-0.0077 (2)	0.0200 (6)
C27	0.7669 (3)	0.5497 (3)	0.0828 (3)	0.0202 (7)
O27	0.6923 (3)	0.5095 (2)	0.1791 (2)	0.0353 (7)
C28	0.8785 (3)	0.6436 (3)	0.0597 (3)	0.0185 (6)
C29	0.9918 (3)	0.6677 (3)	-0.0398 (3)	0.0193 (6)
H29	0.9989	0.6237	-0.0947	0.023*
C30	1.0934 (3)	0.7550 (3)	-0.0588 (3)	0.0227 (7)

H30	1.1708	0.7702	-0.1260	0.027*
C31	1.0816 (4)	0.8206 (3)	0.0213 (3)	0.0266 (8)
H31	1.1502	0.8816	0.0082	0.032*
C32	0.9697 (4)	0.7968 (3)	0.1201 (3)	0.0272 (8)
H32	0.9620	0.8414	0.1747	0.033*
C33	0.8694 (4)	0.7083 (3)	0.1392 (3)	0.0237 (7)
H33	0.7937	0.6916	0.2074	0.028*
C34	0.4423 (3)	0.1698 (3)	0.0806 (3)	0.0180 (6)
C35	0.3166 (4)	0.1749 (3)	0.0690 (3)	0.0240 (7)
H35	0.2908	0.2474	0.0368	0.029*
C36	0.2286 (4)	0.0729 (3)	0.1048 (3)	0.0264 (7)
H36	0.1411	0.0748	0.0986	0.032*
C37	0.2698 (3)	-0.0315 (3)	0.1497 (3)	0.0235 (7)
H37	0.2110	-0.1020	0.1714	0.028*
C38	0.3949 (3)	-0.0368 (3)	0.1641 (3)	0.0200 (7)
H38	0.4197	-0.1093	0.1970	0.024*
C39	0.4836 (3)	0.0662 (3)	0.1293 (3)	0.0180 (6)
O40	0.6079 (2)	0.0729 (2)	0.1376 (2)	0.0247 (5)
C40	0.6524 (4)	-0.0335 (3)	0.1867 (3)	0.0278 (8)
H40A	0.5808	-0.0663	0.2580	0.042*
H40B	0.7380	-0.0146	0.1960	0.042*
H40C	0.6688	-0.0921	0.1395	0.042*

*Atomic displacement parameters (Å^2) for **30a***

	U^{11}	U^{22}	U^{33}	U^{12}	U^{13}	U^{23}
Hg1	0.01763 (6)	0.01677 (6)	0.01029 (6)	-0.00004 (4)	-0.00255 (4)	-0.00175 (4)
O1	0.0317 (14)	0.0480 (17)	0.0141 (12)	0.0179 (13)	-0.0061 (10)	-0.0062 (11)
N2	0.0317 (17)	0.045 (2)	0.0160 (14)	0.0175 (15)	-0.0070 (13)	-0.0070 (13)
N3	0.0211 (14)	0.0260 (15)	0.0141 (13)	0.0029 (12)	-0.0048 (11)	-0.0044 (11)
C4	0.0174 (15)	0.0213 (16)	0.0173 (16)	-0.0004 (13)	-0.0047 (12)	-0.0034 (13)
C5	0.0209 (16)	0.0293 (18)	0.0139 (15)	0.0012 (14)	-0.0031 (13)	-0.0061 (13)
N6	0.0235 (14)	0.0282 (15)	0.0152 (13)	0.0040 (12)	-0.0060 (11)	-0.0036 (12)
C7	0.0203 (16)	0.0225 (17)	0.0177 (16)	0.0003 (13)	-0.0059 (13)	-0.0033 (13)
O7	0.0343 (15)	0.0453 (16)	0.0160 (12)	0.0177 (13)	-0.0047 (11)	-0.0009 (11)
C8	0.0204 (16)	0.0208 (16)	0.0180 (16)	-0.0008 (13)	-0.0078 (13)	-0.0043 (13)
C9	0.0220 (16)	0.0207 (16)	0.0177 (16)	0.0001 (13)	-0.0028 (13)	-0.0032 (13)
C10	0.0324 (19)	0.0221 (17)	0.0178 (16)	0.0003 (15)	-0.0069 (14)	0.0005 (13)
C11	0.0282 (19)	0.0250 (18)	0.031 (2)	0.0087 (15)	-0.0103 (16)	-0.0016 (15)
C12	0.0229 (17)	0.0307 (19)	0.0245 (18)	0.0049 (15)	-0.0025 (14)	-0.0067 (15)
C13	0.0252 (17)	0.0250 (17)	0.0168 (16)	0.0001 (14)	-0.0051 (13)	-0.0023 (13)

C14	0.0201 (16)	0.0222 (16)	0.0163 (15)	0.0043 (13)	-0.0027 (13)	-0.0030 (13)
C15	0.0265 (18)	0.0297 (19)	0.0291 (19)	0.0005 (15)	-0.0077 (15)	-0.0112 (16)
C16	0.032 (2)	0.0191 (18)	0.042 (2)	0.0015 (15)	-0.0052 (17)	-0.0114 (16)
C17	0.031 (2)	0.0228 (18)	0.030 (2)	0.0090 (15)	-0.0019 (16)	0.0001 (15)
C18	0.0239 (17)	0.0277 (18)	0.0169 (16)	0.0014 (14)	-0.0039 (13)	-0.0002 (14)
C19	0.0216 (16)	0.0213 (16)	0.0146 (15)	0.0004 (13)	-0.0022 (12)	-0.0023 (12)
O20	0.0353 (14)	0.0229 (12)	0.0235 (13)	0.0023 (11)	-0.0145 (11)	-0.0050 (10)
C20	0.034 (2)	0.0292 (19)	0.0281 (19)	-0.0019 (16)	-0.0134 (16)	-0.0037 (15)
O21	0.0401 (15)	0.0252 (13)	0.0131 (11)	-0.0132 (11)	-0.0059 (10)	-0.0014 (10)
N22	0.0373 (17)	0.0227 (15)	0.0164 (14)	-0.0124 (13)	-0.0053 (12)	-0.0013 (12)
N23	0.0227 (14)	0.0170 (13)	0.0143 (13)	-0.0023 (11)	-0.0051 (11)	-0.0020 (10)
C24	0.0201 (15)	0.0165 (15)	0.0143 (15)	0.0010 (12)	-0.0047 (12)	-0.0037 (12)
C25	0.0242 (16)	0.0199 (16)	0.0126 (15)	-0.0009 (13)	-0.0051 (12)	-0.0024 (12)
N26	0.0246 (14)	0.0177 (13)	0.0157 (13)	-0.0043 (11)	-0.0062 (11)	-0.0011 (11)
C27	0.0241 (17)	0.0174 (16)	0.0161 (15)	-0.0017 (13)	-0.0047 (13)	-0.0019 (12)
O27	0.0437 (16)	0.0388 (16)	0.0159 (12)	-0.0235 (13)	-0.0032 (11)	-0.0032 (11)
C28	0.0193 (15)	0.0186 (15)	0.0158 (15)	-0.0003 (12)	-0.0063 (12)	0.0000 (12)
C29	0.0194 (15)	0.0232 (16)	0.0162 (15)	0.0035 (13)	-0.0080 (13)	-0.0036 (13)
C30	0.0167 (15)	0.0297 (18)	0.0187 (16)	-0.0012 (13)	-0.0048 (13)	-0.0013 (14)
C31	0.0212 (17)	0.0314 (19)	0.0262 (18)	-0.0062 (15)	-0.0087 (14)	-0.0030 (15)
C32	0.0265 (18)	0.0320 (19)	0.0253 (18)	-0.0026 (15)	-0.0095 (15)	-0.0113 (15)
C33	0.0220 (16)	0.0301 (19)	0.0171 (16)	-0.0038 (14)	-0.0051 (13)	-0.0052 (14)
C34	0.0218 (16)	0.0160 (15)	0.0149 (15)	-0.0034 (12)	-0.0047 (12)	-0.0041 (12)
C35	0.0247 (17)	0.0258 (18)	0.0233 (17)	0.0026 (14)	-0.0114 (14)	-0.0044 (14)
C36	0.0202 (17)	0.0311 (19)	0.0286 (19)	-0.0005 (14)	-0.0095 (14)	-0.0069 (15)
C37	0.0224 (17)	0.0230 (17)	0.0213 (17)	-0.0053 (14)	-0.0036 (13)	-0.0052 (14)
C38	0.0225 (16)	0.0174 (15)	0.0172 (15)	-0.0006 (13)	-0.0041 (13)	-0.0036 (12)
C39	0.0174 (15)	0.0205 (16)	0.0146 (15)	-0.0009 (12)	-0.0036 (12)	-0.0049 (12)
O40	0.0205 (12)	0.0227 (12)	0.0298 (13)	-0.0014 (10)	-0.0113 (10)	0.0014 (10)
C40	0.0220 (17)	0.0315 (19)	0.0285 (19)	0.0075 (15)	-0.0100 (15)	-0.0016 (15)

*Geometric parameters (Å, º) for **30a***

Hg1—C24	2.046 (3)	C20—H20B	0.9800
Hg1—C4	2.050 (3)	C20—H20C	0.9800
Hg1—O7	2.551 (3)	O21—N22	1.371 (4)
Hg1—O27	2.552 (2)	O21—C25	1.386 (4)
O1—N2	1.371 (4)	N22—N23	1.317 (4)
O1—C5	1.383 (4)	N23—C24	1.351 (4)
N2—N3	1.313 (4)	N23—C34	1.450 (4)
N3—C4	1.356 (4)	C24—C25	1.402 (4)

N3—C14	1.446 (4)	C25—N26	1.320 (4)
C4—C5	1.399 (5)	N26—C27	1.357 (4)
C5—N6	1.315 (4)	C27—O27	1.238 (4)
N6—C7	1.355 (4)	C27—C28	1.497 (4)
C7—O7	1.245 (4)	C28—C33	1.387 (5)
C7—C8	1.494 (5)	C28—C29	1.401 (4)
C8—C13	1.388 (5)	C29—C30	1.384 (5)
C8—C9	1.399 (5)	C29—H29	0.9500
C9—C10	1.372 (5)	C30—C31	1.395 (5)
C9—H9	0.9500	C30—H30	0.9500
C10—C11	1.385 (5)	C31—C32	1.388 (5)
C10—H10	0.9500	C31—H31	0.9500
C11—C12	1.383 (5)	C32—C33	1.384 (5)
C11—H11	0.9500	C32—H32	0.9500
C12—C13	1.378 (5)	C33—H33	0.9500
C12—H12	0.9500	C34—C35	1.381 (5)
C13—H13	0.9500	C34—C39	1.394 (4)
C14—C15	1.388 (5)	C35—C36	1.387 (5)
C14—C19	1.395 (5)	C35—H35	0.9500
C15—C16	1.382 (5)	C36—C37	1.379 (5)
C15—H15	0.9500	C36—H36	0.9500
C16—C17	1.387 (6)	C37—C38	1.391 (5)
C16—H16	0.9500	C37—H37	0.9500
C17—C18	1.380 (5)	C38—C39	1.400 (4)
C17—H17	0.9500	C38—H38	0.9500
C18—C19	1.389 (5)	C39—O40	1.350 (4)
C18—H18	0.9500	O40—C40	1.437 (4)
C19—O20	1.355 (4)	C40—H40A	0.9800
O20—C20	1.443 (4)	C40—H40B	0.9800
C20—H20A	0.9800	C40—H40C	0.9800
C24—Hg1—C4	174.81 (13)	O20—C20—H20C	109.5
C24—Hg1—O7	105.40 (10)	H20A—C20—H20C	109.5
C4—Hg1—O7	77.41 (11)	H20B—C20—H20C	109.5
C24—Hg1—O27	77.17 (11)	N22—O21—C25	110.6 (2)
C4—Hg1—O27	107.24 (11)	N23—N22—O21	103.1 (2)
O7—Hg1—O27	92.59 (10)	N22—N23—C24	116.4 (3)
N2—O1—C5	110.7 (3)	N22—N23—C34	115.6 (3)
N3—N2—O1	103.4 (3)	C24—N23—C34	128.0 (3)
N2—N3—C4	116.0 (3)	N23—C24—C25	103.3 (3)

N2—N3—C14	115.7 (3)	N23—C24—Hg1	128.1 (2)
C4—N3—C14	128.2 (3)	C25—C24—Hg1	128.6 (2)
N3—C4—C5	103.5 (3)	N26—C25—O21	113.9 (3)
N3—C4—Hg1	128.0 (2)	N26—C25—C24	139.6 (3)
C5—C4—Hg1	128.3 (2)	O21—C25—C24	106.5 (3)
N6—C5—O1	114.1 (3)	C25—N26—C27	118.2 (3)
N6—C5—C4	139.5 (3)	O27—C27—N26	127.4 (3)
O1—C5—C4	106.4 (3)	O27—C27—C28	118.4 (3)
C5—N6—C7	119.0 (3)	N26—C27—C28	114.2 (3)
O7—C7—N6	127.2 (3)	C27—O27—Hg1	128.3 (2)
O7—C7—C8	118.5 (3)	C33—C28—C29	119.2 (3)
N6—C7—C8	114.3 (3)	C33—C28—C27	119.0 (3)
C7—O7—Hg1	127.8 (2)	C29—C28—C27	121.8 (3)
C13—C8—C9	118.8 (3)	C30—C29—C28	120.5 (3)
C13—C8—C7	119.6 (3)	C30—C29—H29	119.7
C9—C8—C7	121.6 (3)	C28—C29—H29	119.7
C10—C9—C8	120.6 (3)	C29—C30—C31	119.6 (3)
C10—C9—H9	119.7	C29—C30—H30	120.2
C8—C9—H9	119.7	C31—C30—H30	120.2
C9—C10—C11	120.2 (3)	C32—C31—C30	120.1 (3)
C9—C10—H10	119.9	C32—C31—H31	120.0
C11—C10—H10	119.9	C30—C31—H31	120.0
C12—C11—C10	119.6 (3)	C33—C32—C31	120.1 (3)
C12—C11—H11	120.2	C33—C32—H32	120.0
C10—C11—H11	120.2	C31—C32—H32	120.0
C13—C12—C11	120.4 (3)	C32—C33—C28	120.6 (3)
C13—C12—H12	119.8	C32—C33—H33	119.7
C11—C12—H12	119.8	C28—C33—H33	119.7
C12—C13—C8	120.3 (3)	C35—C34—C39	122.5 (3)
C12—C13—H13	119.8	C35—C34—N23	118.6 (3)
C8—C13—H13	119.8	C39—C34—N23	118.8 (3)
C15—C14—C19	122.0 (3)	C34—C35—C36	119.1 (3)
C15—C14—N3	118.1 (3)	C34—C35—H35	120.4
C19—C14—N3	119.9 (3)	C36—C35—H35	120.4
C16—C15—C14	119.2 (4)	C37—C36—C35	119.1 (3)
C16—C15—H15	120.4	C37—C36—H36	120.4
C14—C15—H15	120.4	C35—C36—H36	120.4
C15—C16—C17	119.0 (3)	C36—C37—C38	122.1 (3)
C15—C16—H16	120.5	C36—C37—H37	119.0
C17—C16—H16	120.5	C38—C37—H37	119.0

C18—C17—C16	121.8 (3)	C37—C38—C39	119.1 (3)
C18—C17—H17	119.1	C37—C38—H38	120.4
C16—C17—H17	119.1	C39—C38—H38	120.4
C17—C18—C19	119.8 (3)	O40—C39—C34	117.0 (3)
C17—C18—H18	120.1	O40—C39—C38	125.1 (3)
C19—C18—H18	120.1	C34—C39—C38	117.9 (3)
O20—C19—C18	125.5 (3)	C39—O40—C40	117.6 (3)
O20—C19—C14	116.4 (3)	O40—C40—H40A	109.5
C18—C19—C14	118.1 (3)	O40—C40—H40B	109.5
C19—O20—C20	117.2 (3)	H40A—C40—H40B	109.5
O20—C20—H20A	109.5	O40—C40—H40C	109.5
O20—C20—H20B	109.5	H40A—C40—H40C	109.5
H20A—C20—H20B	109.5	H40B—C40—H40C	109.5
C5—O1—N2—N3	0.3 (4)	C25—O21—N22—N23	1.2 (4)
O1—N2—N3—C4	0.5 (4)	O21—N22—N23—C24	0.4 (4)
O1—N2—N3—C14	-175.5 (3)	O21—N22—N23—C34	-179.1 (3)
N2—N3—C4—C5	-1.1 (4)	N22—N23—C24—C25	-1.7 (4)
C14—N3—C4—C5	174.3 (3)	C34—N23—C24—C25	177.6 (3)
N2—N3—C4—Hg1	173.9 (3)	N22—N23—C24—Hg1	175.6 (2)
C14—N3—C4—Hg1	-10.8 (5)	C34—N23—C24—Hg1	-5.0 (5)
O7—Hg1—C4—N3	178.3 (3)	O7—Hg1—C24—N23	87.4 (3)
O27—Hg1—C4—N3	89.5 (3)	O27—Hg1—C24—N23	176.6 (3)
O7—Hg1—C4—C5	-8.0 (3)	O7—Hg1—C24—C25	-96.0 (3)
O27—Hg1—C4—C5	-96.8 (3)	O27—Hg1—C24—C25	-6.8 (3)
N2—O1—C5—N6	178.7 (3)	N22—O21—C25—N26	176.1 (3)
N2—O1—C5—C4	-1.0 (4)	N22—O21—C25—C24	-2.2 (4)
N3—C4—C5—N6	-178.4 (4)	N23—C24—C25—N26	-175.3 (4)
Hg1—C4—C5—N6	6.7 (7)	Hg1—C24—C25—N26	7.4 (6)
N3—C4—C5—O1	1.2 (4)	N23—C24—C25—O21	2.3 (3)
Hg1—C4—C5—O1	-173.7 (2)	Hg1—C24—C25—O21	-175.0 (2)
O1—C5—N6—C7	-178.3 (3)	O21—C25—N26—C27	-175.6 (3)
C4—C5—N6—C7	1.3 (7)	C24—C25—N26—C27	1.9 (6)
C5—N6—C7—O7	-1.3 (6)	C25—N26—C27—O27	-7.7 (5)
C5—N6—C7—C8	178.3 (3)	C25—N26—C27—C28	173.1 (3)
N6—C7—O7—Hg1	-5.0 (5)	N26—C27—O27—Hg1	4.6 (6)
C8—C7—O7—Hg1	175.5 (2)	C28—C27—O27—Hg1	-176.3 (2)
C24—Hg1—O7—C7	-167.3 (3)	C24—Hg1—O27—C27	2.3 (3)
C4—Hg1—O7—C7	8.2 (3)	C4—Hg1—O27—C27	-174.9 (3)
O27—Hg1—O7—C7	115.3 (3)	O7—Hg1—O27—C27	107.5 (3)

O7—C7—C8—C13	10.0 (5)	O27—C27—C28—C33	-19.9 (5)
N6—C7—C8—C13	-169.6 (3)	N26—C27—C28—C33	159.4 (3)
O7—C7—C8—C9	-170.3 (3)	O27—C27—C28—C29	160.0 (3)
N6—C7—C8—C9	10.0 (5)	N26—C27—C28—C29	-20.8 (5)
C13—C8—C9—C10	-0.9 (5)	C33—C28—C29—C30	-0.1 (5)
C7—C8—C9—C10	179.4 (3)	C27—C28—C29—C30	-180.0 (3)
C8—C9—C10—C11	0.5 (5)	C28—C29—C30—C31	-1.0 (5)
C9—C10—C11—C12	0.5 (6)	C29—C30—C31—C32	1.1 (5)
C10—C11—C12—C13	-1.0 (6)	C30—C31—C32—C33	-0.2 (6)
C11—C12—C13—C8	0.6 (6)	C31—C32—C33—C28	-0.9 (6)
C9—C8—C13—C12	0.4 (5)	C29—C28—C33—C32	1.0 (5)
C7—C8—C13—C12	-179.9 (3)	C27—C28—C33—C32	-179.1 (3)
N2—N3—C14—C15	65.0 (4)	N22—N23—C34—C35	-70.6 (4)
C4—N3—C14—C15	-110.4 (4)	C24—N23—C34—C35	110.0 (4)
N2—N3—C14—C19	-113.2 (4)	N22—N23—C34—C39	106.0 (3)
C4—N3—C14—C19	71.4 (5)	C24—N23—C34—C39	-73.3 (4)
C19—C14—C15—C16	0.2 (5)	C39—C34—C35—C36	-1.3 (5)
N3—C14—C15—C16	-178.0 (3)	N23—C34—C35—C36	175.2 (3)
C14—C15—C16—C17	1.3 (5)	C34—C35—C36—C37	-1.0 (5)
C15—C16—C17—C18	-1.8 (6)	C35—C36—C37—C38	2.6 (5)
C16—C17—C18—C19	0.6 (5)	C36—C37—C38—C39	-1.8 (5)
C17—C18—C19—O20	-179.5 (3)	C35—C34—C39—O40	-178.9 (3)
C17—C18—C19—C14	1.0 (5)	N23—C34—C39—O40	4.6 (4)
C15—C14—C19—O20	179.0 (3)	C35—C34—C39—C38	2.1 (5)
N3—C14—C19—O20	-2.8 (4)	N23—C34—C39—C38	-174.4 (3)
C15—C14—C19—C18	-1.4 (5)	C37—C38—C39—O40	-179.4 (3)
N3—C14—C19—C18	176.8 (3)	C37—C38—C39—C34	-0.5 (5)
C18—C19—O20—C20	2.7 (5)	C34—C39—O40—C40	-179.4 (3)
C14—C19—O20—C20	-177.8 (3)	C38—C39—O40—C40	-0.5 (5)

*Hydrogen-bond geometry (Å, º) for **30a***

D—H···*A*	*D*—H	H···*A*	*D*···*A*	*D*—H···*A*
C20—H20*C*···N26^{i}	0.98	2.64	3.599 (5)	168

Symmetry code: (i) -*x*+1, -*y*+1, -*z*.

Result of the single crystal X-ray analysis of 31a

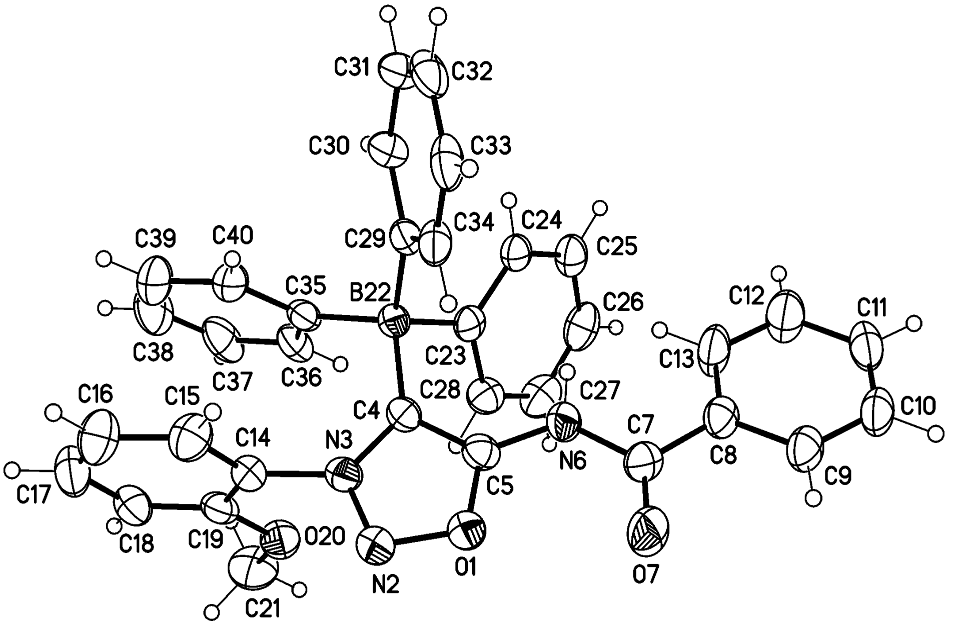

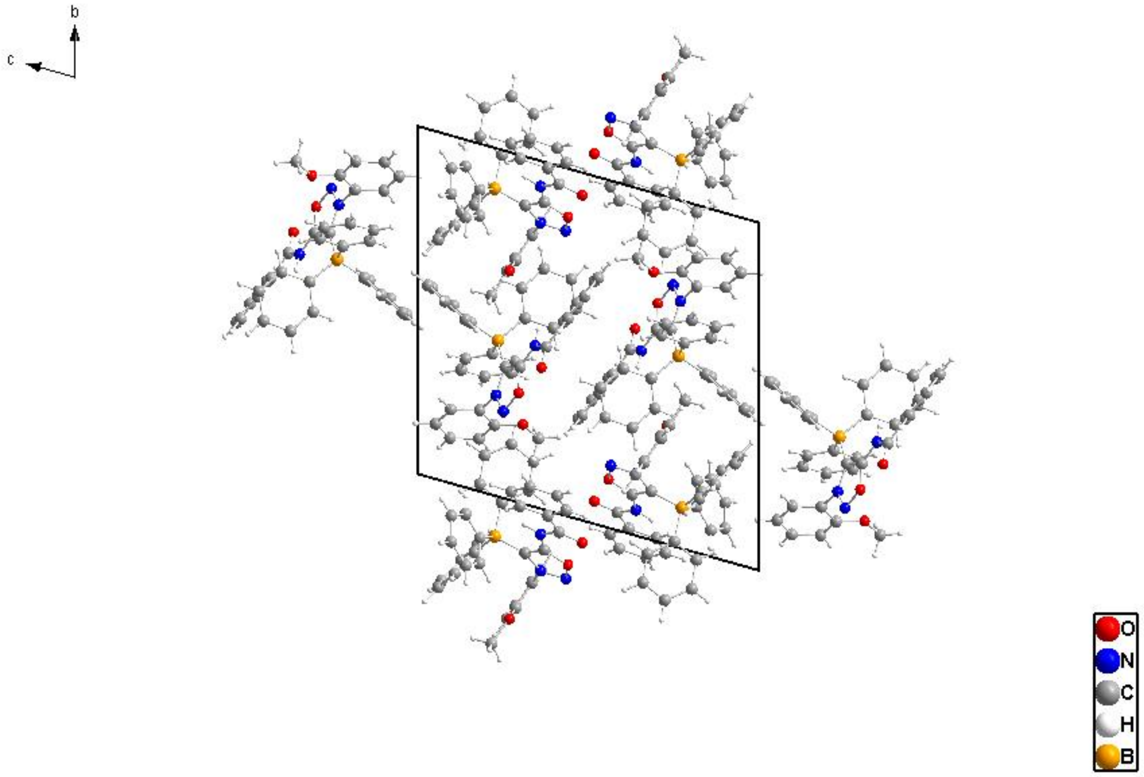

(5-Benzamide-3-(2-methoxyphenyl)-1,2,3-oxadiazol-3-ium-4-yl) triphenylborate - 31a

Crystal data for ***31a***

$C_{34}H_{28}BN_3O_3 \cdot 0.25(C_4H_{10}O)$	$Z = 4$
$M_r = 555.93$	$F(000) = 1170$
Triclinic, *P*-1 (no.2)	$D_x = 1.269$ Mg m^{-3}
$a = 11.2078$ (4) Å	Cu $K\alpha$ radiation, $\lambda = 1.54178$ Å
$b = 16.0505$ (6) Å	Cell parameters from 9835 reflections
$c = 16.8226$ (7) Å	$\theta = 2.8$–$72.1°$
$\alpha = 74.625$ (2)°	$\mu = 0.65$ mm^{-1}
$\beta = 88.792$ (2)°	$T = 123$ K
$\gamma = 85.859$ (2)°	Plates, colourless
$V = 2910.3$ (2) Å^3	0.20 × 0.12 × 0.06 mm

Data collection for ***31a***

Bruker D8 VENTURE diffractometer with PhotonII CPAD detector	9893 reflections with $I > 2\sigma(I)$
Radiation source: INCOATEC microfocus sealed tube	$R_{int} = 0.038$
rotation in ϕ and ω, 1°, shutterless scans	$\theta_{max} = 72.2°$, $\theta_{min} = 2.7°$
Absorption correction: multi-scan *SADABS* (Sheldrick, 2014)	$h = -13 \rightarrow 13$
$T_{min} = 0.819$, $T_{max} = 0.971$	$k = -19 \rightarrow 15$
38234 measured reflections	$l = -20 \rightarrow 20$
11373 independent reflections	

Refinement for ***31a***

Refinement on F^2	Primary atom site location: dual
Least-squares matrix: full	Secondary atom site location: difference Fourier map
$R[F^2 > 2\sigma(F^2)] = 0.060$	Hydrogen site location: inferred from neighbouring sites
$wR(F^2) = 0.172$	H-atom parameters constrained
$S = 1.04$	$w = 1/[\sigma^2(F_o^2) + (0.0813P)^2 + 2.246P]$ where $P = (F_o^2 + 2F_c^2)/3$
11373 reflections	$(\Delta/\sigma)_{max} < 0.001$
741 parameters	$\Delta\rangle_{max} = 0.46$ e Å^{-3}
0 restraints	$\Delta\rangle_{min} = -0.28$ e Å^{-3}

Computing details

Data collection: *APEX3*; cell refinement: *APEX3*; data reduction: *SAINT*; program(s) used to solve structure: SHELXT; program(s) used to refine structure: *SHELXL2014*/7 (Sheldrick,

2014); software used to prepare material for publication: *publCIF*.

Special details for ***31a***

Experimental. dx = 40 mm, 1 deg., 10+1 runs, 1762 frames, 20/50 sec./frame
Geometry. All esds (except the esd in the dihedral angle between two l.s. planes) are estimated using the full covariance matrix. The cell esds are taken into account individually in the estimation of esds in distances, angles and torsion angles; correlations between esds in cell parameters are only used when they are defined by crystal symmetry. An approximate (isotropic) treatment of cell esds is used for estimating esds involving l.s. planes.
Refinement. Refinement with the listed atoms show in one void residual electron density due to a heavily disordered diethylether which could not be refined with split atoms. Therefore the option "SQUEEZE" of the program package PLATON (Spek, A.L. (2009). Acta Cryst. D65, 148-155. Spek, A.L. (2015). Acta Cryst. C71, 9-18.) was used to create a hkl file taking into account the residual electron density in the void areas. Therefore the atoms list and unit card do not agree.

Fractional atomic coordinates and isotropic or equivalent isotropic displacement parameters (Å^2) for ***31a***

	x	*y*	*z*	U_{iso}*/U_{eq}
O1	0.06786 (13)	0.13953 (10)	0.44072 (9)	0.0367 (3)
N2	0.17150 (16)	0.18081 (12)	0.43380 (11)	0.0365 (4)
N3	0.21234 (15)	0.17892 (11)	0.36037 (11)	0.0316 (4)
C4	0.14427 (18)	0.14025 (13)	0.31571 (12)	0.0300 (4)
C5	0.05251 (19)	0.11555 (13)	0.37093 (12)	0.0321 (4)
N6	-0.04504 (16)	0.07357 (12)	0.36081 (11)	0.0356 (4)
H6	-0.0482	0.0584	0.3144	0.043*
C7	-0.1393 (2)	0.05198 (14)	0.41441 (13)	0.0357 (5)
O7	-0.13932 (17)	0.06446 (13)	0.48263 (10)	0.0527 (5)
C8	-0.23981 (19)	0.01433 (14)	0.38165 (14)	0.0361 (5)
C9	-0.3315 (2)	-0.01620 (15)	0.43554 (15)	0.0412 (5)
H9	-0.3283	-0.0132	0.4911	0.049*
C10	-0.4275 (2)	-0.05088 (16)	0.40920 (16)	0.0454 (6)
H10	-0.4893	-0.0720	0.4471	0.054*
C11	-0.4352 (2)	-0.05535 (17)	0.32880 (17)	0.0478 (6)
H11	-0.5022	-0.0787	0.3111	0.057*
C12	-0.3442 (2)	-0.0254 (2)	0.27428 (17)	0.0566 (7)
H12	-0.3480	-0.0288	0.2189	0.068*
C13	-0.2473 (2)	0.00960 (19)	0.30031 (16)	0.0506 (6)
H13	-0.1854	0.0305	0.2624	0.061*
C14	0.32306 (18)	0.22031 (14)	0.33582 (13)	0.0332 (4)
C15	0.4312 (2)	0.17551 (16)	0.36100 (15)	0.0436 (5)
H15	0.4340	0.1175	0.3945	0.052*

C16	0.5356 (2)	0.21651 (19)	0.33663 (18)	0.0528 (6)
H16	0.6110	0.1871	0.3539	0.063*
C17	0.5295 (2)	0.30018 (19)	0.28718 (17)	0.0505 (6)
H17	0.6013	0.3278	0.2699	0.061*
C18	0.4202 (2)	0.34491 (16)	0.26220 (15)	0.0419 (5)
H18	0.4175	0.4025	0.2279	0.050*
C19	0.31506 (19)	0.30519 (14)	0.28757 (13)	0.0341 (4)
O20	0.20309 (14)	0.34176 (10)	0.26964 (10)	0.0414 (4)
C21	0.1915 (3)	0.42772 (17)	0.21660 (19)	0.0564 (7)
H21A	0.2341	0.4664	0.2406	0.085*
H21B	0.1066	0.4478	0.2104	0.085*
H21C	0.2257	0.4279	0.1625	0.085*
B22	0.1651 (2)	0.11690 (14)	0.22628 (14)	0.0288 (4)
C23	0.03199 (18)	0.13300 (13)	0.18251 (12)	0.0300 (4)
C24	-0.01117 (18)	0.07649 (14)	0.14103 (12)	0.0326 (4)
H24	0.0357	0.0247	0.1411	0.039*
C25	-0.1207 (2)	0.09332 (16)	0.09938 (14)	0.0406 (5)
H25	-0.1467	0.0536	0.0714	0.049*
C26	-0.1906 (2)	0.16754 (18)	0.09900 (15)	0.0468 (6)
H26	-0.2643	0.1800	0.0699	0.056*
C27	-0.1526 (2)	0.22408 (17)	0.14144 (15)	0.0465 (6)
H27	-0.2015	0.2745	0.1430	0.056*
C28	-0.0430 (2)	0.20684 (14)	0.18162 (13)	0.0368 (5)
H28	-0.0179	0.2468	0.2096	0.044*
C29	0.21474 (17)	0.01465 (13)	0.24732 (13)	0.0307 (4)
C30	0.2572 (2)	-0.02112 (15)	0.18338 (15)	0.0388 (5)
H30	0.2589	0.0154	0.1288	0.047*
C31	0.2968 (2)	-0.10774 (16)	0.19699 (19)	0.0490 (6)
H31	0.3233	-0.1296	0.1519	0.059*
C32	0.2979 (2)	-0.16221 (15)	0.2760 (2)	0.0512 (7)
H32	0.3253	-0.2214	0.2857	0.061*
C33	0.2588 (2)	-0.12922 (15)	0.33985 (17)	0.0455 (6)
H33	0.2597	-0.1660	0.3944	0.055*
C34	0.21790 (18)	-0.04293 (14)	0.32606 (14)	0.0367 (5)
H34	0.1911	-0.0222	0.3717	0.044*
C35	0.25913 (19)	0.17932 (13)	0.16729 (12)	0.0322 (4)
C36	0.2234 (2)	0.25665 (14)	0.10862 (13)	0.0380 (5)
H36	0.1404	0.2734	0.1014	0.046*
C37	0.3051 (3)	0.30944 (17)	0.06080 (16)	0.0522 (7)
H37	0.2776	0.3610	0.0212	0.063*

C38	0.4260 (3)	0.2873 (2)	0.07063 (19)	0.0634 (8)
H38	0.4821	0.3237	0.0383	0.076*
C39	0.4652 (3)	0.2115 (2)	0.1282 (2)	0.0618 (8)
H39	0.5485	0.1961	0.1356	0.074*
C40	0.3829 (2)	0.15808 (16)	0.17476 (16)	0.0440 (5)
H40	0.4111	0.1057	0.2128	0.053*
O101	0.54927 (13)	0.68579 (9)	0.29651 (9)	0.0353 (3)
N102	0.61362 (16)	0.75056 (11)	0.25144 (12)	0.0359 (4)
N103	0.70940 (14)	0.71016 (10)	0.23007 (10)	0.0278 (3)
C104	0.71652 (17)	0.62163 (12)	0.25683 (12)	0.0265 (4)
C105	0.61027 (17)	0.60943 (13)	0.30028 (12)	0.0291 (4)
N106	0.56304 (15)	0.53530 (11)	0.34475 (11)	0.0329 (4)
H106	0.6091	0.4869	0.3539	0.039*
C107	0.44707 (19)	0.53034 (15)	0.37682 (13)	0.0350 (5)
O107	0.37658 (14)	0.59378 (11)	0.36281 (12)	0.0504 (4)
C108	0.41692 (19)	0.44430 (14)	0.42891 (13)	0.0338 (4)
C109	0.5010 (2)	0.37993 (16)	0.46750 (14)	0.0416 (5)
H109	0.5839	0.3884	0.4599	0.050*
C110	0.4633 (3)	0.30261 (17)	0.51752 (16)	0.0494 (6)
H110	0.5209	0.2583	0.5443	0.059*
C111	0.3434 (3)	0.28975 (17)	0.52854 (15)	0.0484 (6)
H111	0.3185	0.2368	0.5631	0.058*
C112	0.2597 (2)	0.35348 (16)	0.48959 (16)	0.0461 (6)
H112	0.1770	0.3442	0.4968	0.055*
C113	0.2949 (2)	0.43083 (16)	0.44008 (15)	0.0405 (5)
H113	0.2367	0.4748	0.4136	0.049*
C114	0.79535 (18)	0.76651 (13)	0.18223 (13)	0.0305 (4)
C115	0.7909 (2)	0.78774 (17)	0.09766 (14)	0.0460 (6)
H115	0.7315	0.7666	0.0701	0.055*
C116	0.8753 (3)	0.84081 (19)	0.05347 (15)	0.0537 (7)
H116	0.8741	0.8570	-0.0051	0.064*
C117	0.9606 (2)	0.86985 (16)	0.09519 (15)	0.0459 (6)
H117	1.0189	0.9055	0.0646	0.055*
C118	0.9642 (2)	0.84882 (14)	0.18027 (13)	0.0356 (5)
H118	1.0241	0.8698	0.2076	0.043*
C119	0.87935 (18)	0.79676 (12)	0.22539 (12)	0.0286 (4)
O120	0.87000 (13)	0.77206 (10)	0.30872 (9)	0.0336 (3)
C121	0.9587 (2)	0.79954 (18)	0.35446 (15)	0.0464 (6)
H12A	1.0379	0.7756	0.3426	0.070*
H12B	0.9418	0.7789	0.4136	0.070*

H12C	0.9571	0.8629	0.3386	0.070*
B122	0.81864 (19)	0.55106 (14)	0.23510 (13)	0.0262 (4)
C123	0.77118 (18)	0.52101 (12)	0.15591 (11)	0.0275 (4)
C124	0.64989 (19)	0.51709 (13)	0.14030 (12)	0.0320 (4)
H124	0.5922	0.5399	0.1725	0.038*
C125	0.6100 (2)	0.48127 (15)	0.07982 (14)	0.0402 (5)
H125	0.5266	0.4790	0.0722	0.048*
C126	0.6915 (2)	0.44893 (15)	0.03059 (14)	0.0422 (5)
H126	0.6647	0.4250	-0.0113	0.051*
C127	0.8126 (2)	0.45196 (15)	0.04327 (14)	0.0401 (5)
H127	0.8696	0.4309	0.0094	0.048*
C128	0.8507 (2)	0.48584 (13)	0.10561 (13)	0.0341 (4)
H128	0.9342	0.4852	0.1146	0.041*
C129	0.82720 (17)	0.46507 (13)	0.31521 (12)	0.0297 (4)
C130	0.83855 (19)	0.38095 (14)	0.30517 (14)	0.0346 (4)
H130	0.8374	0.3738	0.2509	0.041*
C131	0.8513 (2)	0.30756 (16)	0.37115 (16)	0.0445 (5)
H131	0.8586	0.2518	0.3613	0.053*
C132	0.8535 (2)	0.31507 (18)	0.45053 (16)	0.0515 (6)
H132	0.8634	0.2649	0.4956	0.062*
C133	0.8412 (2)	0.3964 (2)	0.46389 (15)	0.0541 (7)
H133	0.8418	0.4023	0.5186	0.065*
C134	0.8279 (2)	0.46992 (17)	0.39742 (14)	0.0415 (5)
H134	0.8191	0.5253	0.4081	0.050*
C135	0.94857 (18)	0.59372 (13)	0.21910 (14)	0.0317 (4)
C136	1.0209 (2)	0.59694 (14)	0.28537 (17)	0.0418 (5)
H136	0.9935	0.5734	0.3400	0.050*
C137	1.1302 (2)	0.63304 (16)	0.2741 (2)	0.0541 (7)
H137	1.1756	0.6352	0.3205	0.065*
C138	1.1731 (2)	0.66571 (18)	0.1961 (2)	0.0617 (8)
H138	1.2495	0.6887	0.1884	0.074*
C139	1.1047 (3)	0.66518 (19)	0.1284 (2)	0.0617 (8)
H139	1.1338	0.6884	0.0742	0.074*
C140	0.9930 (2)	0.63030 (16)	0.14042 (16)	0.0440 (6)
H140	0.9460	0.6315	0.0937	0.053*

*Atomic displacement parameters (Å^2) for **31a***

	U^{11}	U^{22}	U^{33}	U^{12}	U^{13}	U^{23}
O1	0.0383 (8)	0.0387 (8)	0.0360 (8)	-0.0079 (6)	0.0052 (6)	-0.0138 (6)
N2	0.0369 (9)	0.0382 (10)	0.0381 (9)	-0.0068 (8)	0.0024 (7)	-0.0155 (8)

N3	0.0349 (9)	0.0285 (9)	0.0334 (9)	-0.0029 (7)	0.0010 (7)	-0.0114 (7)
C4	0.0327 (10)	0.0235 (9)	0.0340 (10)	-0.0037 (8)	0.0020 (8)	-0.0075 (8)
C5	0.0394 (11)	0.0277 (10)	0.0295 (10)	-0.0050 (8)	0.0017 (8)	-0.0074 (8)
N6	0.0380 (9)	0.0364 (10)	0.0337 (9)	-0.0116 (8)	0.0074 (7)	-0.0097 (7)
C7	0.0443 (12)	0.0288 (10)	0.0303 (10)	-0.0042 (9)	0.0080 (8)	-0.0018 (8)
O7	0.0568 (10)	0.0673 (12)	0.0371 (9)	-0.0233 (9)	0.0153 (7)	-0.0152 (8)
C8	0.0363 (11)	0.0287 (10)	0.0390 (11)	-0.0039 (8)	0.0053 (9)	-0.0014 (8)
C9	0.0410 (12)	0.0348 (12)	0.0432 (12)	-0.0062 (9)	0.0088 (9)	-0.0020 (9)
C10	0.0369 (12)	0.0373 (12)	0.0573 (15)	-0.0090 (9)	0.0124 (10)	-0.0035 (11)
C11	0.0353 (12)	0.0435 (13)	0.0631 (16)	-0.0133 (10)	0.0018 (10)	-0.0090 (11)
C12	0.0486 (14)	0.077 (2)	0.0465 (14)	-0.0215 (13)	0.0063 (11)	-0.0167 (13)
C13	0.0405 (12)	0.0650 (17)	0.0459 (13)	-0.0221 (12)	0.0132 (10)	-0.0100 (12)
C14	0.0312 (10)	0.0362 (11)	0.0358 (10)	-0.0056 (8)	0.0015 (8)	-0.0151 (9)
C15	0.0391 (12)	0.0418 (13)	0.0456 (13)	0.0027 (10)	-0.0003 (10)	-0.0056 (10)
C16	0.0335 (12)	0.0612 (17)	0.0598 (16)	0.0011 (11)	-0.0044 (11)	-0.0101 (13)
C17	0.0378 (12)	0.0565 (16)	0.0598 (15)	-0.0169 (11)	0.0023 (11)	-0.0164 (12)
C18	0.0449 (12)	0.0362 (12)	0.0479 (13)	-0.0110 (10)	0.0017 (10)	-0.0148 (10)
C19	0.0370 (11)	0.0287 (10)	0.0412 (11)	-0.0010 (8)	-0.0028 (8)	-0.0175 (9)
O20	0.0379 (8)	0.0320 (8)	0.0545 (9)	0.0037 (6)	-0.0018 (7)	-0.0134 (7)
C21	0.0634 (17)	0.0324 (13)	0.0695 (18)	0.0123 (12)	-0.0038 (13)	-0.0102 (12)
B22	0.0311 (11)	0.0248 (11)	0.0310 (11)	-0.0058 (8)	0.0047 (8)	-0.0075 (9)
C23	0.0326 (10)	0.0271 (10)	0.0274 (9)	-0.0052 (8)	0.0069 (7)	-0.0018 (7)
C24	0.0316 (10)	0.0323 (11)	0.0325 (10)	-0.0067 (8)	0.0057 (8)	-0.0056 (8)
C25	0.0368 (11)	0.0506 (14)	0.0343 (11)	-0.0124 (10)	0.0036 (9)	-0.0089 (10)
C26	0.0326 (11)	0.0602 (16)	0.0390 (12)	-0.0002 (10)	-0.0002 (9)	0.0012 (11)
C27	0.0445 (13)	0.0412 (13)	0.0457 (13)	0.0081 (10)	0.0032 (10)	-0.0004 (10)
C28	0.0422 (11)	0.0278 (10)	0.0379 (11)	-0.0005 (9)	0.0022 (9)	-0.0047 (8)
C29	0.0267 (9)	0.0262 (10)	0.0402 (11)	-0.0047 (7)	0.0006 (8)	-0.0102 (8)
C30	0.0424 (12)	0.0305 (11)	0.0462 (12)	-0.0031 (9)	0.0004 (9)	-0.0148 (9)
C31	0.0429 (13)	0.0380 (13)	0.0752 (18)	-0.0025 (10)	0.0016 (12)	-0.0309 (13)
C32	0.0316 (11)	0.0234 (11)	0.097 (2)	-0.0021 (9)	-0.0082 (12)	-0.0130 (12)
C33	0.0298 (10)	0.0320 (12)	0.0653 (16)	-0.0096 (9)	-0.0010 (10)	0.0055 (11)
C34	0.0272 (10)	0.0347 (11)	0.0456 (12)	-0.0068 (8)	0.0026 (8)	-0.0048 (9)
C35	0.0400 (11)	0.0280 (10)	0.0329 (10)	-0.0115 (8)	0.0075 (8)	-0.0139 (8)
C36	0.0511 (13)	0.0327 (11)	0.0322 (10)	-0.0170 (10)	0.0019 (9)	-0.0087 (9)
C37	0.0755 (18)	0.0422 (14)	0.0397 (12)	-0.0308 (13)	0.0046 (12)	-0.0057 (10)
C38	0.0698 (19)	0.0554 (17)	0.0664 (18)	-0.0382 (15)	0.0262 (15)	-0.0116 (14)
C39	0.0444 (14)	0.0625 (18)	0.084 (2)	-0.0224 (13)	0.0259 (14)	-0.0252 (16)
C40	0.0393 (12)	0.0401 (13)	0.0559 (14)	-0.0109 (10)	0.0154 (10)	-0.0177 (11)
O101	0.0317 (7)	0.0279 (7)	0.0462 (8)	-0.0020 (6)	0.0075 (6)	-0.0100 (6)

N102	0.0356 (9)	0.0256 (9)	0.0471 (10)	-0.0026 (7)	0.0075 (8)	-0.0114 (7)
N103	0.0301 (8)	0.0234 (8)	0.0324 (8)	-0.0039 (6)	0.0019 (6)	-0.0111 (6)
C104	0.0287 (9)	0.0241 (9)	0.0288 (9)	-0.0051 (7)	-0.0017 (7)	-0.0095 (7)
C105	0.0289 (9)	0.0259 (10)	0.0337 (10)	-0.0025 (7)	-0.0011 (7)	-0.0102 (8)
N106	0.0322 (9)	0.0274 (9)	0.0389 (9)	-0.0042 (7)	0.0053 (7)	-0.0083 (7)
C107	0.0306 (10)	0.0374 (12)	0.0373 (11)	-0.0086 (9)	-0.0014 (8)	-0.0087 (9)
O107	0.0288 (8)	0.0428 (10)	0.0724 (12)	-0.0028 (7)	0.0006 (7)	-0.0025 (8)
C108	0.0374 (11)	0.0365 (11)	0.0312 (10)	-0.0106 (9)	0.0047 (8)	-0.0137 (9)
C109	0.0402 (12)	0.0448 (13)	0.0388 (11)	-0.0103 (10)	0.0018 (9)	-0.0076 (10)
C110	0.0597 (15)	0.0412 (13)	0.0438 (13)	-0.0092 (11)	-0.0042 (11)	-0.0035 (10)
C111	0.0638 (16)	0.0418 (13)	0.0436 (13)	-0.0238 (12)	0.0115 (11)	-0.0141 (11)
C112	0.0469 (13)	0.0428 (13)	0.0563 (14)	-0.0198 (11)	0.0173 (11)	-0.0233 (11)
C113	0.0385 (11)	0.0396 (12)	0.0496 (13)	-0.0122 (9)	0.0093 (9)	-0.0207 (10)
C114	0.0359 (10)	0.0219 (9)	0.0350 (10)	-0.0061 (8)	0.0062 (8)	-0.0091 (8)
C115	0.0575 (14)	0.0500 (14)	0.0353 (11)	-0.0194 (12)	0.0031 (10)	-0.0158 (10)
C116	0.0745 (18)	0.0592 (16)	0.0290 (11)	-0.0268 (14)	0.0099 (11)	-0.0091 (11)
C117	0.0604 (15)	0.0378 (12)	0.0413 (12)	-0.0217 (11)	0.0149 (11)	-0.0099 (10)
C118	0.0434 (11)	0.0273 (10)	0.0389 (11)	-0.0126 (9)	0.0068 (9)	-0.0113 (8)
C119	0.0344 (10)	0.0195 (9)	0.0329 (10)	-0.0028 (7)	0.0041 (8)	-0.0088 (7)
O120	0.0345 (7)	0.0361 (8)	0.0313 (7)	-0.0114 (6)	0.0014 (6)	-0.0086 (6)
C121	0.0439 (13)	0.0612 (16)	0.0386 (12)	-0.0201 (11)	-0.0017 (10)	-0.0163 (11)
B122	0.0280 (10)	0.0227 (10)	0.0290 (10)	-0.0020 (8)	-0.0012 (8)	-0.0088 (8)
C123	0.0370 (10)	0.0192 (9)	0.0259 (9)	-0.0068 (7)	-0.0005 (7)	-0.0039 (7)
C124	0.0373 (10)	0.0303 (10)	0.0284 (9)	-0.0088 (8)	-0.0006 (8)	-0.0060 (8)
C125	0.0434 (12)	0.0420 (13)	0.0356 (11)	-0.0157 (10)	-0.0055 (9)	-0.0074 (9)
C126	0.0596 (14)	0.0388 (12)	0.0327 (11)	-0.0168 (11)	-0.0032 (10)	-0.0133 (9)
C127	0.0557 (14)	0.0321 (11)	0.0365 (11)	-0.0093 (10)	0.0043 (10)	-0.0145 (9)
C128	0.0398 (11)	0.0277 (10)	0.0376 (11)	-0.0061 (8)	0.0005 (8)	-0.0126 (8)
C129	0.0252 (9)	0.0319 (10)	0.0308 (10)	-0.0003 (7)	-0.0004 (7)	-0.0066 (8)
C130	0.0338 (10)	0.0299 (10)	0.0375 (11)	-0.0021 (8)	-0.0011 (8)	-0.0045 (8)
C131	0.0420 (12)	0.0326 (12)	0.0519 (14)	0.0002 (9)	-0.0039 (10)	0.0006 (10)
C132	0.0452 (13)	0.0508 (15)	0.0447 (13)	0.0048 (11)	-0.0018 (10)	0.0092 (11)
C133	0.0536 (15)	0.0717 (19)	0.0302 (11)	0.0086 (13)	-0.0038 (10)	-0.0049 (11)
C134	0.0434 (12)	0.0471 (13)	0.0325 (11)	0.0077 (10)	-0.0033 (9)	-0.0107 (10)
C135	0.0280 (9)	0.0235 (9)	0.0485 (12)	-0.0008 (7)	0.0028 (8)	-0.0187 (9)
C136	0.0361 (11)	0.0270 (11)	0.0645 (15)	0.0022 (9)	-0.0126 (10)	-0.0158 (10)
C137	0.0358 (12)	0.0358 (13)	0.096 (2)	-0.0016 (10)	-0.0114 (13)	-0.0258 (14)
C138	0.0334 (12)	0.0438 (15)	0.124 (3)	-0.0115 (11)	0.0100 (14)	-0.0478 (17)
C139	0.0615 (17)	0.0493 (15)	0.091 (2)	-0.0283 (13)	0.0429 (16)	-0.0438 (15)
C140	0.0467 (13)	0.0392 (12)	0.0567 (14)	-0.0174 (10)	0.0181 (11)	-0.0287 (11)

Geometric parameters (Å, º) for ***31a***

O1—C5	1.347 (3)	O101—C105	1.347 (2)
O1—N2	1.366 (2)	O101—N102	1.358 (2)
N2—N3	1.315 (2)	N102—N103	1.307 (2)
N3—C4	1.368 (3)	N103—C104	1.369 (3)
N3—C14	1.450 (3)	N103—C114	1.449 (2)
C4—C5	1.379 (3)	C104—C105	1.383 (3)
C4—B22	1.652 (3)	C104—B122	1.656 (3)
C5—N6	1.361 (3)	C105—N106	1.363 (3)
N6—C7	1.377 (3)	N106—C107	1.396 (3)
N6—H6	0.8800	N106—H106	0.8800
C7—O7	1.216 (3)	C107—O107	1.217 (3)
C7—C8	1.497 (3)	C107—C108	1.484 (3)
C8—C9	1.384 (3)	C108—C109	1.380 (3)
C8—C13	1.395 (3)	C108—C113	1.401 (3)
C9—C10	1.379 (4)	C109—C110	1.391 (3)
C9—H9	0.9500	C109—H109	0.9500
C10—C11	1.378 (4)	C110—C111	1.375 (4)
C10—H10	0.9500	C110—H110	0.9500
C11—C12	1.382 (4)	C111—C112	1.374 (4)
C11—H11	0.9500	C111—H111	0.9500
C12—C13	1.388 (4)	C112—C113	1.378 (3)
C12—H12	0.9500	C112—H112	0.9500
C13—H13	0.9500	C113—H113	0.9500
C14—C15	1.378 (3)	C114—C115	1.373 (3)
C14—C19	1.386 (3)	C114—C119	1.390 (3)
C15—C16	1.385 (4)	C115—C116	1.387 (3)
C15—H15	0.9500	C115—H115	0.9500
C16—C17	1.377 (4)	C116—C117	1.375 (4)
C16—H16	0.9500	C116—H116	0.9500
C17—C18	1.388 (4)	C117—C118	1.381 (3)
C17—H17	0.9500	C117—H117	0.9500
C18—C19	1.386 (3)	C118—C119	1.388 (3)
C18—H18	0.9500	C118—H118	0.9500
C19—O20	1.351 (3)	C119—O120	1.356 (2)
O20—C21	1.429 (3)	O120—C121	1.432 (3)
C21—H21A	0.9800	C121—H12A	0.9800
C21—H21B	0.9800	C121—H12B	0.9800
C21—H21C	0.9800	C121—H12C	0.9800
B22—C35	1.637 (3)	B122—C135	1.641 (3)

B22—C29	1.642 (3)	B122—C123	1.643 (3)
B22—C23	1.650 (3)	B122—C129	1.652 (3)
C23—C24	1.399 (3)	C123—C124	1.399 (3)
C23—C28	1.399 (3)	C123—C128	1.406 (3)
C24—C25	1.399 (3)	C124—C125	1.390 (3)
C24—H24	0.9500	C124—H124	0.9500
C25—C26	1.377 (4)	C125—C126	1.385 (4)
C25—H25	0.9500	C125—H125	0.9500
C26—C27	1.388 (4)	C126—C127	1.385 (4)
C26—H26	0.9500	C126—H126	0.9500
C27—C28	1.390 (3)	C127—C128	1.390 (3)
C27—H27	0.9500	C127—H127	0.9500
C28—H28	0.9500	C128—H128	0.9500
C29—C34	1.399 (3)	C129—C130	1.401 (3)
C29—C30	1.407 (3)	C129—C134	1.406 (3)
C30—C31	1.389 (3)	C130—C131	1.389 (3)
C30—H30	0.9500	C130—H130	0.9500
C31—C32	1.383 (4)	C131—C132	1.373 (4)
C31—H31	0.9500	C131—H131	0.9500
C32—C33	1.370 (4)	C132—C133	1.378 (4)
C32—H32	0.9500	C132—H132	0.9500
C33—C34	1.387 (3)	C133—C134	1.396 (4)
C33—H33	0.9500	C133—H133	0.9500
C34—H34	0.9500	C134—H134	0.9500
C35—C36	1.404 (3)	C135—C140	1.395 (3)
C35—C40	1.405 (3)	C135—C136	1.406 (3)
C36—C37	1.387 (3)	C136—C137	1.381 (3)
C36—H36	0.9500	C136—H136	0.9500
C37—C38	1.378 (5)	C137—C138	1.368 (5)
C37—H37	0.9500	C137—H137	0.9500
C38—C39	1.388 (5)	C138—C139	1.389 (5)
C38—H38	0.9500	C138—H138	0.9500
C39—C40	1.388 (3)	C139—C140	1.397 (3)
C39—H39	0.9500	C139—H139	0.9500
C40—H40	0.9500	C140—H140	0.9500
C5—O1—N2	108.42 (15)	C105—O101—N102	108.75 (15)
N3—N2—O1	103.76 (15)	N103—N102—O101	103.96 (15)
N2—N3—C4	116.43 (17)	N102—N103—C104	116.52 (16)
N2—N3—C14	115.67 (16)	N102—N103—C114	114.60 (16)

C4—N3—C14	127.88 (17)	C104—N103—C114	128.87 (16)
N3—C4—C5	100.02 (17)	N103—C104—C105	99.87 (16)
N3—C4—B22	132.88 (17)	N103—C104—B122	128.96 (17)
C5—C4—B22	126.80 (18)	C105—C104—B122	130.96 (17)
O1—C5—N6	120.87 (18)	O101—C105—N106	118.61 (17)
O1—C5—C4	111.38 (18)	O101—C105—C104	110.89 (17)
N6—C5—C4	127.74 (19)	N106—C105—C104	130.49 (19)
C5—N6—C7	127.88 (19)	C105—N106—C107	124.89 (18)
C5—N6—H6	116.1	C105—N106—H106	117.6
C7—N6—H6	116.1	C107—N106—H106	117.6
O7—C7—N6	121.2 (2)	O107—C107—N106	121.0 (2)
O7—C7—C8	123.7 (2)	O107—C107—C108	122.9 (2)
N6—C7—C8	115.09 (19)	N106—C107—C108	116.03 (19)
C9—C8—C13	118.5 (2)	C109—C108—C113	119.7 (2)
C9—C8—C7	117.5 (2)	C109—C108—C107	123.9 (2)
C13—C8—C7	124.02 (19)	C113—C108—C107	116.3 (2)
C10—C9—C8	120.5 (2)	C108—C109—C110	119.5 (2)
C10—C9—H9	119.7	C108—C109—H109	120.3
C8—C9—H9	119.7	C110—C109—H109	120.3
C11—C10—C9	121.1 (2)	C111—C110—C109	120.6 (3)
C11—C10—H10	119.5	C111—C110—H110	119.7
C9—C10—H10	119.5	C109—C110—H110	119.7
C10—C11—C12	119.1 (2)	C112—C111—C110	120.0 (2)
C10—C11—H11	120.4	C112—C111—H111	120.0
C12—C11—H11	120.4	C110—C111—H111	120.0
C11—C12—C13	120.1 (3)	C111—C112—C113	120.4 (2)
C11—C12—H12	119.9	C111—C112—H112	119.8
C13—C12—H12	119.9	C113—C112—H112	119.8
C12—C13—C8	120.6 (2)	C112—C113—C108	119.8 (2)
C12—C13—H13	119.7	C112—C113—H113	120.1
C8—C13—H13	119.7	C108—C113—H113	120.1
C15—C14—C19	122.3 (2)	C115—C114—C119	123.01 (19)
C15—C14—N3	120.0 (2)	C115—C114—N103	119.62 (19)
C19—C14—N3	117.68 (18)	C119—C114—N103	117.37 (17)
C14—C15—C16	118.8 (2)	C114—C115—C116	118.4 (2)
C14—C15—H15	120.6	C114—C115—H115	120.8
C16—C15—H15	120.6	C116—C115—H115	120.8
C17—C16—C15	119.7 (2)	C117—C116—C115	119.4 (2)
C17—C16—H16	120.2	C117—C116—H116	120.3
C15—C16—H16	120.2	C115—C116—H116	120.3

C16—C17—C18	121.2 (2)	C116—C117—C118	122.0 (2)
C16—C17—H17	119.4	C116—C117—H117	119.0
C18—C17—H17	119.4	C118—C117—H117	119.0
C19—C18—C17	119.7 (2)	C117—C118—C119	119.3 (2)
C19—C18—H18	120.2	C117—C118—H118	120.4
C17—C18—H18	120.2	C119—C118—H118	120.4
O20—C19—C18	125.9 (2)	O120—C119—C118	126.08 (18)
O20—C19—C14	115.84 (19)	O120—C119—C114	116.02 (17)
C18—C19—C14	118.3 (2)	C118—C119—C114	117.90 (19)
C19—O20—C21	117.34 (19)	C119—O120—C121	116.91 (16)
O20—C21—H21A	109.5	O120—C121—H12A	109.5
O20—C21—H21B	109.5	O120—C121—H12B	109.5
H21A—C21—H21B	109.5	H12A—C121—H12B	109.5
O20—C21—H21C	109.5	O120—C121—H12C	109.5
H21A—C21—H21C	109.5	H12A—C121—H12C	109.5
H21B—C21—H21C	109.5	H12B—C121—H12C	109.5
C35—B22—C29	111.17 (16)	C135—B122—C123	113.36 (16)
C35—B22—C23	109.90 (16)	C135—B122—C129	109.94 (16)
C29—B22—C23	111.72 (16)	C123—B122—C129	107.88 (15)
C35—B22—C4	111.98 (16)	C135—B122—C104	109.88 (15)
C29—B22—C4	106.56 (16)	C123—B122—C104	108.14 (15)
C23—B22—C4	105.35 (16)	C129—B122—C104	107.43 (15)
C24—C23—C28	115.66 (19)	C124—C123—C128	114.95 (18)
C24—C23—B22	122.74 (18)	C124—C123—B122	122.95 (17)
C28—C23—B22	121.54 (19)	C128—C123—B122	121.53 (18)
C23—C24—C25	122.5 (2)	C125—C124—C123	123.0 (2)
C23—C24—H24	118.7	C125—C124—H124	118.5
C25—C24—H24	118.7	C123—C124—H124	118.5
C26—C25—C24	119.8 (2)	C126—C125—C124	120.1 (2)
C26—C25—H25	120.1	C126—C125—H125	119.9
C24—C25—H25	120.1	C124—C125—H125	119.9
C25—C26—C27	119.5 (2)	C127—C126—C125	119.1 (2)
C25—C26—H26	120.3	C127—C126—H126	120.5
C27—C26—H26	120.3	C125—C126—H126	120.5
C26—C27—C28	119.9 (2)	C126—C127—C128	119.9 (2)
C26—C27—H27	120.0	C126—C127—H127	120.0
C28—C27—H27	120.0	C128—C127—H127	120.0
C27—C28—C23	122.6 (2)	C127—C128—C123	122.9 (2)
C27—C28—H28	118.7	C127—C128—H128	118.5
C23—C28—H28	118.7	C123—C128—H128	118.5

C34—C29—C30	115.1 (2)	C130—C129—C134	115.01 (19)
C34—C29—B22	124.95 (19)	C130—C129—B122	121.48 (18)
C30—C29—B22	119.91 (19)	C134—C129—B122	123.48 (19)
C31—C30—C29	122.5 (2)	C131—C130—C129	122.8 (2)
C31—C30—H30	118.7	C131—C130—H130	118.6
C29—C30—H30	118.7	C129—C130—H130	118.6
C32—C31—C30	120.2 (2)	C132—C131—C130	120.4 (2)
C32—C31—H31	119.9	C132—C131—H131	119.8
C30—C31—H31	119.9	C130—C131—H131	119.8
C33—C32—C31	118.8 (2)	C131—C132—C133	119.1 (2)
C33—C32—H32	120.6	C131—C132—H132	120.4
C31—C32—H32	120.6	C133—C132—H132	120.4
C32—C33—C34	121.0 (2)	C132—C133—C134	120.3 (2)
C32—C33—H33	119.5	C132—C133—H133	119.9
C34—C33—H33	119.5	C134—C133—H133	119.9
C33—C34—C29	122.4 (2)	C133—C134—C129	122.4 (2)
C33—C34—H34	118.8	C133—C134—H134	118.8
C29—C34—H34	118.8	C129—C134—H134	118.8
C36—C35—C40	116.1 (2)	C140—C135—C136	116.0 (2)
C36—C35—B22	123.35 (19)	C140—C135—B122	122.94 (19)
C40—C35—B22	120.51 (19)	C136—C135—B122	121.0 (2)
C37—C36—C35	122.2 (2)	C137—C136—C135	122.5 (3)
C37—C36—H36	118.9	C137—C136—H136	118.7
C35—C36—H36	118.9	C135—C136—H136	118.7
C38—C37—C36	120.1 (3)	C138—C137—C136	120.0 (3)
C38—C37—H37	119.9	C138—C137—H137	120.0
C36—C37—H37	119.9	C136—C137—H137	120.0
C37—C38—C39	119.5 (2)	C137—C138—C139	119.9 (2)
C37—C38—H38	120.2	C137—C138—H138	120.1
C39—C38—H38	120.2	C139—C138—H138	120.1
C38—C39—C40	120.1 (3)	C138—C139—C140	119.7 (3)
C38—C39—H39	120.0	C138—C139—H139	120.2
C40—C39—H39	120.0	C140—C139—H139	120.2
C39—C40—C35	121.9 (3)	C135—C140—C139	121.8 (3)
C39—C40—H40	119.0	C135—C140—H140	119.1
C35—C40—H40	119.0	C139—C140—H140	119.1
C5—O1—N2—N3	-0.2 (2)	C105—O101—N102—N103	0.3 (2)
O1—N2—N3—C4	0.6 (2)	O101—N102—N103—C104	0.2 (2)
O1—N2—N3—C14	179.09 (16)	O101—N102—N103—C114	-178.53 (15)

N2—N3—C4—C5	-0.8 (2)	N102—N103—C104—C105	-0.5 (2)
C14—N3—C4—C5	-179.03 (19)	C114—N103—C104—C105	177.94 (18)
N2—N3—C4—B22	-174.5 (2)	N102—N103—C104—B122	174.65 (18)
C14—N3—C4—B22	7.2 (3)	C114—N103—C104—B122	-6.9 (3)
N2—O1—C5—N6	-179.36 (19)	N102—O101—C105—N106	178.28 (17)
N2—O1—C5—C4	-0.3 (2)	N102—O101—C105—C104	-0.7 (2)
N3—C4—C5—O1	0.6 (2)	N103—C104—C105—O101	0.7 (2)
B22—C4—C5—O1	174.90 (18)	B122—C104—C105—O101	-174.35 (18)
N3—C4—C5—N6	179.6 (2)	N103—C104—C105—N106	-178.1 (2)
B22—C4—C5—N6	-6.1 (4)	B122—C104—C105—N106	6.9 (4)
O1—C5—N6—C7	1.8 (3)	O101—C105—N106—C107	10.4 (3)
C4—C5—N6—C7	-177.1 (2)	C104—C105—N106—C107	-170.8 (2)
C5—N6—C7—O7	-5.3 (4)	C105—N106—C107—O107	3.9 (3)
C5—N6—C7—C8	173.7 (2)	C105—N106—C107—C108	-175.41 (18)
O7—C7—C8—C9	-7.1 (3)	O107—C107—C108—C109	-158.3 (2)
N6—C7—C8—C9	173.9 (2)	N106—C107—C108—C109	21.0 (3)
O7—C7—C8—C13	171.8 (2)	O107—C107—C108—C113	19.9 (3)
N6—C7—C8—C13	-7.2 (3)	N106—C107—C108—C113	-160.83 (19)
C13—C8—C9—C10	0.4 (4)	C113—C108—C109—C110	-0.4 (3)
C7—C8—C9—C10	179.3 (2)	C107—C108—C109—C110	177.6 (2)
C8—C9—C10—C11	-0.6 (4)	C108—C109—C110—C111	0.2 (4)
C9—C10—C11—C12	0.8 (4)	C109—C110—C111—C112	0.3 (4)
C10—C11—C12—C13	-0.8 (4)	C110—C111—C112—C113	-0.7 (4)
C11—C12—C13—C8	0.6 (5)	C111—C112—C113—C108	0.5 (4)
C9—C8—C13—C12	-0.4 (4)	C109—C108—C113—C112	0.1 (3)
C7—C8—C13—C12	-179.3 (3)	C107—C108—C113—C112	-178.1 (2)
N2—N3—C14—C15	83.5 (3)	N102—N103—C114—C115	-93.1 (2)
C4—N3—C14—C15	-98.3 (3)	C104—N103—C114—C115	88.4 (3)
N2—N3—C14—C19	-96.0 (2)	N102—N103—C114—C119	86.8 (2)
C4—N3—C14—C19	82.2 (3)	C104—N103—C114—C119	-91.7 (2)
C19—C14—C15—C16	-0.6 (4)	C119—C114—C115—C116	1.0 (4)
N3—C14—C15—C16	179.9 (2)	N103—C114—C115—C116	-179.1 (2)
C14—C15—C16—C17	-0.9 (4)	C114—C115—C116—C117	0.5 (4)
C15—C16—C17—C18	1.0 (4)	C115—C116—C117—C118	-0.9 (4)
C16—C17—C18—C19	0.3 (4)	C116—C117—C118—C119	0.0 (4)
C17—C18—C19—O20	178.2 (2)	C117—C118—C119—O120	-178.7 (2)
C17—C18—C19—C14	-1.7 (3)	C117—C118—C119—C114	1.3 (3)
C15—C14—C19—O20	-178.1 (2)	C115—C114—C119—O120	178.2 (2)
N3—C14—C19—O20	1.4 (3)	N103—C114—C119—O120	-1.8 (3)
C15—C14—C19—C18	1.9 (3)	C115—C114—C119—C118	-1.8 (3)

N3—C14—C19—C18	-178.64 (19)	N103—C114—C119—C118	178.25 (18)
C18—C19—O20—C21	3.1 (3)	C118—C119—O120—C121	-2.5 (3)
C14—C19—O20—C21	-176.9 (2)	C114—C119—O120—C121	177.5 (2)
N3—C4—B22—C35	-24.0 (3)	N103—C104—B122—C135	31.0 (3)
C5—C4—B22—C35	163.6 (2)	C105—C104—B122—C135	-155.2 (2)
N3—C4—B22—C29	97.7 (2)	N103—C104—B122—C123	-93.2 (2)
C5—C4—B22—C29	-74.6 (2)	C105—C104—B122—C123	80.6 (2)
N3—C4—B22—C23	-143.5 (2)	N103—C104—B122—C129	150.63 (18)
C5—C4—B22—C23	44.2 (3)	C105—C104—B122—C129	-35.7 (3)
C35—B22—C23—C24	101.5 (2)	C135—B122—C123—C124	-152.93 (18)
C29—B22—C23—C24	-22.4 (3)	C129—B122—C123—C124	85.1 (2)
C4—B22—C23—C24	-137.72 (18)	C104—B122—C123—C124	-30.8 (2)
C35—B22—C23—C28	-75.6 (2)	C135—B122—C123—C128	36.1 (2)
C29—B22—C23—C28	160.48 (18)	C129—B122—C123—C128	-85.8 (2)
C4—B22—C23—C28	45.2 (2)	C104—B122—C123—C128	158.24 (17)
C28—C23—C24—C25	1.6 (3)	C128—C123—C124—C125	0.0 (3)
B22—C23—C24—C25	-175.68 (18)	B122—C123—C124—C125	-171.46 (19)
C23—C24—C25—C26	-0.6 (3)	C123—C124—C125—C126	-1.3 (3)
C24—C25—C26—C27	-1.3 (3)	C124—C125—C126—C127	0.8 (3)
C25—C26—C27—C28	2.0 (4)	C125—C126—C127—C128	1.0 (3)
C26—C27—C28—C23	-1.0 (3)	C126—C127—C128—C123	-2.4 (3)
C24—C23—C28—C27	-0.8 (3)	C124—C123—C128—C127	1.9 (3)
B22—C23—C28—C27	176.5 (2)	B122—C123—C128—C127	173.47 (19)
C35—B22—C29—C34	132.1 (2)	C135—B122—C129—C130	-101.3 (2)
C23—B22—C29—C34	-104.8 (2)	C123—B122—C129—C130	22.8 (2)
C4—B22—C29—C34	9.8 (3)	C104—B122—C129—C130	139.18 (18)
C35—B22—C29—C30	-48.6 (2)	C135—B122—C129—C134	76.5 (2)
C23—B22—C29—C30	74.6 (2)	C123—B122—C129—C134	-159.47 (19)
C4—B22—C29—C30	-170.87 (18)	C104—B122—C129—C134	-43.1 (2)
C34—C29—C30—C31	1.4 (3)	C134—C129—C130—C131	-0.9 (3)
B22—C29—C30—C31	-178.0 (2)	B122—C129—C130—C131	177.02 (19)
C29—C30—C31—C32	-1.3 (4)	C129—C130—C131—C132	-0.1 (4)
C30—C31—C32—C33	0.3 (4)	C130—C131—C132—C133	0.9 (4)
C31—C32—C33—C34	0.4 (3)	C131—C132—C133—C134	-0.7 (4)
C32—C33—C34—C29	-0.3 (3)	C132—C133—C134—C129	-0.4 (4)
C30—C29—C34—C33	-0.6 (3)	C130—C129—C134—C133	1.1 (3)
B22—C29—C34—C33	178.71 (19)	B122—C129—C134—C133	-176.7 (2)
C29—B22—C35—C36	148.01 (19)	C123—B122—C135—C140	22.5 (3)
C23—B22—C35—C36	23.8 (3)	C129—B122—C135—C140	143.3 (2)
C4—B22—C35—C36	-92.9 (2)	C104—B122—C135—C140	-98.6 (2)

C29—B22—C35—C40	-33.5 (3)	C123—B122—C135—C136	-158.94 (18)
C23—B22—C35—C40	-157.67 (19)	C129—B122—C135—C136	-38.1 (2)
C4—B22—C35—C40	85.6 (2)	C104—B122—C135—C136	79.9 (2)
C40—C35—C36—C37	-0.2 (3)	C140—C135—C136—C137	-0.7 (3)
B22—C35—C36—C37	178.4 (2)	B122—C135—C136—C137	-179.4 (2)
C35—C36—C37—C38	-0.8 (4)	C135—C136—C137—C138	-1.5 (4)
C36—C37—C38—C39	0.6 (4)	C136—C137—C138—C139	2.2 (4)
C37—C38—C39—C40	0.6 (5)	C137—C138—C139—C140	-0.7 (4)
C38—C39—C40—C35	-1.7 (4)	C136—C135—C140—C139	2.2 (3)
C36—C35—C40—C39	1.4 (3)	B122—C135—C140—C139	-179.1 (2)
B22—C35—C40—C39	-177.2 (2)	C138—C139—C140—C135	-1.5 (4)
